# Evolutionary constraint on a core winter proteome across independently cold-adapted PACMAD grasses

**DOI:** 10.1371/journal.pone.0358458

**Published:** 2026-10-14

**Authors:** Elad Oren, Jingjing Zhai, Travis E. Rooney, Ruthie Angelovici, Charles O. Hale, Lara J. Brindisi, Sheng-Kai Hsu, Christine M. Gault, Jian Hua, Thuy La, Nicholas Lepak, Qin Fu, Edward S. Buckler, Maria Cinta Romay

**Affiliations:** 1 Institute for Genomic Diversity, Cornell University, Ithaca, New York, United States of America; 2 Newe Ya’ar Research Center, Institute of Plant Sciences, Agricultural Research Organization – Volcani Institute, Ramat Yishay, Israel; 3 Sesaco Corporation, Austin, Texas, United States of America; 4 Division of Biological Sciences and Interdisciplinary Plant Group, University of Missouri, Columbia, Missouri, United States of America; 5 School of Integrative Plant Sciences, Plant Breeding and Genetics Section, Cornell University, Ithaca, New York, United States of America; 6 USDA-ARS, Ithaca, New York, United States of America; 7 School of Integrative Plant Science, Plant Biology Section, Cornell University, Ithaca, New York, United States of America; 8 Proteomics and Metabolomics Facility, Cornell University, Ithaca, New York, United States of America; Delaware State University, UNITED STATES OF AMERICA

## Abstract

Grasses in the Panicoideae, Aristidoideae, Chloridoideae, Micrairoideae, Arundinoideae, and Danthonioideae (PACMAD) clade independently colonized cold environments from warm-climate ancestors, but whether their molecular responses to freezing reflect shared evolutionary solutions or lineage-specific innovations remains poorly understood at the protein level. We used comparative proteomics to test whether protein-level cold responses show stronger cross-species conservation than previously observed at the transcript level. We quantified seasonal rhizome proteomes (winter vs summer) from five PACMAD species grown in a common-garden experiment exposed to sustained sub-zero temperatures, identified differentially abundant proteins, and compared fold-change magnitudes across species using orthogroup-based correlation analyses. We further examined LEA3 protein structure through hydropathy profiling and motif analysis. Shared cold-responsive proteins showed higher cross-species fold-change correlation (*ρ* = 0.80) than background proteins (*ρ* = 0.45), despite greater divergence in baseline abundance. This correlation advantage persisted (Δ*ρ* = 0.27) after controlling for effect-size selection by comparing shared cold-responsive proteins against magnitude-matched single-species responses. LEA3 was the only ortholog elevated across all five species, with cold-tolerant species containing more tandem 11-mer repeats than the cold-sensitive maize. Two species accumulated multiple LEA3 paralogs, increasing total LEA3 abundance. Independent evolution of freezing tolerance in PACMAD grasses is consistent with evolutionary constraints on protein-level response magnitude, reflecting the retention of an ancestral protective capacity. Structural divergence of LEA3 in maize suggests that transcriptional induction alone does not ensure freezing tolerance; functional protection likely requires intact motif architecture.

## Introduction

Grasses of the PACMAD clade, including maize, sorghum, sugarcane, the millets, and the bioenergy grass *Miscanthus*, supply a substantial fraction of global cereal, forage, and biofuel production. Maize (*Zea mays* ssp. mays), a vital crop for global food security, is susceptible to cold stress due to its tropical origins, limiting its yield in temperate regions. Early planting of maize can improve nitrogen capture, net primary productivity, and yield [1]. However, this strategy requires cold tolerance, which is lacking in current elite maize varieties [2,3]. Cold and freezing tolerance has arisen independently multiple times within the PACMAD clade (encompassing the Panicoideae, Aristidoideae, Chloridoideae, Micrairoideae, Arundinoideae, and Danthonioideae), a major lineage of predominantly C4 grasses (*Poaceae*) that diverged ~64 Mya, with ancestral roots in the Afrotropics [4,5]*.* These parallel origins of freezing tolerance provide a natural comparative framework. Among these, *Tripsacum*, the closest perennial and cold-tolerant relative of maize, having diverged about 650 Kya [6], is valuable for enhancing maize cold tolerance.

PACMAD grasses originated in warm-season climates unlike temperate diversified Pooideae and Danthonioideae [7]. However, most transitions of PACMAD grasses to colder climates occurred in tropical mountains, where cold-adapted lineages converged with temperate migrants [8]. Each PACMAD genus exhibits distinct evolutionary trajectories, polyploid histories, and biogeographic origins, having independently evolved robust cold and freezing tolerance over the past 20–30 million years (Fig 1e; [5,9]).

**Fig 1:**
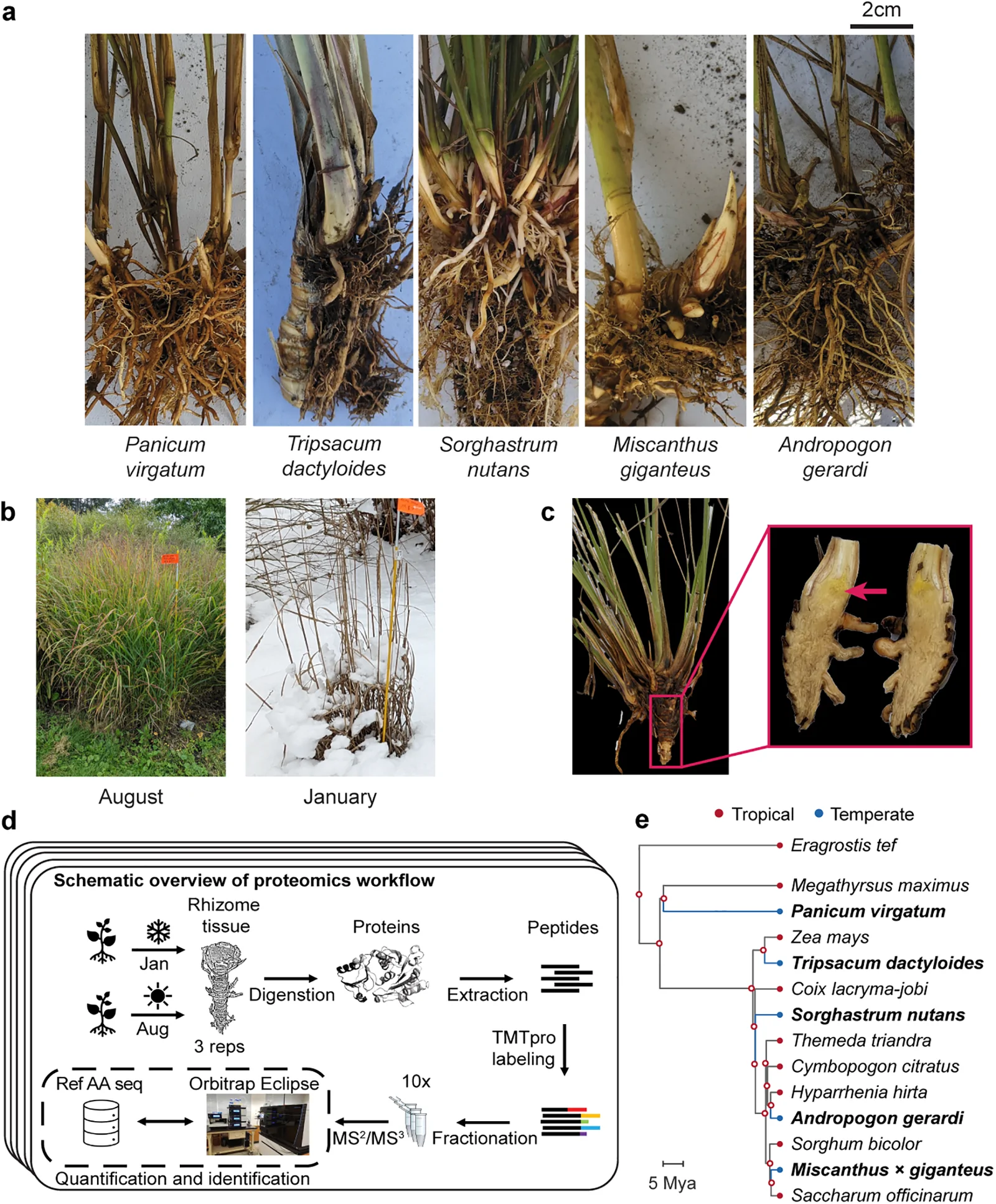
Overview of sampling and proteomics workflow. **(a)** Rhizomes from the five species sampled in the study: *Tripsacum dactyloides* and *T. floridanum* hybrids, *Panicum virgatum*, *Andropogon gerardi*, *Miscanthus × giganteus*, and *Sorghastrum nutans*. **(b)** Field conditions during the two sampling seasons, illustrating the active growth in August and dormancy in January. **(c)** Example of the rhizome tissue sampled for proteomics, shown here for *Tripsacum*. The red arrow highlights the specific region collected. **(d)** Schematic overview of the proteomics workflow. Rhizome samples were collected each season, followed by protein extraction, digestion, and TMTpro labeling. Peptides were fractionated and analyzed via RTS-SPS-MS^3^ using an Orbitrap Eclipse mass spectrometer. Each species’ reference proteome was used for peptide and protein assignment, enabling quantification and identification. **(e)** Phylogenetic relationships among the five study species (bold) and tropical relatives, rooted on *Eragrostis tef* (Chloridoideae). Red, tropical; Blue, temperate and freezing tolerant.

Plant exposure to cold stress, including chilling (>0°C) and freezing (<0°C) temperatures, triggers physiological and molecular responses. Chilling stress disrupts membrane fluidity, impairs enzyme activity, and inhibits photosynthesis [3], whereas freezing stress induces ice crystal formation in extracellular spaces, leading to cellular dehydration, membrane damage, and ultimately, cell death [10]. Many temperate plants undergo cold acclimation, resulting in adaptations such as membrane lipid modifications [11,12], accumulation of compatible solutes [13], enhanced antioxidant enzyme activity [14] and protective protein accumulation [15,16]. Plant cold perception involves plasma membrane modifications, including fluidity changes, Ca² ⁺ channel activation, and cytosolic acidification, driving lipid and transcriptomic responses [17,18]. A widely conserved mechanism underpinning freezing tolerance is the activation of the C-repeat binding factor/dehydration-responsive element-binding (CBF/DREB) signaling pathway, which induces expression of cold-responsive (COR) genes [19,20].

Studies in PACMAD grasses reveal both broadly conserved and species-specific molecular adaptations associated with this pathway. *Panicum virgatum* utilizes a zinc finger transcription factor, PvC3H72, to enhance ICE1-CBF-COR signaling, significantly increasing cold resilience [21]. Additionally, calcium signaling pathways involving calmodulin and MEKK1 provide early response to cold stress [22,23]. *Pennisetum giganteum* maintains membrane fluidity at low temperature through the accumulation of unsaturated fatty acids, such as linoleic and α-linolenic acids [24,25]. Protective proteins, such as late embryogenesis abundant (LEA) proteins, dehydrins, and antioxidant enzymes, are accumulated in response to freezing conditions, reducing oxidative injury and cellular dehydration [21,26]. Species-specific adaptations include enhanced proline biosynthesis via the ornithine pathway in *Sorghum bicolor* [27] and elevated activity of phenylpropanoid pathway enzymes (PAL and CAD) in *Miscanthus* to protect cells during freezing stress [28].

Despite the agronomic and evolutionary interest in cold tolerance across the PACMAD clade, much prior molecular work has focused on the acute leaf-level cold-shock response. Inter-annual survival in perennial PACMAD grasses, however, depends on the rhizome, a specialized below-ground stem that overwinters intact after aerial tissues senesce and gives rise to new aboveground growth each spring. Because the rhizome is buffered by soil from diurnal extremes of the aerial canopy, and because its overwintering is a sustained, months-long state of dormancy rather than a brief acute exposure, the molecular biology of winter survival in these species is fundamentally a question about a dormant subterranean organ and the survival strategy it deploys, not about an immediate stress response. We therefore focus our comparative proteomic analysis on the rhizome as the organ on which inter-annual survival actually depends.

Previous studies in PACMAD grasses have focused mainly on transcriptomic and metabolomic adaptations. However, proteomic responses remain largely unexplored. As the functional products of genes, proteins integrate transcriptional, translational, and post-translational regulation. Given the persistent nature of cold stress, proteomic analysis uniquely captures the accumulation of proteins reflecting acclimation and sustained adaptive responses. Furthermore, Cope et al. [29] demonstrated that proteins face stronger evolutionary constraints than transcripts, with protein abundances being highly conserved across species while mRNA levels can vary without substantially affecting protein output. This suggests that shared cold adaptive responses may be more readily detected at the protein level than at the transcript level, making proteomics essential for understanding adaptive processes such as those underlying cold or freezing tolerance. Here, we test the hypothesis that independently cold-adapted PACMAD lineages converge on a shared core winter proteome, defined as the set of orthogroups differentially abundant between summer-active and winter-dormant rhizomes in two or more of the five species examined. We further hypothesize that the magnitude of cold-induced proteomic responses is consistent with constraint imposed by the shared physiological requirements of freezing tolerance.

## Materials and methods

To investigate proteomic responses to seasonal changes across PACMAD grasses, we conducted a comparative analysis of rhizomes from five species under winter (January, with lows reaching –29°C) and summer (August, with highs of 26°C) conditions. The species analyzed (Fig 1a) were interspecific and intraspecific hybrids of *Tripsacum dactyloides* (L.) L. and *Tripsacum floridanum* Porter ex Vasey, *Andropogon gerardi* Vitman (cv. ‘Sentinel’), *Miscanthus* × *giganteus* J.M.Greef & Deuter ex Hodk. & Renvoize, *Panicum virgatum* L. (cv. ‘Dust Devil’), and *Sorghastrum nutans* (L.) Nash (cv. ‘Golden Sunset’). All taxa sampled belong to the subfamily Panicoideae; the remaining PACMAD subfamilies (Arundinoideae, Chloridoideae, Micrairoideae, Aristidoideae, and Danthonioideae) were not sampled. We use “PACMAD” to situate these independent cold-adaptation events within the broader clade in which freezing tolerance has repeatedly evolved (see Phylogenetic context, below).

### Phylogenetic context of freezing tolerance

To illustrate independent origins of freezing tolerance in each of the five study species rather than from a single common ancestor, we constructed phylogenetic context by pruning the 1,153-tip ASTRAL-Pro nuclear species tree [30] to 14 representative species (Fig 1e): Paniceae (2 species) and Andropogoneae (11 species from 7 subtribes) and *Eragrostis tef* (Chloridoideae) as outgroup. Of these 14, only five temperate species were sampled for proteomics analysis (*P. virgatum*, *T. dactyloides*, *S. nutans*, *A. gerardi*, and *M. giganteus*); the remaining nine tropical relatives (*Megathyrsus maximus*, *Zea mays*, *Coix lacryma-jobi*, *Themeda triandra*, *Cymbopogon citratus*, *Hyparrhenia hirta*, *Sorghum bicolor*, *Saccharum officinarum*) were included to demonstrate that each study species sits on a separate branch from its nearest warm-climate relative, confirming five independent transitions to freezing tolerance. *Sorghastrum fuscescens* served as a positional proxy for *S. nutans*, which was absent from the tree, and *M. giganteus*’s position was determined by its maternal progenitor, *Miscanthus sinensis*. Thermal ecology was classified as tropical or temperate based on native range descriptions in Kellogg [7].

### Plant material and environmental conditions

Plants were grown outdoors at two field sites in Ithaca, New York (USDA Hardiness Zone 6a), where they experienced natural seasonal fluctuations, with an average winter temperature of –5°C (lows reaching –29°C) and summer temperatures averaging 20°C (highs of 26°C) over the five years preceding sampling. *Tripsacum* hybrids were cultivated at Cornell University Caldwell Field, while *A. gerardi, M. giganteus, P. virgatum*, and *S. nutan*s were grown at the Cornell Botanic Gardens. Environmental data were obtained from the Northeast Regional Climate Center (Station USC00304174) and the New York State Mesonet (Station GROT; [31]).

### Sampling and experimental design

Rhizome tissue was collected during two sampling campaigns in 2019–2020 and 2022, timed to capture distinct physiological states: winter dormancy and summer active growth. Summer samples were collected in August 2019 and August 2022 during active growth; winter samples in January 2020 and December 2022 during dormancy. Soil temperatures and precipitation data for each sampling date are reported in S1 Methods.

At summer sampling, plants displayed active vegetative rhizome growth with no visible drought stress; winter samples were dormant with hardened exteriors (S1 Fig). Our study was conducted in two phases. Initial sampling of *Tripsacum* hybrids in 2019 provided evidence of conserved winter protein accumulation, prompting expansion in 2022 to both replicated *Tripsacum* sampling and four additional PACMAD species with independent evolutionary histories of cold adaptation.

Rhizome tissue (minimum 5 g fresh weight) was collected from the internal mid-sections of rhizomes using sterile pruning scissors. Sampling was standardized to exclude apical tips, fine roots, and necrotic zones to ensure reproducibility and tissue comparability across species (Fig 1c and S1 Fig). Samples were immediately placed in pre-labeled 15 ml tubes, transported on dry ice, and stored at −80°C within 4 hours of collection. Biological replicates were defined as tissue collected from independent clonal plants. Sample sizes and genotypes are detailed in Table 1. For *A. gerardi* (’Sentinel’), *M. giganteus*, and *P. virgatum* (’Dust Devil’), three biological replicates were sampled per season from established accessions at Cornell Botanic Gardens. *S. nutans* (’Golden Sunset’) was represented by two biological replicates per season due to winter mortality of one clonal plant.

**Table 1:** Summary of rhizome proteomics datasets for *Tripsacum* hybrids, *Andropogon gerardi*, *Miscanthus* × *giganteus*, *Panicum virgatum*, and *Sorghastrum nutans*, including number of samples per season, total proteins quantified, proteins shared across seasons, and differentially accumulated proteins (DAPs) accumulated (up) or depleted (down) in winter.

Species	Year	N samples	Genotype / Cultivar	Entry code	Clonal reps	Proteins quantified		DAP up	DAP down
						Total	Shared winter/summer		
*Tripsacum spp.*	2019	8	*T. dactyloides ‘IA_Pete_2’ × T. floridanum*	C002	1	4,451	4,451	123	175
			*T. dactyloides ‘KS_C9_9’ × T. floridanum*	C004	1				
			*T. dactyloides ‘KS_B6_1’ × T. floridanum*	C022	3				
			*T. dactyloides ‘KS_C9_10’ × T. floridanum*	C031	1				
			*T. dactyloides ‘IA_Pete_1’ × T. dactyloides ‘FL’*	C071	1				
			*T. dactyloides ‘TX_HC_3’ × T. dactyloides ‘MO_TP’*	C076	1				
*Tripsacum spp.*	2022	24	*T. dactyloides ‘IA_Pete_2’ × T. floridanum*	C002	3	3,468	3,054	100	141
			*T. dactyloides ‘KS_C9_9’ × T. floridanum*	C004	3				
			*T. dactyloides ‘KS_B6_1’ × T. floridanum*	C022	3				
			*T. dactyloides ‘KS_C7_3’ × T. floridanum*	C027	3				
			*T. dactyloides ‘IL_10_1’ × T. floridanum*	C043	3				
			*T. dactyloides ‘IL_14’ × T. dactyloides ‘FL’*	C065	3				
			*T. dactyloides ‘IA_Pete_1’ × T. dactyloides ‘FL’*	C071	3				
			*T. dactyloides ‘TX_HC_3’ × T. dactyloides ‘MO_TP’*	C076	3				
*Andropogon gerardi*	2022	3	Sentinel	01-266*A	3	3,734	3,733	109	76
*Miscanthus* × *giganteus*	2022	3	–	88-363*A	3	2,372	2,273	58	83
*Panicum virgatum*	2022	3	Dust Devil	01-277*B	3	4,071	4,068	174	218
*Sorghastrum nutans*	2022	2	Golden Sunset	22-095*A	2	2,641	2,464	66	56

### Tripsacum hybrid population

*Tripsacum* rhizomes were sampled from seven-year-old F_1_ hybrid progenies that had survived multiple freezing winters at Cornell University’s Caldwell Field. The hybrids were derived from controlled crosses between northern founders collected from Iowa, Kansas, Illinois, and Missouri, where winters experience heavy frost, and southern founders collected from Florida and Texas, with subtropical climates. Most crosses were interspecific, between *T. dactyloides* subsp. *dactyloides* (northern) and *T. floridanum* (southern); additional crosses were intraspecific, between northern and southern populations of *T. dactyloides* (Table 1). In 2019, we sampled eight individuals per season representing six distinct hybrid genotypes from five cross combinations, prioritizing coverage of genetic diversity across the hybrid population. In 2022, we sampled 24 individuals per season comprising eight hybrid genotypes with three clonal replicates each, balancing genetic diversity with robust statistical replication.

### F_2_ Seedlings for RNA sequencing

F_2_ seeds used for seedling freezing tolerance screening and RNA sequencing were generated from the same *Tripsacum* hybrid population described above, generated and screened as part of a companion bulk-segregant mapping study [32]. Due to the limited seed set achieved through self-pollination (~500 seeds over the entire season), F_1_ plants were allowed to open-pollinate. Seeds were harvested from individual maternal plants, germinated in trays, and grouped into F_2_ families based on maternal lineage. Seedlings were grown for approximately four weeks under standard greenhouse conditions, transplanted, and cold-acclimated in a growth chamber for one week before ice-nucleation treatment and freezing exposure; full growth, acclimation, and freezing-screen conditions are described in El-Walid et al. [32]. Six-week-old F_2_ seedlings sharing the same maternal F_1_ parent were selected as experimental material for freezing assays and RNA sampling.

### Proteomics data

Proteomic profiling was conducted using tandem mass tag (TMTpro) labeling and Orbitrap Eclipse Real Time Search (RTS)-SPS-MS^3^ technology. The experimental design included samples collected in both winter (January) and summer (August), with each species represented by at least three biological replicates with a larger number of genotypes analyzed for *Tripsacum* across two years (see Table 1 and Fig 1d).

### Protein extraction and digestion

Fresh rhizome tissue was ground in liquid nitrogen and subjected to phenol-based protein extraction with protease and phosphatase inhibitors, followed by ammonium acetate/methanol precipitation, following Isaacson et al. [33] with modifications. Protein pellets were dissolved in 8 M urea, quantified by BCA assay, and digested using the S-Trap protocol with trypsin as described in Fu et al. [34]. Digested peptide mixtures were labeled using TMTpro reagents: TMTpro 16-plex for 2019 Tripsacum samples and TMTpro 18-plex for 2022 samples. The 2022 samples were processed across seven separate TMTpro 18-plex batches: four batches for Tripsacum and three batches for the remaining four species, with winter and summer samples from each species always included within the same batch. Detailed buffer compositions, wash steps, digestion parameters, and labeling procedures are provided in S1 Methods.

### LC-MS/MS analysis and database searching

Labeled peptides were fractionated by high-pH reversed-phase chromatography (48 fractions concatenated into 10 pools) and analyzed by nanoLC coupled to an Orbitrap Eclipse mass spectrometer using the RTS-SPS-MS^3^ method [34]. Raw data were processed in Proteome Discoverer 2.5 using the Sequest HT search engine against species-specific reference proteomes (see Peptide Mapping and Reference Proteome Assignment). Peptide spectral matches were filtered at 1% FDR (Percolator), and protein identifications required a minimum of two distinct peptides. Protein quantification was based on summed reporter ion intensities of unique and razor peptides. Full search parameters, modification lists, mass tolerances, and reporter ion filtering criteria are detailed in S1 Methods.

Given the inherent biological variability of a multi-species field study, we applied a stringent threshold for differential abundance. Differentially abundant proteins (DAPs) were determined based on |log_2_ fold change| ≥ 1 and adjusted P-value < 0.05, to prioritize high-confidence candidates with robust seasonal responses over minor fluctuations that may reflect transient environmental noise rather than conserved adaptive strategies. This approach helps address the statistical power imbalance between the deeply sampled Tripsacum dataset (n ≈ 32) and the other grass lineages (n = 3). While the higher power in Tripsacum inherently yields a larger list of statistically significant changes, our comparative analysis defines conservation based on the intersection of species. Consequently, the identification of conserved proteins is constrained by the more limited statistical power of the smaller datasets, suggesting that the core response network we report represents a high-confidence set of shared adaptations rather than an exhaustive catalog.

### Peptide mapping and reference proteome assignment

Initial Sequest HT searches were performed against species-specific reference proteomes (see above). For species pooled within the same TMT batch due to <15% predicted proteome divergence, such as *A. gerardi* and *M. giganteus*, spectra were searched against the *A. gerardi* proteome. To improve protein recovery for *M. giganteus*, peptides were re-mapped to the *M. sinensis* proteome using DIAMOND BLASTp in “ultrasensitive” mode. Proteins were reassigned to their best-matching *M. sinensis* counterparts based on aggregated peptide evidence (GitHub repository: https://github.com/elor77/PACMAD-Rhizome-Proteomics). All final analyses were conducted using species-specific reference proteomes: *A. gerardi* (Ag-CAM1351-DRAFT-PanAnd-1.0), *M. giganteus* (Msinensis_497_v7.0), *P. virgatum* (Pvirgatumvar_WBCHAP1_778_v1.0), *S. nutans* (Sn-CAM1369-DRAFT-PanAnd-1.0), and *T. dactyloides* (Td-FL_9056069_6-DRAFT-PanAnd-1.0); genome assemblies and annotations were obtained from MaizeGDB (PanAnd project) and the JGI Data Portal (see Data Availability section). Search databases contained 197,551 protein entries for *A. gerardi* (Ag-CAM1351-DRAFT-PanAnd-1.0; also used as the search database for *M. giganteus* samples in the same TMT batch, with peptides subsequently re-mapped to the *M. sinensis* proteome containing 67,789 entries), 95,264 entries for *P. virgatum* (Pvirgatumvar_WBCHAP1_778_v1.0), 123,723 entries for *S. nutans* (Sn-CAM1369-DRAFT-PanAnd-1.0), and 65,151 entries for *T. dactyloides* (Td-FL_9056069_6-DRAFT-PanAnd-1.0). All databases were supplemented with the cRAP common contaminants database for the Sequest HT search.

### Protein abundance normalization and statistical analysis

Protein intensities were normalized using the “total peptide amount” method in Proteome Discoverer 2.5. Differential abundance between winter and summer was assessed using one-way ANOVA followed by Tukey’s HSD post hoc test, with multiple testing correction by the Benjamini-Hochberg procedure. For the multi-batch *Tripsacum* 2022 dataset, statistical testing was performed within each batch and P-values were combined using Fisher’s method; only proteins quantified in at least 3 of 4 batches were retained. Proteins quantified in both seasons within each species were considered differentially abundant at |log_2_FC| ≥ 1 and adjusted *P* < 0.05.

PCA was performed on log_2_-transformed protein abundances (S1 Table) and on orthogroup-level data detected in >50% of samples per species (S2 Fig). For Tripsacum, batch effects were removed via residuals from a linear model prior to PCA; because season was the dominant factor (S2b Fig), all Tripsacum samples were pooled across years. For cross-species comparison, orthogroups were assigned using OrthoFinder (v2.5.5; [35], with fold-change values averaged when multiple proteins mapped to the same orthogroup. All analyses were performed in R v4.4.3.

### Cross-species correlation analysis

To assess evolutionary constraint on cold-responsive proteins, we compared cross-species conservation of seasonal response magnitude between shared cold-responsive orthogroups and background proteins, following the framework of Cope et al. [29].

For each orthogroup quantified in at least four of five species, we calculated pairwise Spearman correlations of log_2_ fold-change (winter/summer) across all species pairs. Orthogroups were classified as “shared DAPs” if differentially abundant (|log_2_FC| ≥ 1, adjusted *P* < 0.05) in two or more species, or as “background” if differentially abundant in at most one species. Distributions of pairwise correlations were compared between shared DAPs and background using the Wilcoxon rank-sum test. The same approach was applied to basal protein abundance (summer and winter separately) to distinguish constraint on response dynamics from constraint on absolute abundance levels.

To control for potential confounds in the correlation contrast, we applied two complementary permutation approaches (10,000 replicates each). First, to assess effect-size selection bias, shared and background labels were shuffled within |log_2_FC| magnitude bins, preserving the fold-change distribution of each group. Second, to control for shared ancestry, we used a Brownian-motion permulation approach: for each orthogroup independently, a BM rate was estimated from the observed fold-change vector and the species phylogeny, new tip values were simulated under this model, and the original observed values were reassigned by the ranks of the simulated values [36]. This preserves both the marginal distribution of each orthogroup and the phylogenetic covariance structure while breaking the coordinated cross-orthogroup signal. The test statistic was the median Δρ between shared DAPs and background across all 10 species pairs. Both tests were also run against magnitude-matched single-species DAPs (|log_2_FC| ≥ 0.53; n = 155) as an alternative comparison group.

### Functional annotation and GO enrichment

Orthogroups were assigned using OrthoFinder (v2.5.5; [35]), with DIAMOND (v2.1.9; [37]) for all-vs-all sequence comparisons across all five species. Orthogroups served as the basis for cross-species comparisons and functional propagation of annotations. DAP sequences were annotated using DIAMOND BLASTp against the SwissProt with gene ontology (GO) terms extracted from UniProt. For DAPs lacking SwissProt hits, functional predictions were generated using PANNZER2 [38], run independently for each species. Annotation of non-significant proteins is detailed in S1 Methods.

GO term enrichment analysis was performed using the topGO R package (v2.50.0; [39]), with the “elim” algorithm with Fisher’s exact test, separately for winter-elevated and winter-reduced proteins against all quantified proteins. Terms with a minimum node size of 10 and a P-value < 0.01 were considered significant. Due to the hierarchical nature of topGO’s algorithm, multiple test correction was not necessary [39].

### Candidate protein exploration methods

Barnes et al. [18] showed that cold-responsive transcriptional and proteomic changes often overlap with broader stress mechanisms involving damage, osmotic imbalance, and acidification. To improve biological resolution, we curated DAPs into nine functional categories based on known roles in freezing tolerance [8,40,41]: (1) protein aggregation and membrane stability, (2) protein folding and chaperone activity, (3) metabolism and osmoregulation, (4) detoxification and ROS scavenging, (5) lipid metabolism, (6) antifreeze and cell wall modification, (7) cold signal transduction, (8) cytoskeletal organization, and (9) other/uncategorized. Assignments were informed by protein descriptions, GO terms, PANNZER2 predictions, and domain feature review (S3 Fig).

### Selecting proteins for traits on trees

Proteins for phylogenetic trait mapping were identified by text-mining PANNZER2 annotations for GO terms associated with LEA proteins, HSPs, and EF1. Full-length sequences were extracted from reference proteomes using seqkit grep (v2.0), aligned with MAFFT (v7.475), and used to infer maximum-likelihood trees with raxmlHPC (RAxML-8.2.13, PROTGAMMAJTT, 100 bootstraps). Trees were rooted on appropriate outgroups and visualized with ggtree, mapping log_2_FC values as tip-point colors. Alignment and tree-building parameters are detailed in S1 Methods.

### RNA-seq processing and differential expression analysis

Transcriptome data for maize chilling responses across tissues (leaf, root, top-crown, and bottom-crown) were derived from Xue et al. [42] using 3′ RNA-seq (5C vs. 22C). For *Tripsacum*, we analyzed leaf transcriptome data from a segregating freezing-tolerant bulk of F_2_ seedlings derived from the same *Tripsacum* hybrids used in our rhizome analysis. Leaf tissue was collected from individual six-week-old F_2_ seedlings from seven F_2_ families at control temperature (22 °C) and again after seven days of cold acclimation (5 °C), prior to freezing exposure. The same seedlings were then screened by exposure to −8 °C for three hours, phenotyped six days post-freezing, and assigned retrospectively to freezing-tolerant or freezing-intolerant bulks following the criteria described previously **[**32**].** Only samples from seedlings assigned to the tolerant bulk were analysed here, with expression compared between the two temperatures (tol_5C vs. tol_22C). The second transcriptome from *Tripsacum* ‘Pete’ (leaf, root, top-crown, and bottom-crown) was generated using the same methodology (5°C vs. 22°C) as in Xue et al. [42] and is currently unpublished.

F_2_ seedling leaf RNA was extracted following Gault et al. [43]. Paired-end reads were quality-trimmed using Trimmomatic (v0.32; [44]), aligned to the T. dactyloides genome (Td-FL_9056069_6-DRAFT-PanAnd-1.0) using STAR v2.7.10b, and quantified with featureCounts v2.0.3. Differential expression was assessed using DESeq2 [45], modeling temperature and genotype as main effects. Genes were considered differentially expressed at adjusted P < 0.05 (Benjamini-Hochberg) and |log_2_FC| > 1. Trimming parameters, STAR alignment settings, and the full DESeq2 model are detailed in S1 Methods.

### Structural and biophysical analysis of LEA3 proteins

Because LEA3 emerged as the only differentially abundant protein consistently elevated across all five species (see Results) we performed targeted structural and biophysical analyses to characterize its repeat architecture and cryoprotective potential. LEA3 orthologs from all five study species plus *Zea mays* were aligned using MAFFT v7.520 with default settings.

Predicted three-dimensional structures for the *Tripsacum dactyloides* (Td00001aa022594_T002) and *Zea mays* (Zm00001eb294480) orthologs were generated using AlphaFold3 [46] with default parameters and full length amino acid sequence as input. Hydrophobicity was visualized in PyMOL (v2.5) using a custom script based on the Eisenberg scale, which colored residues according to side-chain properties. Hydropathy plots were generated using both ProtScale (ExPASy) and AmphipaSeek [47], with a sliding window size of 11 amino acids corresponding to the LEA3 motif length. Hydrophobic peaks associated with conserved LEA motifs were annotated across both sequence alignments and structural models.

## Results

To examine seasonal proteomic responses in PACMAD grasses, we analyzed rhizome tissues of five species using a proteomic shotgun approach during the winter (January) and summer (August) of 2019–2020 and 2022 (Fig 1) in Ithaca, New York (USDA Hardiness Zone 6a). The exposure to deep-freezing winters ensured that the proteomic responses captured were reflective of natural seasonal variation (Fig 1b).

Protein abundance analysis was performed in batches (see Methods). Across all five species a total of 20,737 proteins were quantified, but for differential accumulation across seasons we included only those quantified in both winter and summer samples (Table 1, S4 Fig). The number of identified proteins ranged from 2,372–4,451 per species. Differentially abundant proteins (DAPs) varied across species, with 58–174 proteins significantly increased and 56–218 decreased in abundance under freezing conditions (Table 1). PCA of 745 shared orthogroups confirmed clustering by species and season (S2 Fig); batch-corrected *Tripsacum* samples (1,889 proteins) clustered by season regardless of hybrid background (S2b Fig). Although fewer proteins were identified in *Sorghastrum nutans*, reflecting its draft genome status (S2 Table), robust seasonal signatures were detectable across all five lineages (S2a Fig).

### Cross-Species comparative proteomic analysis reveals conserved seasonal protein accumulation patterns

We restricted our comparative analysis to the 18,454 proteins assigned to 4,888 orthogroups identified across species (S3 and S4 Tables). Seasonal fold-changes were positively correlated across all species pairs (*ρ* = 0.37–0.69; Fig 2a), with specific orthogroups—including LEA3, GST, HSP1, and PEBP—consistently identified as differentially abundant. The full pattern of pairwise, triple, quadruple, and five-way DAP intersections across species is shown in S6 Fig. To test whether this conservation reflects evolutionary constraint on cold response, we compared fold-change correlations between shared DAPs (differential abundance in ≥2 species; n = 126 orthogroups) and background proteins (≤1 species; n = 1,557). Shared DAPs showed substantially higher correlation in seasonal response magnitude (*ρ* = 0.80) than background (*ρ* = 0.45; Fig 2b). This difference persisted after we controlled for effect-size selection bias (magnitude-matched permutation, *P* < 10^-4^) and for shared ancestry (Brownian motion permulation, *P* = 8 × 10^-4^; S5 Fig). Notably, this constraint did not extend to basal abundance, where shared DAPs showed slightly lower correlation than background (*ρ* = 0.62 vs 0.68; *P* = 0.004). This pattern of tight cross-species correlation in response dynamics combined with flexibility in baseline levels is consistent with conserved regulation of a core set of protective proteins. This result was robust when we excluded *S. nutans*, which had fewer biological replicates (n = 2 per season; *P* = 0.002).

**Fig 2:**
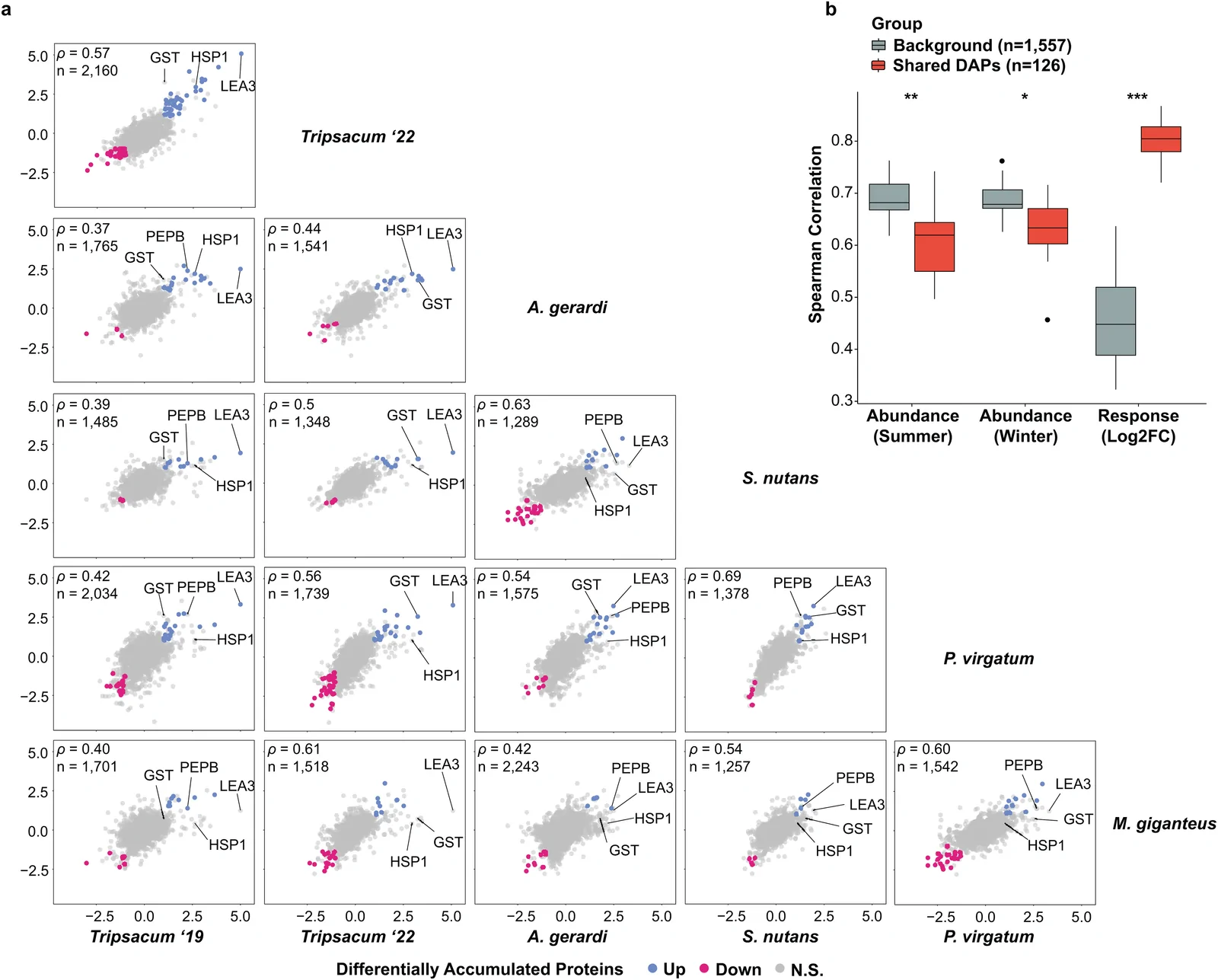
Conservation of seasonal protein accumulation in rhizomes of *Tripsacum dactyloides* and *T. floridanum* hybrids, *Andropogon gerardi*, *Miscanthus* × *giganteus*, *Panicum virgatum*, and *Sorghastrum nutans.* **(a)** Pairwise comparisons of log_2_ fold-change (log_2_FC winter/summer) in differentially accumulated orthogroups across species. Scatterplots display the correlation of orthogroup-level log_2_FC values between species, with shared DAPs elevated in winter shown in blue, reduced in red, and non-significant proteins in gray. Each panel represents a comparison between two species, with the number of shared orthogroups (*n*) and Spearman’s correlation coefficient (ρ) displayed. **(b)** Pairwise Spearman correlations for orthogroups quantified in at least four of five species, comparing shared DAPs (red; differentially abundant in ≥2 species) versus background orthogroups (gray; differentially abundant in ≤1 species). Correlations are shown for summer abundance, winter abundance, and seasonal response magnitude (log_2_FC winter/summer). Wilcoxon rank-sum test: *P < 0.05, **P < 0.01, ***P < 0.001.

### Ortholog and pathway sharing across species

We next examined orthogroup sharing across species (S6 Fig). Winter-elevated orthogroups showed greater conservation than winter-reduced: 27% of 330 winter-elevated orthogroups were shared across at least two species versus 21% of 470 winter-reduced orthogroups. Variation in identified proteins partly reflects reference proteome completeness—*S. nutans*, with a draft genome containing 26% ambiguous bases, yielded fewer identifications (S2 Table).

GO enrichment analysis revealed distinct functional signatures (S7a Fig). Winter-elevated proteins were enriched for protein folding, stress response, cold acclimation, and ROS scavenging (S5 Table). Winter-reduced proteins were enriched for microtubule-based processes, mitotic cell cycle, cytoskeleton organization, and cell wall biogenesis—processes associated with active growth (S6 Table). The greater conservation of winter-elevated orthogroups suggests that protective responses to freezing are more constrained than growth-related processes active during summer. To identify the most conserved functional themes, we examined the 50 most winter-elevated proteins per species (Fig 3; S7 Table). LEAs and heat shock proteins (HSPs) were present in at least four of five species. A chi-square test on standard GO categories revealed significant species heterogeneity (χ^2^ (70) = 97.5, *P* = 0.017), but GO terms may conflate cold-specific processes with broader stress responses [18]. 63% of proteins mapped to one of nine functional groups curated for relevance to cold (S7b Fig). Heterogeneity remained significant (χ^2^ (40) = 58.1, *P* = 0.032; Fig 3a), indicating that while species share the same core functional categories, they differ in relative investment. Detoxification and ROS scavenging proteins were represented in all five species. Metabolism/osmoregulation proteins were less prominent in *A. gerardi*, while protein aggregation and membrane stability proteins were enriched in *A. gerardi* and *P. virgatum*. These patterns indicate a shared functional core with lineage-specific variation in relative allocation (Fig 3a), particularly in metabolism/osmoregulation and protein folding/stability.

**Fig 3:**
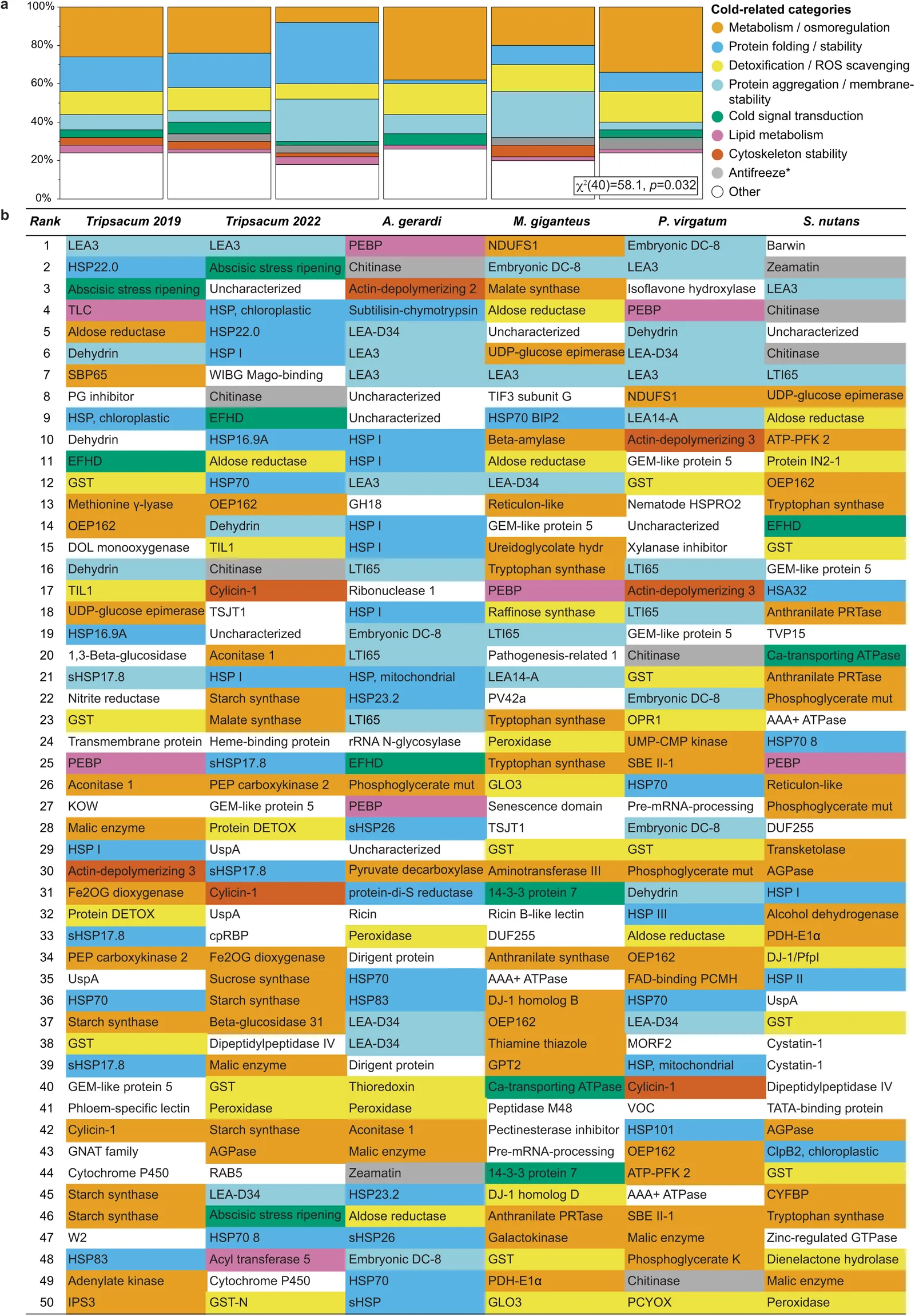
Overview of cold-related functional categories represented among the top 50 most winter-enriched rhizome proteins *Tripsacum dactyloides* and *T. floridanum* hybrids, *Andropogon gerardi*, *Miscanthus* × *giganteus*, *Panicum virgatum*, and *Sorghastrum nutans.* **(a)** Mosaic plot of cold-related functional categories across species. This plot visualizes the distribution of proteins among cold-related functional categories for each species. The relative abundance of each category within a species is shown, highlighting distinct functional profiles and species-specific variations in cold tolerance mechanisms. A chi-square test for independence (χ^2^ (40) = 58.1, *P* = 0.032) was performed to evaluate variation across species. **(b)** Visual representation of cold-related functional categories in rhizome proteins of five PACMAD species. This figure illustrates the top 50 most abundant proteins within the rhizomes of five *Andropogoneae* species. Proteins are classified into functional categories relevant to cold-related mechanisms, such as metabolism/osmoregulation, cold signal transduction, membrane stability, lipid metabolism, and others. Color-coding facilitates a comparative visualization of cold tolerance mechanisms across species. Antifreeze proteins are marked by an asterisk, as their classification is based solely on protein homology.

LEA3 (OG0022470) was the only ortholog ranked among the top 10 winter-elevated proteins in all five species and was the single most elevated protein in *Tripsacum* (log_2_FC = 5.0; Fig 3b; S7 Table). Other functional categories showed species-specific representation: HSPs appeared in the top 10 for *Tripsacum* (HSP22, Class IV) and *S. nutans* (HSP23.2) but involved different family members; ROS scavenging was universal but employed glutathione transferases in four species and peroxidases in *A. gerardi*; putative antifreeze proteins (chitinases) ranked highly in *S. nutans* and *A. gerardi*. This pattern—a single conserved ortholog (LEA3) alongside functionally equivalent but distinct proteins in other categories—exemplifies parallel recruitment of LEA3 alongside functional convergence for chaperone and antioxidant functions.

### Evolutionary patterns in accumulated protein families

Phylogenetic analysis revealed distinct evolutionary patterns between LEAs and HSP families (Fig 4). The control elongation factor 1 protein (EF1) family, previously shown to have stable expression at transcript and protein levels across conditions ([48], Fig 4a), showed minimal seasonal accumulation and no phylogenetic clustering of abundance level patterns, consistent with their housekeeping function (S8 Table). HSPs showed greater diversity (212 proteins across 37 orthogroups, S9 Table) compared to LEAs (66 proteins in 20 orthogroups, S10 Table), with control EF1 comprising 33 proteins in 4 orthogroups. Both LEA and HSP phylogeny (Fig 4b and 4c) resolved into clear functional sub-families, but among HSPs the most strongly winter-elevated members (log_2_FC > 2.5) belonged to different clades across different species, with only 32% of HSP proteins significantly elevated in winter. In contrast, LEA proteins (Fig 4b) demonstrated more consistent winter abundance (47% of the proteins), particularly within the LEA3 subfamily, which displayed elevated abundance across all species despite having diverged earlier than other LEA clades.

**Fig 4:**
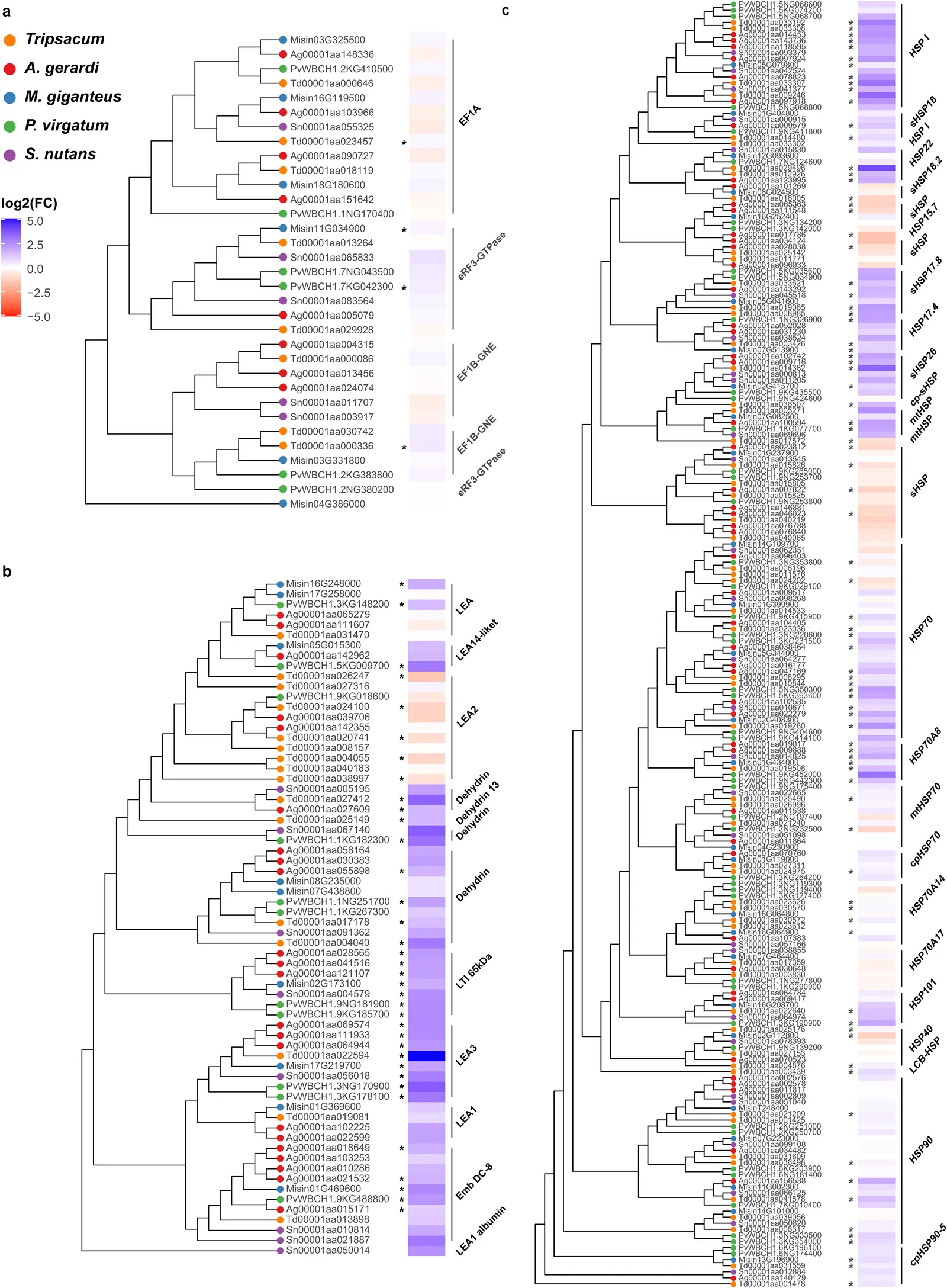
Phylogenetic analysis and seasonal abundance dynamics of selected protein families across *Tripsacum dactyloides* and *T. floridanum* hybrids, *Panicum virgatum*, *Andropogon gerardi*, *Miscanthus* × *giganteus*, and *Sorghastrum nutans.* Protein identifiers represent species-specific sequences, and brackets denote functional group annotations, merging adjacent orthogroups with similar functions for clarity. Heat maps illustrate log_2_ fold-change (log_2_FC) in protein abundance between winter and summer conditions, where blue indicates higher abundance in winter, and red signifies higher summer abundance. Panels show distinct protein families: **(a)** translation elongation factor 1 proteins (EF1; control group), **(b)** late embryogenesis abundant (LEA) proteins, and **(c)** heat shock proteins (HSPs). Asterisks (*) mark proteins exhibiting statistically significant differential accumulation (*P* < 0.05).

### Beyond rhizomes: Comparison with maize & Tripsacum mRNA

We next tested whether rhizome protein signatures corresponded to mRNA responses in other tissues. Cold-induced mRNA in F_2_ seedling leaves of *Tripsacum*, derived from the same hybrids used in our rhizome study, showed weak but significant correlation with rhizome protein fold-changes (*ρ* = 0.29, *P* < 0.001; S8a Fig). Other *Tripsacum* tissues showed weaker correlations (*ρ* = 0.08–0.16; S8b–S8e Fig), as did maize orthologs (*ρ* = 0.01–0.09; S8f–S8i Fig). Despite these weak overall correlations, LEA3 displayed strong cold induction in leaves of both species (log_2_FC = 8 in *Tripsacum*, and 7 in maize; S8a and S8f Fig). That maize induces LEA3 transcription to levels comparable to cold-tolerant *Tripsacum*, yet remains freezing-sensitive, suggests that transcriptional induction of *LEA3* alone does not confer protection.

### Structural and functional insights into LEA3: A key player in freezing tolerance

LEA3 (OG0022470) was the only ortholog consistently elevated across all five species, ranking as the top DAP in *Tripsacum* rhizomes (log_2_FC = 5.0) and the most cold-induced transcript in leaves (log_2_FC = 8). Sequence alignment revealed that all six PACMAD orthologs share the Group 3 LEA architecture (S9 Fig), featuring tandem 11-mer repeats that fold into amphipathic α-helices (Fig 5b). LEA3 proteins are classified as intrinsically disordered proteins thought to adopt amphipathic alpha-helical conformations upon binding to membranes or other proteins during cellular dehydration [49]. These repeats generate characteristic oscillations in Kyte-Doolittle hydropathy profiles (Fig 5c; S11 Table; S10 Fig), consistent with a hydrophobic face (positions 1, 2, 5, 9) and a charged hydrophilic face essential for desiccation and freezing tolerance [50]. While the number of repeats varied—ranging from three (*M. giganteus*, *Z. mays*) to five (*T. dactyloides*, *A. gerardi*)—total LEA3 dosage was further modulated by gene copy number, with *A. gerardi* and *P. virgatum* accumulating multiple paralogs (Fig 4b; S10 Table). Notably, although *M. giganteus* and *Z. mays* share the same three-repeat architecture, their biophysical properties diverge. The *M. giganteus* ortholog retains hydrophilic consensus repeats, while the maize ortholog contains a variant third repeat (AADAMEAAKQK) with alanine and methionine substitutions that increase local hydrophobicity (repeat GRAVY −0.67 vs consensus mean −1.41; S12 Table). This variation produces a distinct hydrophobic peak in the hydropathy profile (Fig 5c) and a more pronounced hydrophobic face in the predicted structure (Fig 5b), potentially compromising the balance between hydrophobic and hydrophilic faces required for effective cryoprotection. Hydrophobicity scores for individual LEA3 orthologs are provided in S11 Table.

**Fig 5:**
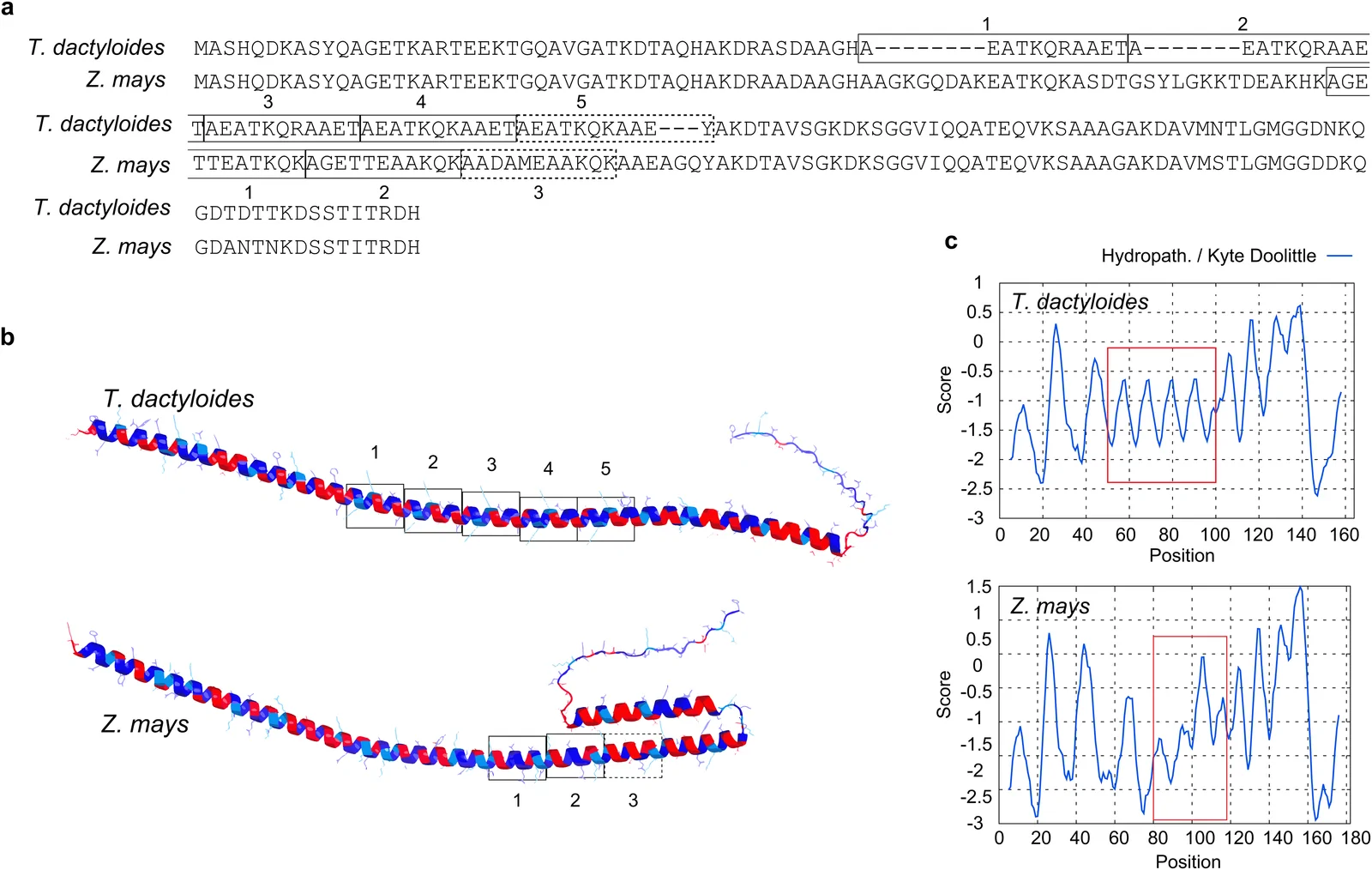
Protein sequence alignment, structure and hydrophobicity of LEA3 orthologs from *Tripsacum dactyloides* and *Zea mays.* **(a)** The alignment shows the LEA3 proteins from *T. dactyloides* (Td00001aa022594_T002) and, *Z. mays* (Zm00001eb294480), with tandem 11-mer repeat units highlighted and labeled numerically. *T. dactyloides* contains five repeat units (1–5); *Z. mays* contains three (1–3). The dashed box indicates a repeat unit with variant residues relative to the consensus repeat sequence. **(b)** Predicted secondary structure of LEA3 proteins using AlphaFold3, showing the conserved alpha-helical domains across *T. dactyloides* (top) and *Z. mays* (bottom). Repeat regions corresponding to labeled sequence motifs are boxed. Red and blue indicate regions of high hydrophilicity and hydrophobicity, respectively. **(c)** Kyte-Doolittle hydropathy plots for LEA3 proteins. The X-axis represents residue position, while the Y-axis indicates hydropathicity scores (positive values for hydrophobic regions, negative for hydrophilic regions). Red boxes highlight the amphipathic region encompassing the 11-mer tandem repeat array, corresponding to key domains in (b).

Hydropathy profiles from all cold-tolerant species examined show oscillating patterns consistent with amphipathic helix periodicity (S10 Fig). Species with more repeats exhibit more oscillation cycles and consequently longer amphipathic regions. Outgroup LEA3 proteins from wheat (TdLEA3; [51]) and *Arabidopsis* (AtLEA7; [52]) showed comparable repeat architecture. These observations suggest that transcriptional induction of *LEA3* alone may not ensure freezing tolerance and that functional protection may depend on both protein accumulation in the appropriate tissue and the biophysical properties of the tandem repeats of the 11-mer motif.

## Discussion

Our analysis of rhizome proteomes from five independently cold-adapted PACMAD grasses reveals that protein-level cold responses combine ancestral retention of core protective orthologs with convergent deployment of functionally equivalent but phylogenetically distinct proteins. The clearest example of retention is LEA3, accumulated across all five lineages: its early divergence from other LEA subfamilies (Fig 4b) and the conservation of its tandem 11-mer repeat architecture across cold-tolerant species suggest that the cryoprotective capacity of LEA3 predates the PACMAD radiation and has been maintained under constraint in lineages that colonized cold environments (Fig 1e). In contrast, the chaperone response illustrates convergent evolution: different HSP family members are recruited in different species, suggesting that chaperone-mediated protection permits greater flexibility in which family members are deployed. To our knowledge, this study provides the first quantitative comparison of cross-species fold-change conservation at the protein level in a freezing-stress context, complementing transcript-level comparisons in *Pooideae* [53] and desiccation-tolerance studies in resurrection grasses [54].

The most striking finding is evidence for evolutionary constraint on response magnitude, meaning a higher cross-species correlation in cold-induced fold-change among shared cold-responsive orthogroups than among proteins responsive in at most one species. Among shared cold-responsive orthogroups, cross-species fold-change correlations were substantially higher (*ρ* = 0.80, Fig 2b) than among background proteins (*ρ* = 0.45), a difference that persisted after controlling for effect-size selection bias and shared ancestry (S5 Fig). This indicates that when freezing tolerance evolved independently across PACMAD lineages, it proceeded through quantitatively similar responses in a core set of protective proteins. Our common-garden design ensured identical winter conditions across all genotypes, with winter collections following sustained sub-zero exposure reaching −16°C and soil temperatures below 2–5°C for 20–22 days [31], while summer collections captured vigorous growth with actively emerging rhizome buds after >90 mm cumulative precipitation (S1 Fig), establishing these seasonal contrasts as cold-driven rather than drought-related. A limitation of this design is that all species experienced identical winter conditions rather than stress calibrated to each species’ tolerance threshold. A second limitation is the reduced replication of *S. nutans* (n = 2 after winter mortality), which yields wider per-species confidence intervals than for the other species. The headline cross-species correlation contrast is preserved when *S. nutans* is excluded (*P* = 0.002; reported above), and orthogroup-level fold-change summarization buffers, but does not eliminate, replication-driven variance. The *Tripsacum* genus is represented as a single collapsed entry in cross-species tests to avoid pseudo-replication of one lineage.

The cross-species correlations we observe are consistent with constraint on response magnitude but do not uniquely imply a single mechanism. At least three non-mutually exclusive explanations could generate the patterns reported here. First, shared physiological limits: independently of evolutionary history, the biophysical demands of freezing tolerance impose a narrow set of viable molecular responses, and any organism meeting those demands would converge on similar solutions. Second, environmental uniformity: the common-garden experiment exposes all species to identical thermal cues, and shared cues can elicit correlated responses from independently evolved sensing pathways without invoking deep evolutionary constraint. Third, statistical-threshold artifacts: the |log_2_FC| ≥ 1 selection criterion preferentially retains proteins with strong, reproducible responses, biasing toward shared signal independently of any underlying biology. Our magnitude-matched permutation control (effect-size selection bias, *P* < 10 ^-4^) addresses the third explanation, and the Brownian-motion permulation (*P* = 8 × 10^-4^; S5 Fig) controls for shared ancestry, but neither rules out shared physiological limits or environmental uniformity. With these responses established as cold-driven, their conservation contrasts sharply with transcript-level patterns: comparative transcriptomics in *Pooideae* found generally low similarity in cold-responsive gene expression across species (*r* = 0.09–0.40 [53]), with nearly half of all cold-responsive genes being species-specific. This comparison spans different clades, tissues, and experimental designs (our PACMAD rhizome proteomes versus *Pooideae* seedling transcriptomes), so the gap between protein-level and transcript-level correlations may be confounded by these differences. Nevertheless, the direction is consistent with the framework of Cope et al. [29], who demonstrated that protein abundances are under stronger stabilizing selection than mRNA abundances over macroevolutionary timescales, with mutations affecting mRNA levels largely buffered before they alter protein output. If this buffering operates on cold-responsive genes as it does genome-wide, then transcript-level comparisons would systematically underestimate the degree of evolutionary conservation in stress responses. Notably, this constraint applies specifically to the dynamic response: absolute abundance correlations for shared cold-responsive proteins were lower than background in both summer (*ρ* = 0.62 vs 0.68; *P* = 0.004) and winter (Fig 2b), indicating that these proteins are more divergent across species in their resting and stress-state levels. What is conserved is not the absolute abundance in either season, but the magnitude of accumulation when freezing arrives. This divergence in absolute levels likely reflects the distinct physiological contexts of each species’ rhizomes, including differences in dormancy depth, metabolic rate, or tissue composition, while the conserved fold-change suggests that selection acts primarily on the dynamic response rather than on any single optimal protein concentration. Such buffering is plausibly under particularly strong selection in an overwintering rhizome, which must maintain a functional, dormancy-competent proteome over months rather than minutes.

A central feature of our experimental design is that the tissue analyzed is subterranean. Soil thermally buffers below-ground organs against the diurnal extremes that aerial tissues experience, and the dormant rhizome inhabits a relatively stable, sustained sub-zero environment rather than the acute, fluctuating cold stress imposed on leaves. The conservation patterns reported here should therefore be read as specific to a dormancy organ; they do not necessarily generalize to leaf-level cold-shock responses or to other tissues that experience more variable thermal regimes.

LEA3 provides the clearest illustration of this constraint and connects the evolutionary pattern to a mechanistic hypothesis. LEA proteins function as molecular shields during freezing through membrane stabilization, the prevention of protein aggregation, and the protection of client proteins through steric exclusion and stress induced conformational changes [49,55]. These functions depend on the repeating 11-mer motif architecture, described by Dure [50], in which apolar residues form the hydrophobic face of α-helices that bind membranes and partially unfolded proteins during dehydration and freezing [51,56].

The structural data support a model in which cryoprotective efficacy arises from three factors: amphipathic helix length per protein (determined by repeat number), total protein abundance (increased through paralog accumulation in *A. gerardi* and *P. virgatum*), and repeat composition. All cold-tolerant species and outgroups maintain conserved amphipathic character across their 11-mer repeats (S9 Fig, S10 Fig; S12 Table), whereas the variant third repeat of Z. mays shows an exaggerated hydrophobic face that may compromise the amphipathic balance required for effective cryoprotection (S12 Table). AlphaFold3 multimer predictions reinforce this distinction: while both Td and ZmLEA3 form tetrameric assemblies, they adopt distinct packing geometries (S11 Fig), consistent with observations that cereal LEA3 proteins require conserved domains for oligomerization [57] and Arabidopsis LEA3 proteins form higher-order assemblies under stress [58,59]. The variant hydrophobic face in ZmLEA3 may disrupt the optimal packing arrangement required for effective cryoprotection. This structural divergence helps explain why, although ZmLEA3 is cold-inducible and confers partial cold tolerance [60], maize remains freezing-sensitive despite accumulating transcripts to levels comparable to the cold-tolerant *Tripsacum* in leaves (S8f Fig). Transcriptional induction alone does not ensure protection; functional efficacy depends on both sufficient abundance and activity, the latter shaped by the biophysical properties of the repeat region. The early divergence of LEA3 from other LEA subfamilies (Fig 4b) suggests that the molecular properties enabling cryoprotection were likely present before the PACMAD radiation. Cold-tolerant lineages maintained or expanded LEA3 repeat number and in some cases accumulated multiple paralogs, while in maize, where freezing selection was relaxed, repeat number was not expanded and the third repeat diverged from the hydrophilic consensus. Maize encodes a single LEA3 ortholog, with no copy-number expansion to offset the shorter, more hydrophobic protein architecture.

Direct experimental validation of the proposed LEA3 contribution, for example through heterologous expression, repeat-number perturbation, or *in vitro* cryoprotection assays, is required to establish causation and is beyond the scope of the present study. Even with such validation, freezing survival involves the coordinated action of numerous protective mechanisms, and restoring a single protein’s architecture in a freezing-sensitive species would be unlikely to confer full tolerance. Rather, LEA3 illustrates how structural constraints at the protein level could limit the efficacy of otherwise conserved transcriptional responses.

More broadly, several of the most consistently accumulated proteins across PACMAD rhizomes have well-characterized orthologs implicated in cold acclimation in *Arabidopsis*, including LEA proteins, small HSPs, and glutathione S-transferases [61–63], indicating deep conservation of core protective pathways across monocots and dicots. However, some highly accumulated proteins in PACMAD rhizomes—notably PEBP family members and certain chitinase-like proteins—have not been prominently featured in *Arabidopsis* cold studies, potentially reflecting grass-specific or rhizome-specific adaptations [64].

The presence of ABA-inducible proteins and dehydrins among the most accumulated winter proteins across species is consistent with shared physiological pathways between drought and frost responses. While evidence shows that drought tolerance evolved before frost tolerance in PACMAD grasses, suggesting they may have been co-opted, the extent to which this actually happened remains unclear [65]. In contrast, Pooideae grasses show a different trajectory, where frost tolerance appears to have evolved independently of, or even prior to, drought tolerance [65,66].

Orthogroup-level analysis also identifies a smaller set of conservation outliers: orthogroups whose response in a single species substantially exceeds the cross-species median, despite belonging to functional families otherwise represented in two or more species (S13 Table). Notable examples include a Temperature-Induced Lipocalin (TIL1) orthogroup elevated specifically in *Tripsacum* (log_2_FC = 2.7) while remaining near baseline in the other four species, a chitinase-like GH18 protein elevated in *A. gerardi* (log_2_FC = 2.3), and ureidoglycolate hydrolase elevated only in *M. giganteus* (log_2_FC = 1.9). These species-specific responders, drawn from otherwise conserved cold-related categories, may reflect lineage-specific adaptations to local environmental conditions or divergence in regulatory networks acting on conserved protein families. We highlight these outliers as biologically meaningful exceptions rather than as a counter-thesis to the conserved-magnitude pattern; the headline correlation result is not contingent on their presence.

In conclusion, rhizome freezing tolerance across independently cold-adapted PACMAD grasses is consistent with retention of ancestral protective proteins under quantitative constraint on response magnitude, combined with convergent deployment of diverse protein families for chaperone and antioxidant functions. This constraint is substantially stronger at the protein level than previously observed at the transcript level. The differences in LEA3 repeat number and composition between cold-tolerant PACMAD species and maize offer a candidate mechanism for enhancing freezing resilience in maize. Future work should prioritize functional validation of LEA3 repeat variants, particularly testing whether restoring the ancestral hydrophilic consensus or introducing additional paralogs can enhance frost tolerance in maize.

## Supporting information

S1 Fig Rhizome morphology at sampling. (PDF)

S2 Fig Principal component analysis of log-transformed protein abundance data. (PDF)

S3 Fig Biological processes (BPs) within curated cold-related functional categories. (PDF)

S4 Fig Volcano plots showing differentially accumulated proteins in sampled species. (PDF)

S5 Fig Permutation and permulation controls for the cross-species fold-change correlation contrast. (PDF)

S6 Fig Upset plots of shared differentially accumulated proteins among species in winter and summer. (PDF)

S7 Fig Enriched BPs in rhizomes of five PACMAD grass species and BP distribution within curated cold-related functional categories. (PDF)

S8 Fig Scatterplots showing the relationship between rhizome proteins and cold-responsive mRNA expression in *Tripsacum* and maize tissues. (PDF)

S9 Fig MAFFT alignment of LEA3 across six PACMAD species. (PDF)

S10 Fig Hydropathy profiles of LEA3 orthologs. (PDF)

S11 Fig AlphaFold 3 prediction of LEA3 homo-tetramer assemblies in *Tripsacum* and *Zea mays.* (PDF)

S1 Table Normalized protein abundances (log_2_-transformed) for all samples. (ZIP)

S2 Table Genome assembly statistics for the five species analyzed. (XLSX)

S3 Table List of 4,888 orthogroups identified across species. (ZIP)

S4 Table Log_2_ fold-changes (Winter/Summer) for all quantified proteins. (XLSX)

S5 Table GO enrichment analysis for winter-upregulated proteins. (XLSX)

S6 Table GO enrichment analysis for winter-downregulated proteins. (XLSX)

S7 Table Top 50 most abundant proteins in winter vs summer. (XLSX)

S8 Table Elongation Factor 1-alpha (EF1) orthologs and abundance data. (XLSX)

S9 Table Heat Shock Protein (HSP) orthologs and abundance data. (XLSX)

S10 Table LEA3 orthologs and abundance data. (XLSX)

S11 Table Hydrophobicity scores for LEA3 orthologs. (XLSX)

S12 Table Biophysical properties of individual 11mer LEA3 (OG0022470) tandem repeats. (XLSX)

S13 Table Conservation outliers: orthogroups in conserved categories significant in only one species. (XLSX)

S14 Table PRIDE sample key and metadata. (XLSX)

S1 Methods Detailed protocols for protein extraction, S-Trap digestion, LC and mass spectrometric acquisition, instrument quality control, database search parameters, RNA-seq alignment settings, and bioinformatics pipeline parameters. (DOCX)
