# Supplementary material for: Evolutionary constraint on a core winter proteome across independently cold-adapted PACMAD grasses
Source: PLoS One. 2026 Oct 14;21(10):e0358458. doi: 10.1371/journal.pone.0358458 (PMC13667808; doi:10.1371/journal.pone.0358458)
Supplement: Supplementary file 1 [file pone.0358458.s001.pdf]

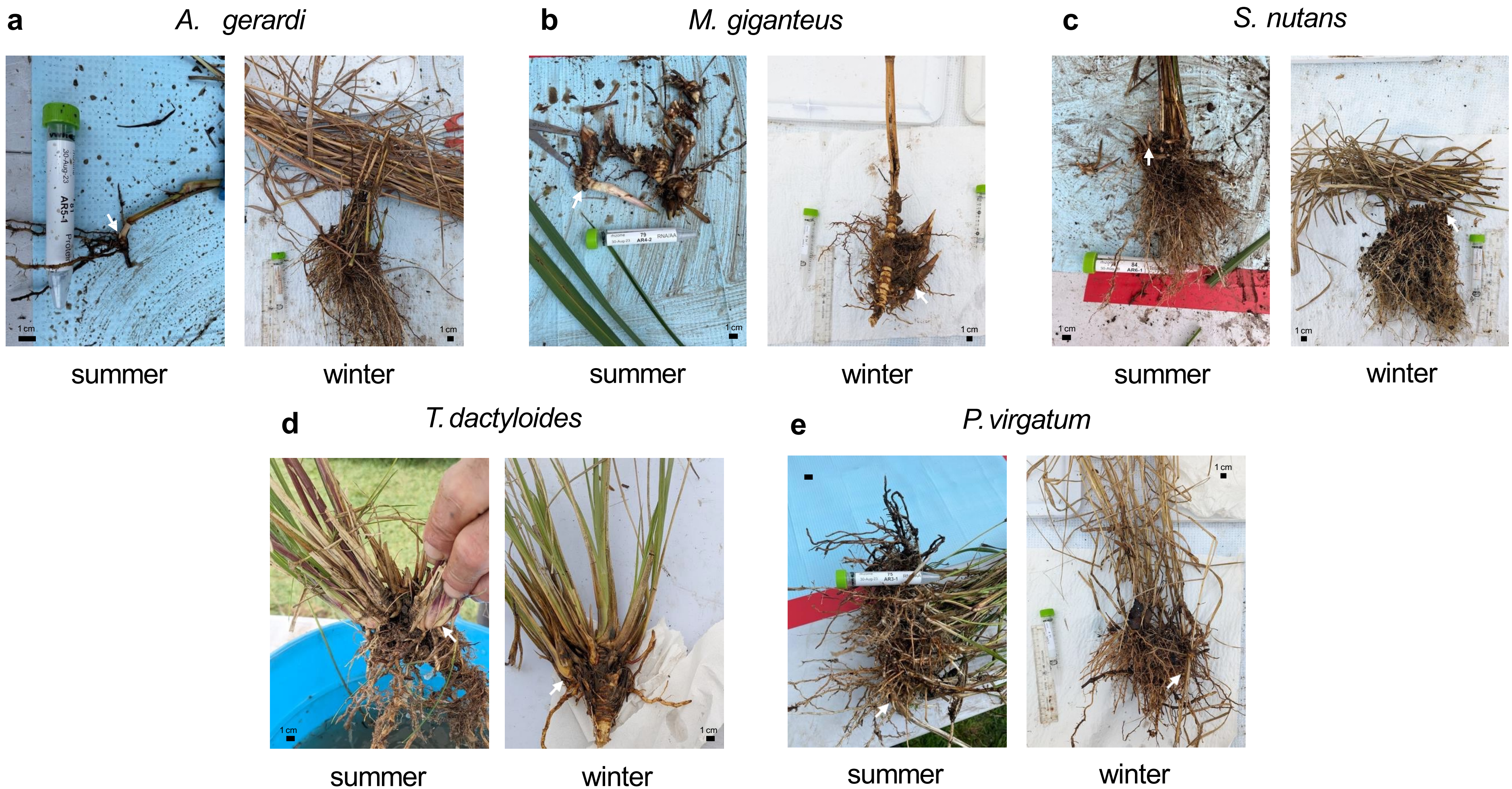

S1 Fig. Rhizome morphology at sampling. Paired images display representative rhizome tissues (white arrows) collected during summer (August, left) and winter (January, right) for **(a)** *Andropogon gerardi*, **(b)** *Miscanthus* × *giganteus*, **(c)** *Sorghastrum nutans*, **(d)** *Tripsacum dactyloides* and *T. floridanum* hybrids, and **(e)** *Panicum virgatum*. Summer samples exhibit active bud growth and pale storage tissue. Winter samples exhibit dormancy and epidermal hardening. Proteomic analysis was restricted to mid-section of these storage organs. Scale bars represent approx. 1 cm.
