# Supplementary material for: Evolutionary constraint on a core winter proteome across independently cold-adapted PACMAD grasses
Source: PLoS One. 2026 Oct 14;21(10):e0358458. doi: 10.1371/journal.pone.0358458 (PMC13667808; doi:10.1371/journal.pone.0358458)
Supplement: Supplementary file 2 [file pone.0358458.s002.pdf]

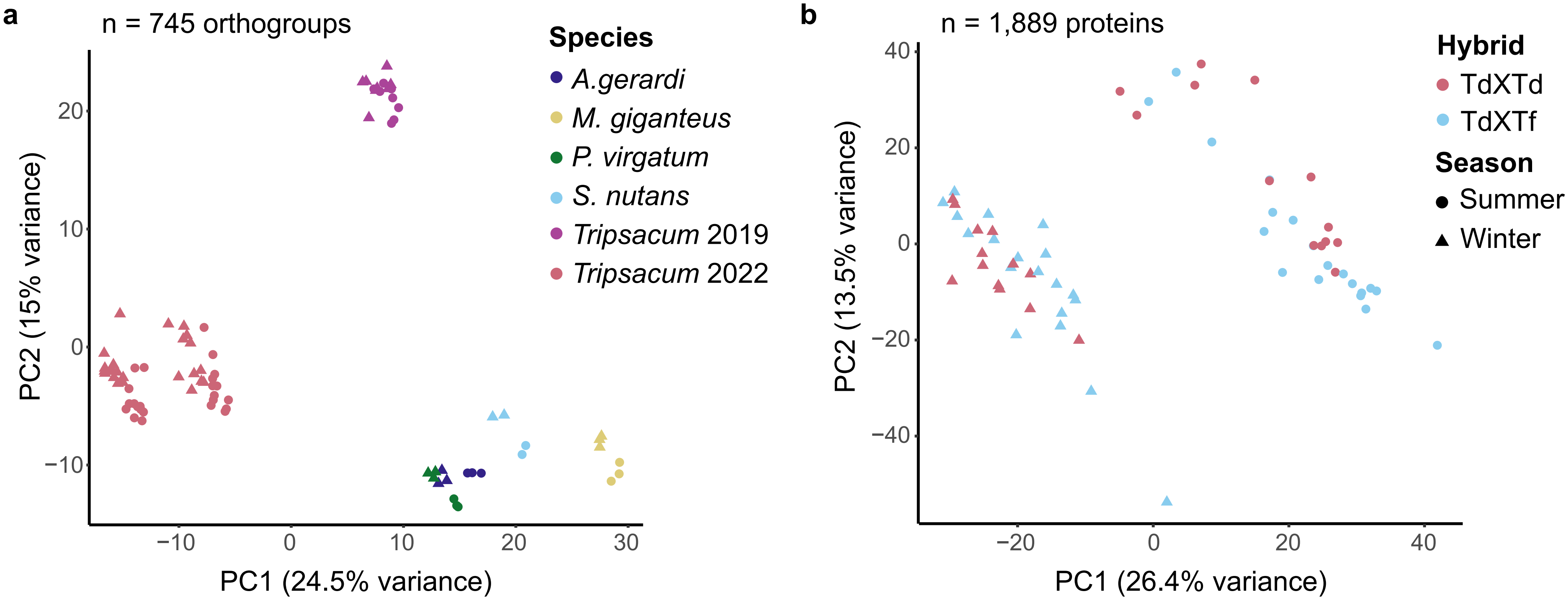

S2 Fig. Principal component analysis (PCA) of log-transformed protein abundance data. (a) PCA of 745 orthogroups from five species *Tripsacum dactyloides and T. floridanum hybrids* (Td-2019, Td-2022), *Andropogon gerardi* (Ag), *Miscanthus × giganteus* (Mg), *Panicum virgatum* (Pv), and *Sorghastrum nutans* (Sn). **(b)** PCA of 1,889 proteins from *Tripsacum* hybrids. Points are colored by hybrid type–intraspecific *T. dactyloides* hybrids (Td × Td) and interspecific *T. dactyloides* × *T. floridanum* hybrids (Td × Tf). Shapes represent season (circle = summer, triangle = winter). The percentage of total variance explained by each principal component is shown on the axes.
