# Supplementary material for: Evolutionary constraint on a core winter proteome across independently cold-adapted PACMAD grasses
Source: PLoS One. 2026 Oct 14;21(10):e0358458. doi: 10.1371/journal.pone.0358458 (PMC13667808; doi:10.1371/journal.pone.0358458)
Supplement: Supplementary file 3 [file pone.0358458.s003.pdf]

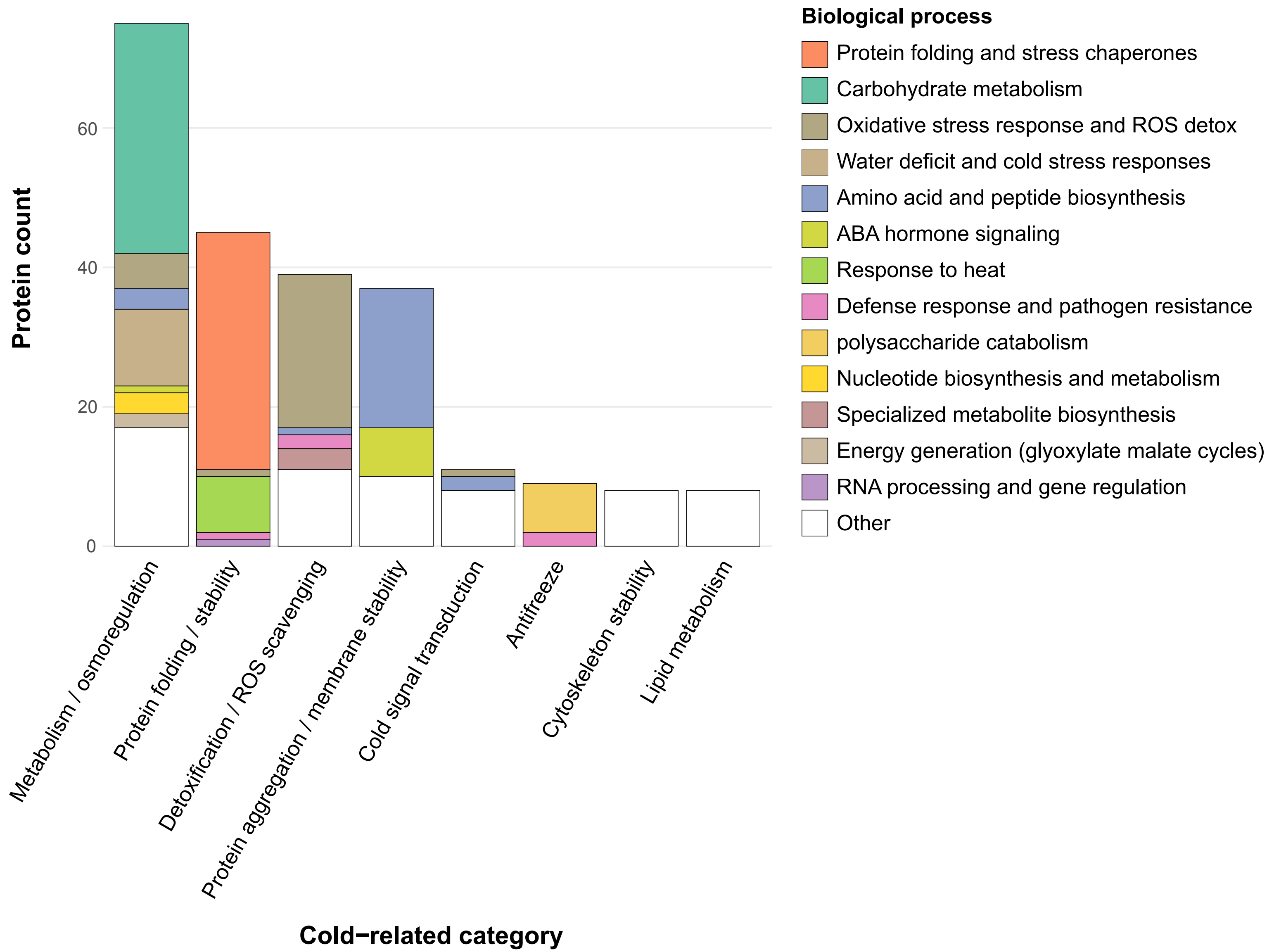

S3 Fig. Stacked bar chart illustrating the composition of biological processes (BPs) within curated cold-related functional categories. The plot includes 230 proteins from the top 50 differentially accumulated proteins (DAPs) accumulated in winter rhizomes of *Tripsacum dactyloides* and *T. floridanum* hybrids, *Andropogon gerardi*, *Miscanthus × giganteus*, *Panicum virgatum*, and *Sorghastrum nutans*, after excluding those assigned to the cold response category "Other" (i.e., proteins with annotations deemed less relevant to cold tolerance interpretation). Cold-related categories are shown on the x-axis and sorted by total protein count. Each bar is color-coded by BP, highlighting the functional composition of each category. Less informative or poorly annotated BPs were grouped under "Other" in the legend. This visualization supports the manual consolidation of specific biological processes into broader cold-response categories used throughout the study.
