# Supplementary material for: Evolutionary constraint on a core winter proteome across independently cold-adapted PACMAD grasses
Source: PLoS One. 2026 Oct 14;21(10):e0358458. doi: 10.1371/journal.pone.0358458 (PMC13667808; doi:10.1371/journal.pone.0358458)
Supplement: Supplementary file 4 [file pone.0358458.s004.pdf]

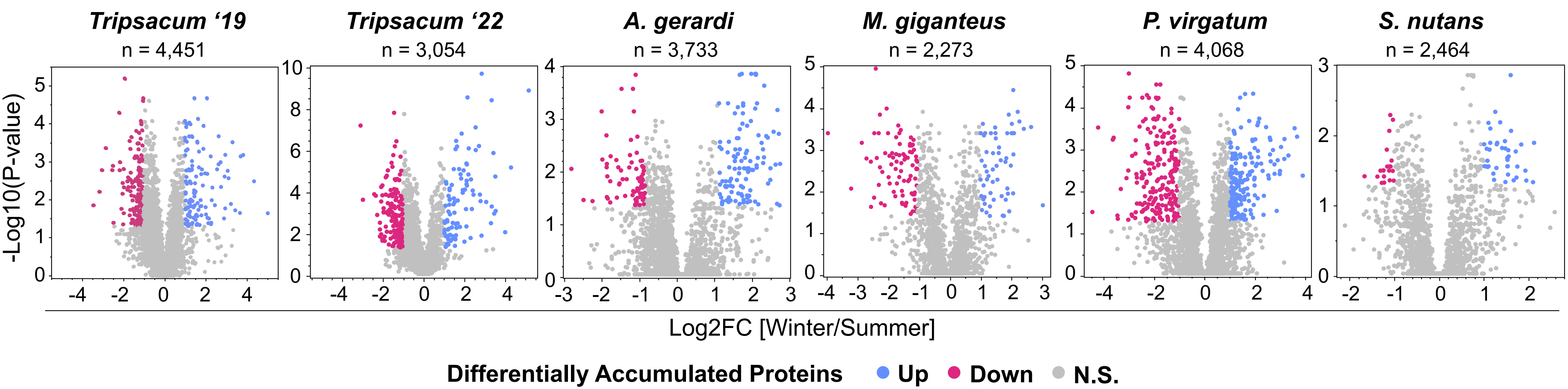

S4 Fig. Volcano plots showing differentially accumulated proteins (DAPs) in rhizomes of *Tripsacum dactyloides and T. floridanum hybrids, Andropogon gerardi, Miscanthus* × *giganteus*, *Panicum virgatum*, and *Sorghastrum nutans* under winter vs. summer conditions, with total number of proteins identified listed. Proteins with significantly elevated (blue) and reduced (pink) abundance in winter relative to summer are highlighted, with non-significant proteins in gray. The y-axis represents the significance as $-\log_{10}$(p-value), and the x-axis shows the $\log_2$ fold-change ($\log_2$FC) of the DAPs between winter and summer.
