# Supplementary material for: Evolutionary constraint on a core winter proteome across independently cold-adapted PACMAD grasses
Source: PLoS One. 2026 Oct 14;21(10):e0358458. doi: 10.1371/journal.pone.0358458 (PMC13667808; doi:10.1371/journal.pone.0358458)
Supplement: Supplementary file 5 [file pone.0358458.s005.pdf]

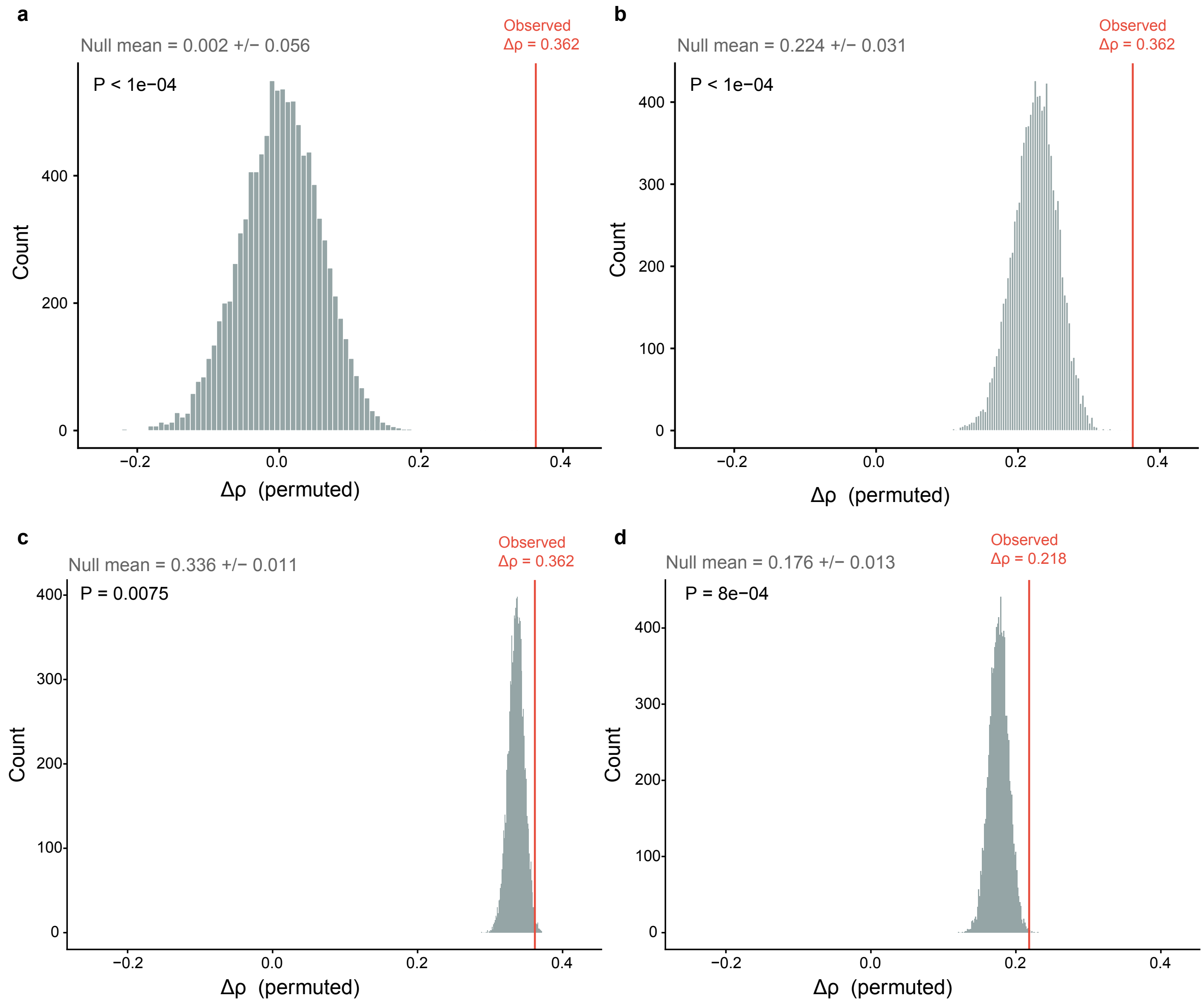

S5 Fig. Permutation and permulation controls for the cross-species fold-change correlation contrast. The observed $\Delta\rho$ (median Spearman ρ of shared DAPs minus background) is shown as a red line in each panel. (**a**) Naive permutation: shared/background labels shuffled randomly (10,000 replicates). (**b**) Magnitude-matched permutation: labels shuffled within $|\log_2 FC|$ bins. (**c**) Brownian-motion (BM) permulation against all background (n = 1,557): for each orthogroup independently, fold-change values were reassigned across species using rank-matched BM simulation along the species phylogeny (10,000 replicates). (**d**) BM permulation against magnitude-matched single-species DAPs (n = 155; $|\log_2 FC| \geq 0.53$). The observed $\Delta\rho$ in panel (d) is 0.218 because the comparison group is magnitude-matched single-species DAPs rather than all background.
