# Supplementary material for: Evolutionary constraint on a core winter proteome across independently cold-adapted PACMAD grasses
Source: PLoS One. 2026 Oct 14;21(10):e0358458. doi: 10.1371/journal.pone.0358458 (PMC13667808; doi:10.1371/journal.pone.0358458)
Supplement: Supplementary file 6 [file pone.0358458.s006.pdf]

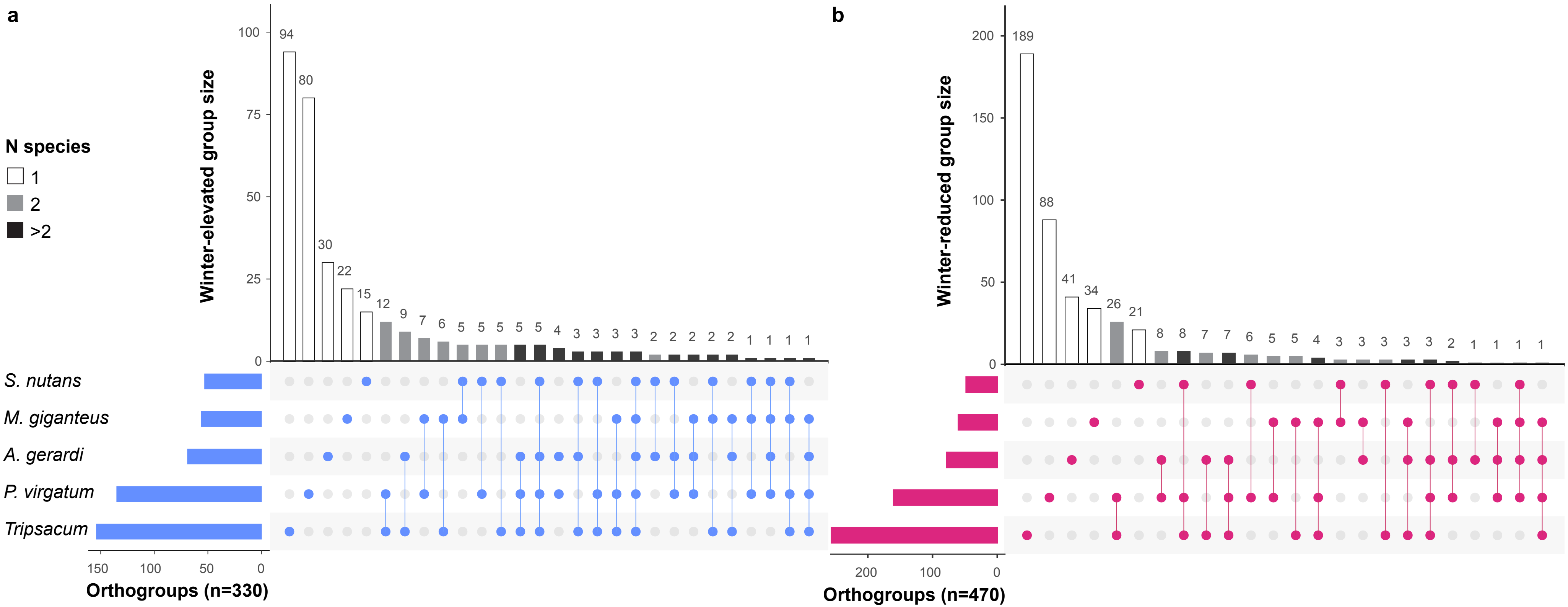

S6 Fig. Orthogroup sharing of significantly DAPs among *Tripsacum dactyloides and T. floridanum hybrids, Andropogon gerardi, Miscanthus* × *giganteus*, *Panicum virgatum*, and *Sorghastrum nutans*. (a) Winter-elevated. **(b)** Winter-reduced. Set sizes (horizontal bars) indicate the total number of differentially abundant orthogroups identified per species. Intersection sizes (vertical bars) represent orthogroups unique to individual species or shared among multiple species. Bar shading indicates the number of species sharing each orthogroup: unique to one species (white), shared between two species (gray), or shared among more than two species (black). Dots below the plot illustrate species-specific orthogroup intersections.
