# Supplementary material for: Evolutionary constraint on a core winter proteome across independently cold-adapted PACMAD grasses
Source: PLoS One. 2026 Oct 14;21(10):e0358458. doi: 10.1371/journal.pone.0358458 (PMC13667808; doi:10.1371/journal.pone.0358458)
Supplement: Supplementary file 7 [file pone.0358458.s007.pdf]

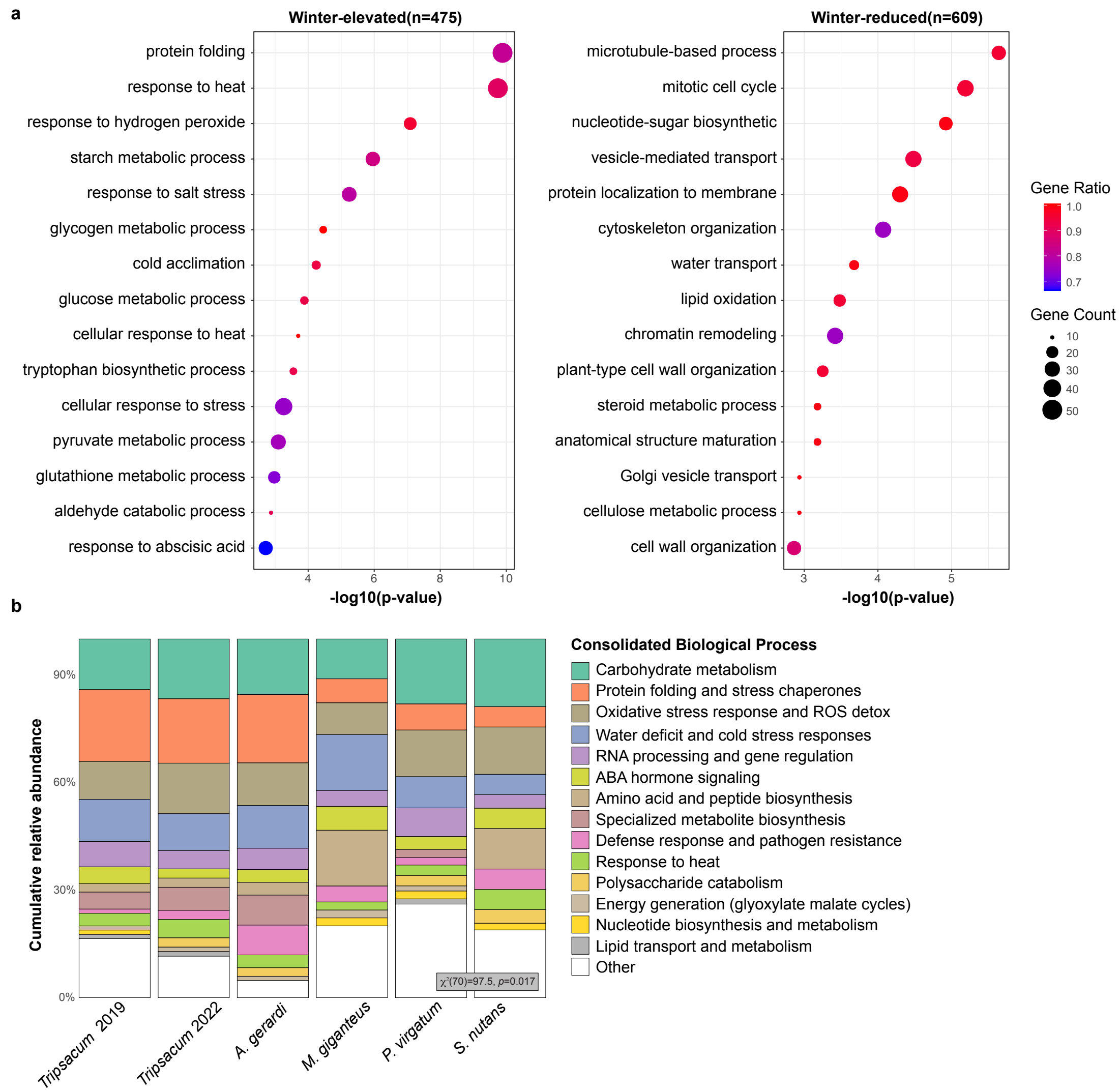

S7 Fig. (a) Enriched biological processes (BP) of *Tripsacum dactyloides* and *T. floridanum* hybrids, *Andropogon gerardi*, *Miscanthus × giganteus*, *Panicum virgatum*, and *Sorghastrum nutans* rhizomes among differentially accumulated proteins (DAPs) in 475 winter-elevated and 609 winter-reduced proteins, rather than the orthogroup counts (n=330 and n=470) discussed in the main text, to capture functional diversity among paralogs. Proteins with unknown BP term were excluded). Dot size represents gene count per BP category, while dot color indicates the gene ratio (proportion of proteins annotated to each BP category). Only the top significantly enriched BP terms (lowest *P*-values) are displayed for clarity. **(b)** Mosaic plot of BP enriched among winter-elevated DAPs across five PACMAD grass species. The plot summarizes the distribution of functionally annotated proteins (n = 483 of 630; unannotated proteins were excluded) across 139 biological process (BP) terms. These terms were consolidated into broader functional categories to aid interpretability. Each bar shows the relative abundance of functional categories within a species, highlighting both conserved and species-specific patterns. A Chi-square test for independence ($\chi^2[70]=97.5$, $P=0.017$) was performed to evaluate variation across species. Category labels are shown in the legend.
