# Supplementary material for: Evolutionary constraint on a core winter proteome across independently cold-adapted PACMAD grasses
Source: PLoS One. 2026 Oct 14;21(10):e0358458. doi: 10.1371/journal.pone.0358458 (PMC13667808; doi:10.1371/journal.pone.0358458)
Supplement: Supplementary file 8 [file pone.0358458.s008.pdf]

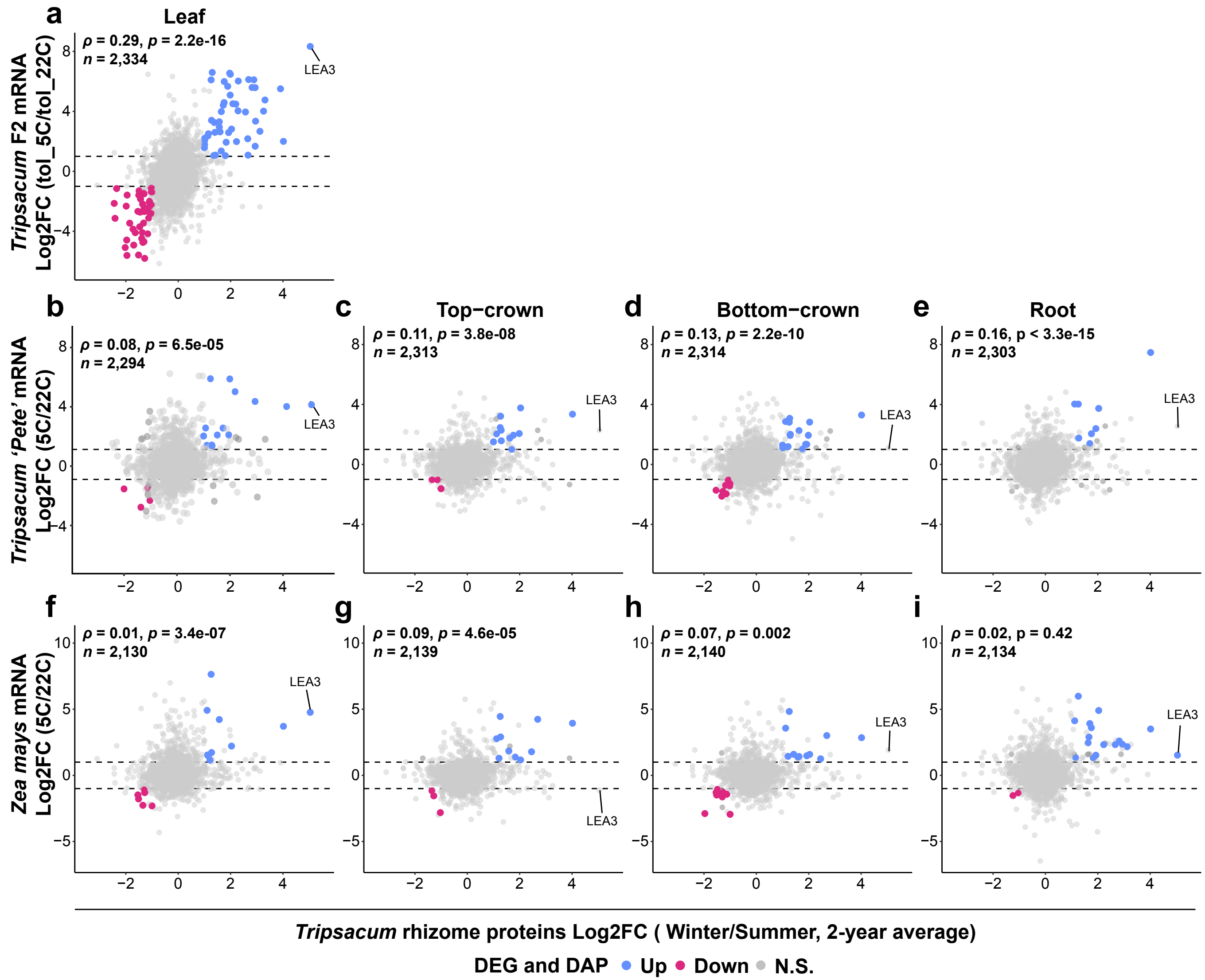

S8 Fig. Relationship between seasonal protein abundance in rhizomes of *Tripsacum dactyloides* and *T. floridanum* hybrids and cold-responsive RNA expression in *Tripsacum* hybrids, *Tripsacum dactyloides*, and *Zea mays*. **(a)** Scatterplots showing the relationship between *Tripsacum* rhizome proteins (x-axis, log2FC Winter/Summer, 2-year average) and *Tripsacum* leaf mRNA from a freezing tolerant F_2 bulk before and after 7 days cold acclimation (tol_5°C/tol_22°C) descended from the same *Tripsacum* hybrids used for rhizome proteomic analysis. **(b–e)** *Tripsacum dactyloides* (cv. 'Pete') tissues (5°C/22°C): leaf, top-crown, bottom-crown, and root. **(f–i)** *Zea mays* tissues (5°C/22°C): leaf, top-crown, bottom-crown, and root. Points are colored by shared differential expression status: blue for upregulated, pink for downregulated, and gray for non-significant changes. Dashed horizontal lines indicate the ±1 log2FC thresholds. Spearman's correlation coefficient (ρ) and *P*-value are shown in the upper left of each panel, along with the number of genes analyzed (*n*). The LEA3 gene is labeled in each panel.
