# Supplementary material for: Evolutionary constraint on a core winter proteome across independently cold-adapted PACMAD grasses
Source: PLoS One. 2026 Oct 14;21(10):e0358458. doi: 10.1371/journal.pone.0358458 (PMC13667808; doi:10.1371/journal.pone.0358458)
Supplement: Supplementary file 9 [file pone.0358458.s009.pdf]

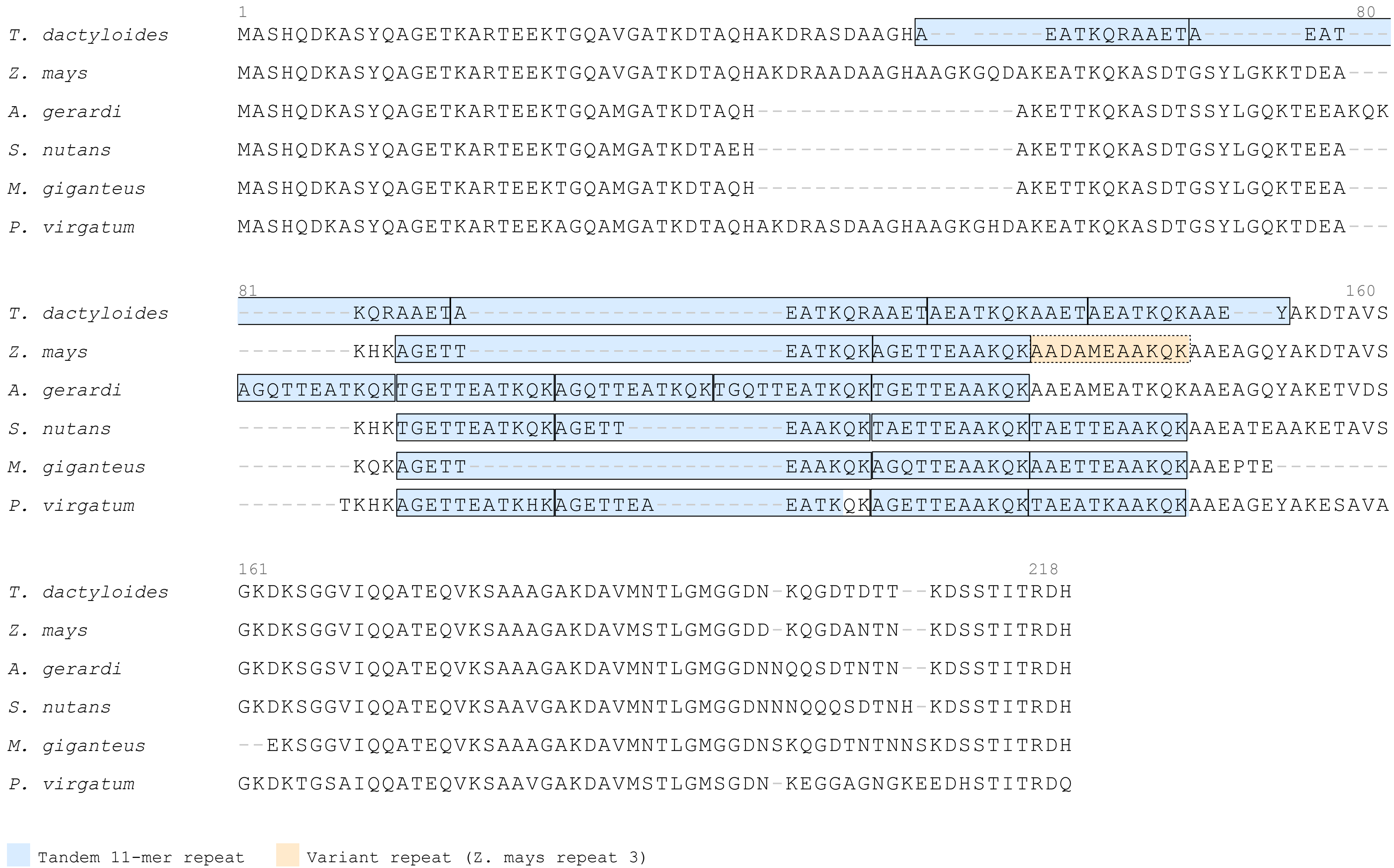

S9 Fig. Multiple sequence alignment of LEA3 orthologs across six PACMAD species. MAFFT alignment of LEA3 (OG0022470) protein sequences from *Tripsacum dactyloides* (Td00001aa022594), *Zea mays* (Zm00001eb294480), *Andropogon gerardi* (Ag00001aa069574), *Sorghastrum nutans* (Sn00001aa056018), *Miscanthus giganteus* (Misin17G219700), and *Panicum virgatum* (PvWBCH1.3NG170900). Blue shading indicates tandem 11-mer repeat units; orange shading indicates the variant third repeat of *Z. mays*.
