# Supplementary material for: Evolutionary constraint on a core winter proteome across independently cold-adapted PACMAD grasses
Source: PLoS One. 2026 Oct 14;21(10):e0358458. doi: 10.1371/journal.pone.0358458 (PMC13667808; doi:10.1371/journal.pone.0358458)
Supplement: Supplementary file 10 [file pone.0358458.s010.pdf]

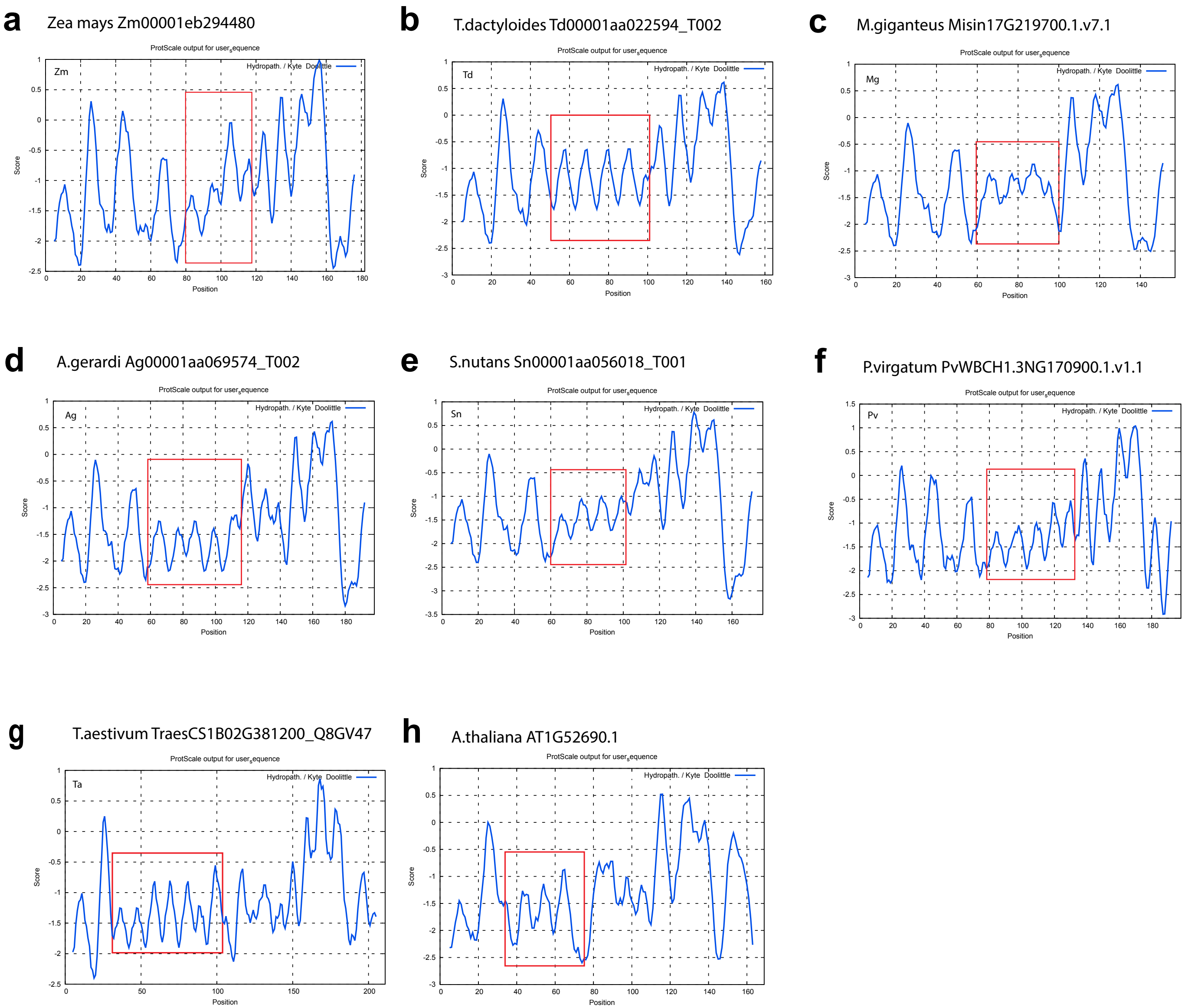

S10 Fig. Hydropathy profiles of LEA3 orthologs. **(a)** *Zea mays,* **(b)** *Tripsacum dactyloides*, **(c)** *Miscanthus × giganteus*, **(d)** *Andropogon gerardi*, **(e)** *Sorghastrum nutans*, **(f)** *Panicum virgatum*, **(g)** *Triticum aestivum*, and **(h)** *Arabidopsis thaliana*. Kyte-Doolittle plots were generated using ProtScale (ExPASy) with a window size of 11 amino acids. The x-axis indicates amino acid position, and the y-axis represents hydropathy score. Red boxes indicate the tandem 11-mer repeat region for each species.
