# Supplementary material for: Evolutionary constraint on a core winter proteome across independently cold-adapted PACMAD grasses
Source: PLoS One. 2026 Oct 14;21(10):e0358458. doi: 10.1371/journal.pone.0358458 (PMC13667808; doi:10.1371/journal.pone.0358458)
Supplement: Supplementary file 11 [file pone.0358458.s011.pdf]

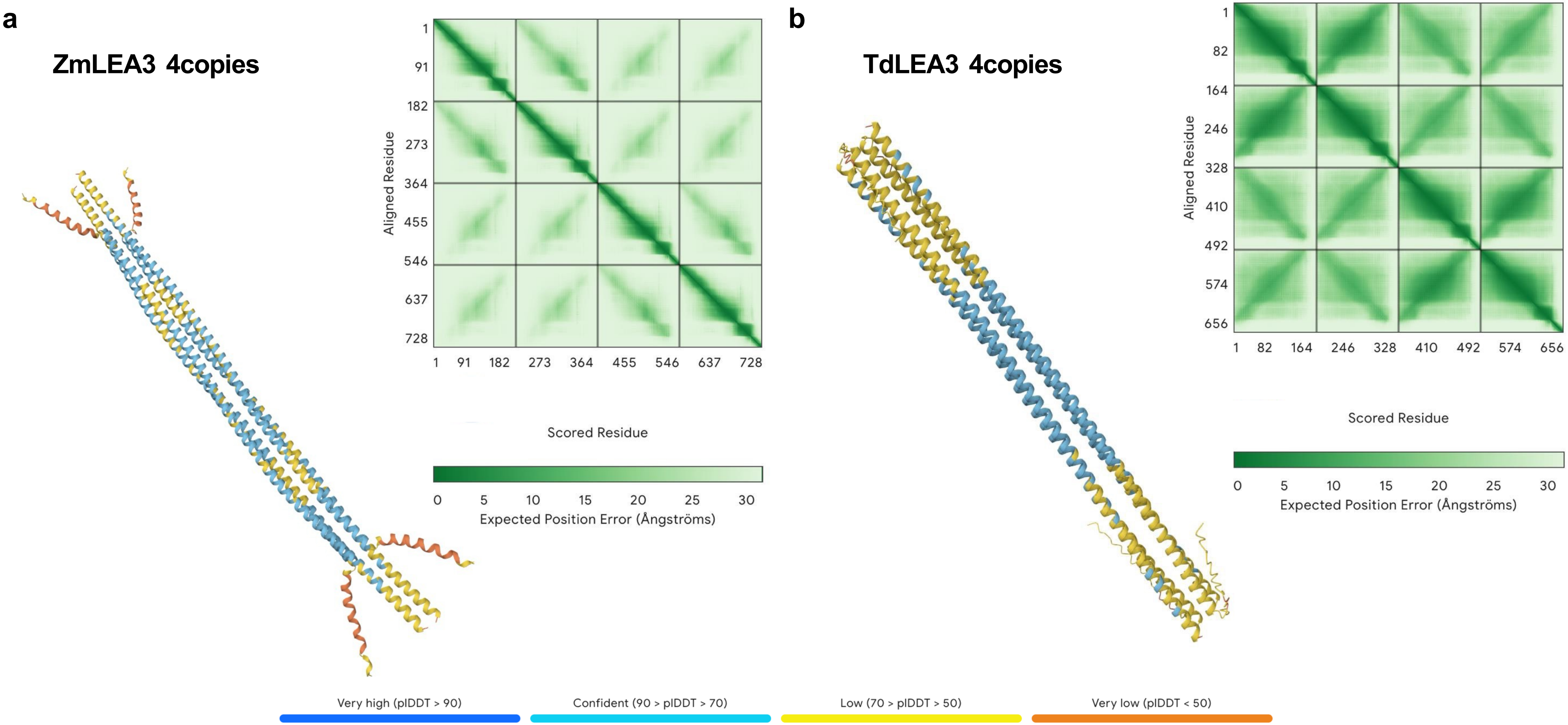

S11 Fig. AlphaFold 3 prediction of LEA3 homo-tetramer assemblies. Predicted quaternary structures of LEA3 homotetramers for **(a)** *Zea mays* and **(b)** *Tripsacum dactyloides*. Both orthologs are predicted to form tetrameric assemblies. Colors indicate per-residue confidence (pLDDT), with dark blue/blue representing high to very high confidence. The *Z. mays* complex displays a distinct oligomeric packing arrangement relative to *Tripsacum*. Predicted Aligned Error (PAE) plots. Low error values (dark green) in the off-diagonal regions confirm high confidence in the relative positioning of the subunits, validating the predicted tetrameric interfaces for both species.
