# Supplementary material for: Evolutionary constraint on a core winter proteome across independently cold-adapted PACMAD grasses
Source: PLoS One. 2026 Oct 14;21(10):e0358458. doi: 10.1371/journal.pone.0358458 (PMC13667808; doi:10.1371/journal.pone.0358458)
Supplement: Supplementary file 12 [file pone.0358458.s026.docx]

Supporting Information S1 Methods

Detailed environmental data, protocols for protein extraction, S-Trap digestion, LC and mass spectrometric acquisition, instrument quality control, database search parameters, proteins abundance statistical analyses, GO enrichment, RNA-seq alignment settings, and bioinformatics pipeline parameters for: Evolutionary constraint on a core winter proteome across independently cold-adapted PACMAD grasses by Oren et al*.*

### Sampling environmental data

In 2019, summer samples were collected on August 11 following 121 mm of precipitation in the preceding 30 days, and mean soil temperature at 5 cm depth was 19.9°C. Winter samples were collected on January 8, 2020, after 22 days with soil temperature below 2°C. In 2022, summer sampling occurred on August 15 following 93 mm of precipitation; 30-day mean soil temperature was 20.6°C. Winter sampling on December 9 followed 20 days with soil temperature below 5°C.

### Protein Extraction

Fresh rhizome tissue was ground in liquid nitrogen. The ground sample was suspended in a lysis buffer composed of 1× PBS (pH 8), 10 mM EDTA, 900 mM sucrose, and 0.4% β-mercaptoethanol. The suspension was vortexed and incubated on ice, then mixed with an equal volume of phenol buffered to pH 8 with Tris-EDTA (TE). After centrifugation and phase separation, the phenol phase containing proteins was collected. Proteins were precipitated from the phenol phase using ice-cold ammonium acetate/methanol solution (100 mM w/v ammonium acetate in 100% methanol) with protease and phosphatase inhibitors. After precipitation the pellets were washed five times with three different solutions and frozen.

### S-Trap Digestion

Protein pellets were dissolved in 8 M urea buffer. Protein concentration was determined using a BCA protein assay. A set amount of each protein sample underwent S-Trap digestion, including treatment with dithiothreitol (DTT) for reduction and iodoacetamide (IAA) for alkylation. The sample underwent multiple rounds of centrifugation with the addition of 8 M urea, followed by 50 mM ammonium bicarbonate (ABC) or triethylammonium bicarbonate (TEAB). The sample was digested using a trypsin buffer, centrifuged, and then dried by vacuum freeze-drying for storage prior to subsequent TMT labeling.

### High-pH Reversed-Phase Fractionation

Labeled peptides were fractionated by high-pH reversed-phase chromatography on a Dionex Ultimate 3000 HPLC system. A total of 48 fractions were collected and concatenated into 10 pools using a meandering pooling scheme designed to maximize orthogonality with subsequent low-pH nanoLC separation.

### Mass Spectrometric Acquisition

Labeled peptides were separated on an Ultimate 3000 nano-LC system (Thermo Fisher Scientific) coupled to an Orbitrap Eclipse Tribrid mass spectrometer (Thermo Fisher Scientific). Peptides were loaded onto a PepMap C-18 reversed-phase nano-trap column (5 µm particle size, 100 µm i.d. × 20 mm; Dionex) and separated on a PepMap C-18 reversed-phase analytical column (2 µm particle size, 75 µm i.d. × 25 cm). Mobile phase A was 2% acetonitrile in 0.1% aqueous formic acid; mobile phase B was 95% acetonitrile in 0.1% formic acid. Peptides were eluted at 300 nL/min with the column oven maintained at 35 °C, using a 120-min gradient from 5% to 33% B, followed by an 8-min ramp to 90% B, a 9-min hold at 90% B, and a 25-min re-equilibration at 2% B before the next injection. Mass spectra were acquired using a Real-Time Search synchronous precursor selection MS3 (RTS-SPS-MS3) method (Fu *et al.*, 2021). Precursor ions (MS1) were measured in the Orbitrap with an AGC target of 4 × 10⁵. MS2 fragmentation was performed by collision-induced dissociation (CID) in the linear ion trap with a normalized collision energy of 30%, isolation width 0.7 m/z, maximum injection time 50 ms, and AGC target 1 × 10⁴. MS3 fragmentation of the top 10 SPS precursors was performed by higher-energy collisional dissociation (HCD) in the Orbitrap with a normalized collision energy of 55%, isolation width 1.1 m/z, maximum injection time 120 ms, and AGC target 1 × 10⁵. The Real-Time Search node searched MS2 spectra against the corresponding species-specific reference proteome using TMTpro on peptide N-termini and lysine residues and carbamidomethyl on cysteine as static modifications, and oxidation on methionine as a variable modification, with a maximum of one missed cleavage and two variable modifications per peptide. No inclusion list was used.

### Instrument Quality Control and Calibration

Mass accuracy was monitored using the protonated hexakis (2,2-dimethoxypropane) cyclodisiloxane lock-mass ion at m/z 445.120029, with a tolerance of 5 ppm standard deviation maintained on the Orbitrap Eclipse prior to sample injection. When the mass error of the lock-mass ion exceeded 5 ppm, the instrument was recalibrated using the Pierce FlexMix calibration solution. Escherichia coli tryptic digest and bovine serum albumin (BSA) were analyzed as quality-assurance samples prior to each sample series. Instrument performance was evaluated using the total ion chromatogram and base peak chromatogram of the E. coli digest and the extracted ion chromatograms of representative BSA peptides.

### Database Search Parameters

Raw MS data were processed in Proteome Discoverer 2.5 using the Sequest HT search engine against species-specific reference proteomes (see main text, Peptide Mapping and Reference Proteome Assignment). Trypsin was specified as the proteolytic enzyme, allowing up to two missed cleavages with a minimum peptide length of six amino acids. Fixed modifications included TMTpro tags on peptide N-termini and lysine residues and carbamidomethylation of cysteine. Dynamic modifications included methionine oxidation, asparagine/glutamine deamidation, N-terminal acetylation, methionine loss, and methionine loss with acetylation. Precursor mass tolerance was set to 10 ppm and fragment mass tolerance to 0.6 Da.

### Peptide and Protein Validation

Peptide spectral matches were filtered using Percolator with a strict target FDR of 0.01 and a relaxed target FDR of 0.05. Protein identifications were validated using the Protein FDR Validator node with the same strict (0.01) and relaxed (0.05) FDR thresholds. Proteins were initially identified with a minimum of one unique peptide and subsequently filtered to retain only those supported by at least two distinct peptides for downstream analysis.

### Reporter Ion Quantification

Protein quantification was based on the summed reporter ion intensities of unique and razor peptides, filtered with a minimum signal-to-noise ratio of 10, SPS mass matches ≥65%, and a co-isolation threshold of 50%. No minimum channel occupancy threshold was applied, allowing quantification of proteins detected in subsets of samples. Total peptide amount normalization was applied across TMT channels. Protein abundance ratios were calculated based on summed reporter ion intensities, and hypothesis testing was performed using one-way ANOVA within Proteome Discoverer 2.5.

### Protein abundance normalization and statistical analysis

PCA was performed using the FactoMineR::PCA function in R with centering and scaling. For cross-species comparison at the orthogroup level, in cases where multiple proteins belonged to the same orthogroup within a species, log₂ fold-change values were averaged to generate a single orthogroup-level fold change. For differential abundance analysis, only proteins quantified in both winter and summer samples within each species were retained. All statistical analyses and visualization were performed in R version 4.4.3, using RStudio 2024.12.1+563 ("Kousa Dogwood" release).

### Functional annotation and GO enrichment

Orthogroups served as the basis for cross-species comparisons and functional propagation of annotations, particularly for proteins with limited annotation coverage outside *T. dactyloides.* For non-significant proteins, functional annotations were propagated from *Tripsacum dactyloides* to orthologous proteins within the same orthogroup when available. Remaining unannotated entries were manually curated based on sequence similarity and conserved domain features.

The topGO R package (v2.50.0; Alexa & Rahnenfuhrer, 2017), “elim” algorithm with Fisher’s exact test was used to reduce redundancy and account for GO hierarchy. The background set included all proteins quantified across the experiment. The top 15 enriched Biological Process (BP) terms in each group were visualized using dot plots in ggplot2, incorporating log-transformed P-values, gene ratios, and counts of significant proteins.

### Phylogenetic Analysis of Candidate Protein Families

Proteins for phylogenetic trait mapping were identified by text‐mining functional annotations produced by PANNZER2 for both the *Tripsacum dactyloides* proteome and the DAP sets from all five species, searching specifically for GO terms associated with LEA proteins, HSPs, and EF1. Extracted orthogroup identifiers from our log₂ fold‐change matrix were then used to retrieve the corresponding protein IDs, which served as input for sequence extraction. Full-length amino-acid sequences were obtained from each species’ reference proteome using seqkit grep (v2.0) with protein headers matching extracted IDs. Multiple sequence alignments were generated in MAFFT (v7.475; (Katoh & Standley, 2013) under default settings. Maximum-likelihood trees were inferred using raxmlHPC (RAxML-8.2.13; (Stamatakis, 2014) with the PROTGAMMAJTT substitution model and 100 rapid bootstraps. Each resulting tree was rooted on a biologically appropriate outgroup (e.g., a chloroplastic class I small HSP) and visualized in R (v4.1.0) using the ggtree package.

### RNA-seq Processing Parameters

Paired-end RNA-seq reads were quality-filtered and trimmed using Trimmomatic (v0.32; (Bolger *et al.*, 2014) to remove Illumina adapter bases, discarding leading bases with a phred score below 35 and trailing bases with a phred score below 20. Reads shorter than 36 bp after trimming were discarded.

Trimmed reads were aligned to the Tripsacum dactyloides genome (Td-FL_9056069_6-DRAFT-PanAnd-1.0.fasta) and annotation (Td-FL_9056069_6-DRAFT-PanAnd-1.0_final.gff3) using STAR v2.7.10b in two-pass mode (--twopassMode Basic), allowing each read to map to a maximum of 20 genomic locations (--outFilterMultimapNmax 20). Gene-level counts were extracted from uniquely mapped reads using featureCounts v2.0.3 with default settings.

Differential gene expression was assessed using DESeq2 (Love *et al.*, 2014), modeling both genotype and temperature as main effects (~ genotype + temperature). Contrasts were made between 5°C and 22°C within each of eight F₂ families, with 5°C as the reference level. Raw counts were normalized using the estimateSizeFactors method. Genes were considered differentially expressed at adjusted P < 0.05 (Benjamini-Hochberg correction) and |log₂FC| > 1.

References

Alexa A, Rahnenfuhrer J. 2017. *topGO: Enrichment analysis for gene ontology*. Bioconductor.

Bolger AM, Lohse M, Usadel B. 2014. Trimmomatic: a flexible trimmer for Illumina sequence data. *Bioinformatics* 30: 2114–2120.

Fu Q, Liu Z, Bhawal R, Anderson ET, Sherwood RW, Yang Y, Thannhauser T, Schroyen M, Tang X, Zhang H, *et al.* 2021. Comparison of MS2, synchronous precursor selection MS3, and real-time search MS3 methodologies for lung proteomes of hydrogen sulfide treated swine. *Anal. Bioanal. Chem.* 413: 419–429.

Katoh K, Standley DM. 2013. MAFFT multiple sequence alignment software version 7: improvements in performance and usability. *Mol. Biol. Evol.* 30: 772–780.

Love MI, Huber W, Anders S. 2014. Moderated estimation of fold change and dispersion for RNA-seq data with DESeq2. *Genome Biol.* 15: 550.

Stamatakis A. 2014. RAxML version 8: a tool for phylogenetic analysis and post-analysis of large phylogenies. *Bioinformatics* 30: 1312–1313.
