# Supplementary material for: Evolutionary constraint on a core winter proteome across independently cold-adapted PACMAD grasses
Source: PLoS One. 2026 Oct 14;21(10):e0358458. doi: 10.1371/journal.pone.0358458 (PMC13667808; doi:10.1371/journal.pone.0358458)
Supplement: Supplementary file 13 [file pone.0358458.s027.docx]

**Manuscript Title:** Constrained evolution of a core winter proteome across independently cold-adapted PACMAD grasses

**Manuscript Number:** PONE-D-26-14249

The manuscript “Constrained evolution of a core winter proteome across independently cold-adapted PACMAD grasses” presents a valuable contribution to understanding the evolution of cold tolerance within the PACMAD clade. The use of quantitative proteomics in a "common-garden" experimental design is a high-value methodological strength. The work is ambitious and well-grounded; however, it lacks transparency regarding technical details essential for reproducibility and presents gaps in the interpretation of some statistical data. While the writing is of high quality, certain terms and sections should be rewritten to improve the article’s impact.

**Introduction and Abstract**

**#1 - L32 (Abstract):** The term "remains unknown" is excessively broad. Suggestion: "...remains to be elucidated at the protein level" or "...remains poorly understood."

**#2-**The text explains the expansion into temperate latitudes well. I suggest adding a sentence regarding the economic importance of PACMAD grasses (e.g., maize, sorghum, sugarcane) at the beginning to frame the study's global impact for the editor.

**#3 -** The rationale for choosing rhizomes over leaves should be explicitly clarified in the introduction. It is critical to emphasize that the rhizome is the key organ for winter survival and perennity, justifying the focus on a "survival strategy" rather than an immediate "stress response."

**#4 -** The hypothesis currently appears "hidden" within the text. I recommend using a standalone sentence or a paragraph starting with: *"Here, we test the hypothesis that..."* to improve readability.

**Methodology**

**#5 -** For the phrase "in a common garden exposed to sustained sub-zero temperatures," I suggest using "common-garden experiment," which is the standard technical term for this design.

**#6- (#L199):** There is a significant lack of detail in the main text regarding the LC-MS/MS setup, which hinders reproducibility. Please include: Column specifications (brand, C18, length, diameter, and particle size) and whether a trap column was used. Solvents used and the exact chromatographic gradient (e.g., % of acetonitrile increase over time).

**#7 -** Provide the collision energy (HCD/CID), injection time, and precursor isolation windows for TMT. Without these, the MS³ data cannot be replicated.

**#8 -** Was any quality control or mass calibration monitoring performed during the runs?

**#9 -**Specify the source of the FASTA files for each species (e.g., Phytozome, NCBI, UniProt), the number of entries per database, and if a common contaminant database was included.

**#10 -** While Proteome Discoverer is mentioned, it is unclear if FDR (False Discovery Rate) filters were applied at both peptide and protein levels (standard <1%). Furthermore, clarify the quantification algorithm: were raw TMT intensities used, or was protein-level normalization applied?

**Results and Discussion**

**#11 -** Winter mortality reduced the biological replicates of *S. nutans* to n=2$. How did this affect the statistical power of the Tukey tests or linear models? Have the authors performed a sensitivity analysis to ensure this species is not distorting orthogroup correlations? Clarify how intra-group variance was handled to avoid false positives.

**#12 -** The authors state that the proteome is more conserved than the transcriptome but do not fully explore the biological reason. Is mRNA more "plastic" for rapid adaptation, while rhizome proteins require structural stability? Discussing the concept of "post-translational buffering" would enrich the manuscript.

**#13 -**The discussion should emphasize that rhizomes are subterranean organs. Soil insulation provides a different environment than leaves; therefore, conservation findings might be specific to this "dormancy organ."

**#14 -**The findings regarding the 11-amino acid repeats are compelling. However, it is unclear if total LEA3 abundance compensates for the lower number of repeats in sensitive species. Does maize (sensitive) produce *more* copies of a "short" protein or *fewer* copies? This distinction is vital for discussing energy efficiency and evolution.

**#15-** I suggest adding a graphic (such as an UpSet plot or Venn diagram) to clearly show the exact intersection of shared proteins among the five species.

**#16 -** A visualization (e.g., Bubble Plot) showing which metabolic pathways are most conserved across species beyond LEA3 would add significant value and novel information to the literature.

**General Comments**

**#17-** The manuscript relies heavily on the passive voice (e.g., "Proteomes were quantified..."). I recommend using the active voice (e.g., "We quantified...") to make the text more direct and engaging.

**#18-** While the focus on conservation is excellent, the authors could explore "Conservation Outliers"—proteins highly responsive in only one species despite belonging to conserved families—to provide a counterpoint to the "constrained evolution" thesis.

In summary, this manuscript provides a valuable and original contribution to plant evolutionary biology by demonstrating a conserved "core winter proteome" in PACMAD grasses. The application of TMT-MS³ technology and the functional analysis of the LEA3 protein provide a strong mechanistic basis for the findings. The study is well-grounded and has high potential for impact. However, the manuscript currently lacks transparency regarding its methodology. To ensure reproducibility, the authors must explicitly detail the LC-MS/MS parameters and database sources used. Furthermore, the statistical limitations of *S. nutans* and the biological implications of protein-transcript decoupling require a more rigorous and transparent discussion. Once these gaps are addressed, the manuscript will be well-suited for publication.
