# Supplementary material for: Evolutionary constraint on a core winter proteome across independently cold-adapted PACMAD grasses
Source: PLoS One. 2026 Oct 14;21(10):e0358458. doi: 10.1371/journal.pone.0358458 (PMC13667808; doi:10.1371/journal.pone.0358458)
Supplement: Supplementary file 16 [file pone.0358458.s029.docx]

**Response to Reviewers**

**Manuscript: PONE-D-26-14249**

Evolutionary constraint on a core winter proteome across independently cold-adapted PACMAD grasses

Dear Dr. Srinivasan,

We thank you and the two reviewers for the thoughtful and constructive evaluation of our manuscript. Below we provide a point-by-point response to each comment. Reviewer comments are reproduced in black; our responses appear in blue immediately below each.

Sincerely,

Elad Oren, on behalf of all authors

**Editorial requirements**

**Comment 1:**

Please ensure that your manuscript meets PLOS ONE's style requirements, including those for file naming.

The manuscript has been reformatted to follow the PLOS ONE main body and title-page templates. The reference list has been converted to Vancouver numbered format and Supporting Information citations now follow PLOS ONE convention (S1 Fig, S1 Table).

**Comment 2:**

PLOS ONE has specific guidelines on code sharing for submissions in which author-generated code underpins the findings.

All author-generated code is available at <https://github.com/elor77/PACMAD-Rhizome-Proteomics>, with the repository URL provided in the Data Availability section. We have opted for the GitHub deposit rather than a zipped Supporting Information file because GitHub provides a more durable, discoverable, and citable repository for the analysis pipelines, consistent with PLOS ONE's recommendations for code sharing.

**Comment 3:**

Please provide an amended Funding Statement that declares all the funding or sources of support received during this study, and include the statement "There was no additional external funding received for this study."

The Funding Statement has been amended to include all funding sources and the requested sentence verbatim. The amended statement is reproduced in the cover letter.

**Comment 4:**

Please remove any funding-related text from the Acknowledgments section.

All funding-related text has been removed from the Acknowledgments section.

**Comment 5:**

We are unable to open your Supporting Information file PACMAD_Rhizome_Proteomics_Code_S1-S3.zip.

Apologies for the corrupted upload. We have replaced the zipped supplementary code file with a permanent GitHub deposit (<https://github.com/elor77/PACMAD-Rhizome-Proteomics>). The repository is referenced in the Data Availability section.

**Reviewer 1**

***Introduction and Abstract***

**Comment 1: "remains unknown" too broad**

L32 (Abstract): The term "remains unknown" is excessively broad. Suggestion: "...remains to be elucidated at the protein level" or "...remains poorly understood."

Adopted. The Abstract now reads "...remains poorly understood at the protein level".

**Comment 2: Economic-importance opening**

Add a sentence regarding the economic importance of PACMAD grasses (e.g., maize, sorghum, sugarcane) at the beginning to frame the study's global impact.

Adopted. The Introduction now opens with: "Grasses of the PACMAD clade, including maize, sorghum, sugarcane, the millets, and the bioenergy grass *Miscanthus*, supply a substantial fraction of global cereal, forage, and biofuel production."

**Comment 3: Rationale for rhizomes vs. leaves**

The rationale for choosing rhizomes over leaves should be explicitly clarified. Emphasize that the rhizome is the key organ for winter survival and perennity, justifying the focus on a "survival strategy" rather than an immediate "stress response."

Adopted. We added a new paragraph to the Introduction [L97] explicitly justifying the focus on rhizome tissue: prior molecular work on grass cold biology has focused on the acute leaf-level cold-shock response, but inter-annual survival in perennial PACMAD grasses depends on the rhizome, a specialized below-ground stem that overwinters intact. Because the rhizome is buffered by soil from diurnal extremes and its overwintering is a sustained, months-long state of dormancy, we frame the molecular biology of winter survival as a question about a dormant subterranean organ and the survival strategy it deploys, not an immediate stress response.

**Comment 4: Hypothesis stated explicitly**

The hypothesis currently appears "hidden" within the text. I recommend using a standalone sentence or a paragraph starting with: "Here, we test the hypothesis that..."

Adopted. The Introduction now closes with explicit hypothesis sentences [L118]: "Here, we test the hypothesis that independently cold-adapted PACMAD lineages converge on a shared core winter proteome, defined as the set of orthogroups differentially abundant between summer-active and winter-dormant rhizomes in two or more of the five species examined. We further hypothesize that the magnitude of cold-induced proteomic responses is consistent with constraint imposed by the shared physiological requirements of freezing tolerance."

***Methodology***

**Comment 5: "common-garden experiment"**

For the phrase "in a common garden exposed to sustained sub-zero temperatures," use "common-garden experiment," which is the standard technical term.

Adopted. "Common garden" has been replaced with "common-garden experiment" throughout the manuscript and figure captions.

**Comment 6: LC-MS/MS column and gradient details**

There is a significant lack of detail in the main text regarding the LC-MS/MS setup. Please include: column specifications (brand, C18, length, diameter, particle size); whether a trap column was used; solvents and the exact chromatographic gradient.

Adopted. The LC description has been substantially expanded in S1 Methods (new subsection "Mass Spectrometric Acquisition"). Peptides were loaded onto a PepMap C-18 reversed-phase nano-trap column (5 µm, 100 µm × 20 mm; Dionex) and separated on a PepMap C-18 analytical column (2 µm, 75 µm × 25 cm). Mobile phase A was 2% acetonitrile in 0.1% aqueous formic acid; mobile phase B was 95% acetonitrile in 0.1% formic acid. The 162-min run used a 120-min gradient from 5% to 33% B, an 8-min ramp to 90% B, a 9-min hold at 90% B, and a 25-min re-equilibration. Flow rate was 300 nL/min and the column oven was maintained at 35 °C.

**Comment 7: MS3 acquisition parameters**

Provide the collision energy (HCD/CID), injection time, and precursor isolation windows for TMT. Without these, the MS3 data cannot be replicated.

Full MS2 and MS3 acquisition parameters have been added to S1 Methods. MS2 fragmentation used CID in the linear ion trap (NCE 30%, isolation width 0.7 m/z, max injection time 50 ms, AGC target 1 × 10⁴). MS3 fragmentation of the top 10 SPS precursors used HCD in the Orbitrap (NCE 55%, isolation width 1.1 m/z, max injection time 120 ms, AGC target 1 × 10⁵). The Real-Time Search node searched MS2 spectra against the species-specific reference proteome with TMTpro and carbamidomethyl as static modifications and methionine oxidation as a variable modification, with up to one missed cleavage and two variable modifications per peptide. No inclusion list was used.

**Comment 8: QC and mass calibration**

Was any quality control or mass calibration monitoring performed during the runs?

Yes. The new "Instrument Quality Control and Calibration" subsection in S1 Methods describes the workflow: mass accuracy was monitored using the protonated hexakis(2,2-dimethoxypropane)cyclodisiloxane lock-mass ion at m/z 445.120029 with a 5 ppm SD tolerance prior to each sample injection; when the lock-mass mass error exceeded 5 ppm, the instrument was recalibrated using the Pierce FlexMix calibration solution. *Escherichia coli* tryptic digest and BSA were run as quality-assurance samples prior to each sample series, with instrument performance evaluated via the TIC, base peak chromatograms of the *E. coli* digest, and extracted ion chromatograms of representative BSA peptides.

**Comment 9: FASTA sources and contaminant database**

Specify the source of the FASTA files for each species (e.g., Phytozome, NCBI, UniProt), the number of entries per database, and if a common contaminant database was included.

The per-species FASTA sources, version codes, and Data Availability URLs (MaizeGDB and JGI Data Portal) were already provided in the originally submitted manuscript ("Peptide Mapping and Reference Proteome Assignment" subsection and Data Availability section). We have now added: (i) [L249] per-database protein-entry counts (197,551 for *A. gerardi*; 67,789 for *M. sinensis*; 95,264 for *P. virgatum*; 123,723 for *S. nutans*; 65,151 for *T. dactyloides*); and (ii) [L255] explicit confirmation that all databases were supplemented with the cRAP common contaminants database for the Sequest HT search.

**Comment 10: FDR and TMT quantification**

It is unclear if FDR filters were applied at both peptide and protein levels (standard <1%). Clarify the quantification algorithm: were raw TMT intensities used, or was protein-level normalization applied?

The originally submitted M&M states that Proteome Discoverer 2.5 was used with the Sequest HT engine, that peptide-spectral matches were filtered at 1% FDR (Percolator), that protein identifications required at least two distinct peptides, that protein quantification was based on summed reporter ion intensities of unique and razor peptides, that intensities were normalized using the "total peptide amount" method, and that statistical testing used ANOVA followed by Tukey HSD with Benjamini-Hochberg correction. To address the reviewer's specific questions: (i) FDR filters were applied at both peptide and protein levels (1% strict FDR), now stated explicitly in S1 Methods; (ii) reporter ions were quantified at the MS3 level (HCD, FTMS analyzer, 20 ppm integration tolerance), filtered at co-isolation threshold 50%, average reporter S/N at least 10, and SPS mass matches at least 65%, with reporter intensities of unique + razor peptides summed per protein and normalized across channels by Total Peptide Amount. Modified peptides (deamidated N/Q) were excluded from pairwise ratio calculation. Full parameter list is now provided in S1 Methods.

***Results and Discussion***

**Comment 11: S. nutans n=2 — sensitivity analysis**

Winter mortality reduced the biological replicates of S. nutans to n=2. How did this affect statistical power? Have the authors performed a sensitivity analysis to ensure this species is not distorting orthogroup correlations? Clarify how intra-group variance was handled.

We thank the reviewer; this concern overlaps with Reviewer 2's major comment on sample-size imbalance, and we address both together. The originally submitted Results already reported a sensitivity analysis: "This result was robust to the exclusion of *S. nutans*, which had fewer biological replicates (n = 2 per season; *P* = 0.002)." To address the reviewer's concern more fully, we have added a Limitations sentence explicitly framing the replication imbalance: *S. nutans* yields wider per-species confidence intervals than the other species; orthogroup-level fold-change summarization buffers, but does not eliminate, replication-driven variance; and the *Tripsacum* genus is represented as a single collapsed entry in cross-species tests to avoid pseudo-replication.

**Comment 12: Post-translational buffering**

The proteome being more conserved than the transcriptome is not fully explored. Discussing the concept of "post-translational buffering" would enrich the manuscript.

Adopted. The originally submitted Discussion already invoked this framework via Cope et al. (ref [29]), explaining that protein abundances are under stronger stabilizing selection than mRNA abundances. We have now strengthened this paragraph [L623] connecting the buffering argument specifically to the dormancy-organ context: "Such buffering is plausibly under particularly strong selection in an overwintering rhizome, which must maintain a functional, dormancy-competent proteome over months rather than minutes."

**Comment 13: Subterranean / soil-insulated context**

Emphasize that rhizomes are subterranean organs. Soil insulation provides a different environment than leaves; conservation findings might be specific to this "dormancy organ."

Adopted. We have added a Discussion paragraph that makes this point explicitly: soil thermally buffers below-ground organs against the diurnal extremes that aerial tissues experience, and the dormant rhizome inhabits a relatively stable, sustained sub-zero environment rather than the acute, fluctuating cold stress imposed on leaves. The conservation patterns reported here are therefore specific to a dormancy organ and may not generalize to leaf-level cold-shock responses.

**Comment 14: LEA3 abundance vs. 11-aa repeat number**

It is unclear if total LEA3 abundance compensates for the lower number of repeats in sensitive species. Does maize (sensitive) produce more copies of a "short" protein or fewer copies?

We thank the reviewer for this perceptive question. The originally submitted Discussion identified three factors shaping cryoprotective efficacy (amphipathic helix length per protein, total protein abundance via paralog accumulation, and repeat composition), and noted that *Andropogon gerardi* and *Panicum virgatum* accumulate multiple LEA3 paralogs while maize and *M. giganteus* do not. To make this answer explicit, we have added: "Maize encodes a single LEA3 ortholog, with no copy-number expansion to offset the shorter, more hydrophobic protein architecture." The single-paralog result was confirmed by DIAMOND BLASTp searches against the maize B73 NAM v5 reference proteome.

**Comment 15: UpSet / Venn of shared proteins**

Add a graphic (UpSet plot or Venn diagram) showing the exact intersection of shared proteins among the five species.

An UpSet plot of shared DAP intersections across species is provided as S6 Fig. We agree the figure deserves more prominent treatment and have added an explicit Results-section sentence pointing to it [L396]: "The full pattern of pairwise, triple, quadruple, and five-way DAP intersections across species is shown in S6 Fig."

**Comment 16: Pathway breadth visualization**

A visualization (e.g., Bubble Plot) showing which metabolic pathways are most conserved across species beyond LEA3 would add significant value.

Fig 3a in the originally submitted manuscript already presents this information: a mosaic plot of cold-related functional categories across all five species, showing which pathways (protein folding, metabolism/osmoregulation, detoxification/ROS scavenging, lipid metabolism, membrane stability, and others) are conserved versus species-specific in their relative investment. The mosaic plot was chosen over a bubble/dotplot because it integrates relative abundance and category presence within each species in a single panel. The Results narrative identifies the conserved categories beyond LEA3 (HSPs, ROS scavenging, putative chitinase-like antifreeze proteins) and discusses lineage-specific allocation.

***General***

**Comment 17: Active voice**

The manuscript relies heavily on the passive voice. Recommend active voice to make the text more direct and engaging.

Adopted in part. We have made selective active-voice conversions where the text read as overly impersonal. We have retained passive voice in Methods sections covering routine protocols (sample lysis, digestion, mass-spectrometric acquisition), where it is the field-standard convention. We hope this approach is acceptable.

**Comment 18: Conservation outliers**

Explore "Conservation Outliers" — proteins highly responsive in only one species despite belonging to conserved families — to provide a counterpoint to the "constrained evolution" thesis.

Adopted. We have added a Discussion paragraph and a new S13 Table identifying conservation outliers: orthogroups whose response in a single species substantially exceeds the cross-species median, despite belonging to functional families otherwise represented in two or more species. Notable examples highlighted in the text include a Temperature-Induced Lipocalin orthogroup elevated specifically in *Tripsacum*, a chitinase-like GH18 protein elevated in *A. gerardi*, and ureidoglycolate hydrolase elevated only in *M. giganteus*. We highlight these outliers as biologically meaningful exceptions rather than as a counter-thesis; the constrained-magnitude pattern is not contingent on their presence.

**Reviewer 2**

***Major comments***

**Major Comment 1: "Constrained evolution" overstated**

The authors claim that protein-level responses reflect "constrained evolution." This claim seems overstated. While the observed correlations are compelling, they do not uniquely imply evolutionary constraint.

We agree, and have made this the central interpretive revision. The originally submitted Abstract used "is consistent with evolutionary constraints on protein-level response magnitude" rather than asserting causation; we have now extended this consistency framing to the body. Two remaining instances of causal language ("is governed by") have been softened to "is consistent with." We have not removed the mechanistic hypothesis but now present it as one of several non-mutually-exclusive explanations (see Major Comment 2). The manuscript title has been updated from "Constrained evolution of..." to "Evolutionary constraint on..." to reflect the consistency framing throughout the body, now describing constraint as a property of the proteome we observed rather than a sweeping evolutionary claim.

**Major Comment 2: Alternative explanations not sufficiently discussed**

The alternative explanations such as shared physiological limits, environmental uniformity, and statistical thresholds are not sufficiently discussed.

Adopted. The Discussion now contains a dedicated paragraph enumerating three non-mutually-exclusive explanations for the observed cross-species correlations [L590]: (i) *shared physiological limits* — the biophysical demands of freezing tolerance impose a narrow set of viable molecular responses, generating cross-species correlation independently of evolutionary history; (ii) *environmental uniformity* — the common-garden experiment exposes all species to identical thermal cues, which alone can elicit correlated responses from independently evolved sensing pathways; and (iii) *statistical-threshold artifacts* — the |log2FC| at least 1 selection criterion preferentially retains proteins with strong, reproducible responses, biasing toward shared signal. We discuss honestly which alternatives our controls (magnitude-matched permutation; Brownian-motion permulation) address, and which they do not.

**Major Comment 3: Sample-size imbalance**

There seems to be imbalance in sample size across species, for example extensive Tripsacum sampling vs. limited replication in other species, which might challenge the identification of differentially abundant proteins and shared responses.

We address this concern jointly with Reviewer 1 Comment 11. *Tripsacum* accessions (Trip19, Trip22) were collapsed into a single *Tripsacum* entry for cross-species analyses to avoid pseudo-replication. Per-species DAP calling uses replication-aware tests; orthogroup-level fold-change summarization buffers some of the imbalance. The originally submitted Results [L584] documents that the headline cross-species correlation is robust to exclusion of *S. nutans* (the species with reduced replication; *P* = 0.002), and we have added an explicit Limitations sentence framing the replication imbalance.

**Major Comment 4: LEA3 not experimentally validated**

The key findings, particularly regarding LEA3, were not experimentally validated. The authors may rephrase the conclusions and acknowledge the limitation.

Adopted. The originally submitted Discussion includes the caveat that "freezing survival involves the coordinated action of numerous protective mechanisms, and restoring a single protein's architecture in a freezing-sensitive species would be unlikely to confer full tolerance." To make the validation gap fully explicit, we have softened LEA3 functional language throughout the Discussion and added a closing sentence [L664]: "Direct experimental validation of the proposed LEA3 contribution, for example through heterologous expression, repeat-number perturbation, or *in vitro* cryoprotection assays, is required to establish causation and is beyond the scope of the present study."

***Minor comments***

**Minor Comment 1: Define key terms**

Define key terms such as "core winter proteome" and "constraint on response magnitude."

Adopted. The Introduction now defines "core winter proteome" on first body use as "the set of orthogroups that are differentially abundant between summer-active and winter-dormant rhizomes in two or more of the five species examined." "Constraint on response magnitude" is defined operationally in the Discussion as "a higher cross-species correlation in cold-induced fold-change among shared cold-responsive orthogroups than among proteins responsive in at most one species."

**Minor Comment 2: |log2FC| ≥ 1 threshold**

Clarify threshold selection (|log2FC| ≥ 1).

This is already addressed in the originally submitted M&M, where we justify the |log2FC| at least 1 cutoff as a stringent threshold appropriate for a multi-species field study with inherent biological variability, prioritizing high-confidence candidates over minor fluctuations that may reflect transient environmental noise. The same paragraph notes that this approach addresses the statistical power imbalance between the deeply sampled *Tripsacum* dataset and the smaller per-species datasets.

**Minor Comment 3: Batch correction and normalization detail**

Provide more detail on batch correction and normalization.

The Methods describe within-plex normalization (Total Peptide Amount in PD 2.5) and the multi-batch *Tripsacum* 2022 strategy (within-batch testing followed by Fisher's-method P-value combination, retention of proteins quantified in at least 3 of 4 batches). Cross-species normalization rests on orthogroup-level fold-change summarization rather than direct cross-plex abundance correction. The full implementation is available in the GitHub repository linked in Data Availability.

We are grateful to both reviewers and to the academic editor for the time invested in this manuscript. We hope these revisions adequately address the points raised.

Sincerely,

Elad Oren, Ph.D.

on behalf of all authors
