# Supplementary material for: Evolutionary constraint on a core winter proteome across independently cold-adapted PACMAD grasses
Source: PLoS One. 2026 Oct 14;21(10):e0358458. doi: 10.1371/journal.pone.0358458 (PMC13667808; doi:10.1371/journal.pone.0358458)
Supplement: Supplementary file 17 [file pone.0358458.s030.docx]

**Response to Reviewers**

**Manuscript: PONE-D-26-14249R1**

Evolutionary constraint on a core winter proteome across independently cold-adapted PACMAD grasses

Dear Dr. Vennapusa,

We thank you for taking on the editorial handling of this manuscript and for your careful reading of the revised version. We are also grateful to both reviewers, who indicated that all of their previous comments have been addressed. Below we provide a point-by-point response to each comment. Editor comments are reproduced in black; our responses appear in blue immediately below each.

**Editorial requirements**

**Comment 1:**

Please ensure that the affiliations, email for the corresponding author (Elad Oren) are correctly specified. Should it be Newe Ya'ar Research Center, ARO, Israel, Cornell University, or both?

Both. We have corrected this. E.O. is now listed with both affiliations, and the correspondence address and email have been updated accordingly.

**Comment 2:**

In the methods section, please specify which plant species belong to each of the following clades: Panicoideae, Aristidoideae, Chloridoideae, Micrairoideae, Arundinelloideae, and Danthonioideae (PACMAD).

Adopted, and we thank the editor for raising this. We have added a clarification to the opening of the Materials and Methods. While all sampled taxa fall within Panicoideae, the PACMAD framing describes the evolutionary context of repeated cold adaptation, not the taxonomic breadth of our sampling. We have also corrected the subfamily name in the Abstract and Introduction to Arundinoideae.

**Comment 3:**

Maintain a uniform format when citing the table or figure numbers, and check the journal guidelines. Should it be Supporting Information S1 Methods, S1 Fig, or Fig S1?

Corrected according to the PLOS ONE convention.

**Comment 4:**

Please provide citations for the F₂ seedlings used in RNA sequencing and RNA extraction, along with a brief description of the method. Additionally, cite the references for the protein extraction and digestion method.

Adopted. We now cite El-Walid et al. [32] for the F₂ germplasm, crossing design, and freezing screen, and have expanded the "F₂ Seedlings for RNA sequencing" subsection with a brief description of the growth, cold-acclimation, and screening conditions; this companion preprint was posted after our original submission, which is why it could not be cited previously. We have also clarified the sampling sequence. RNA extraction follows Gault et al. [43], as before. For the proteomics, we have extended the existing citation to Fu et al. [34], which describes the S-Trap digestion, TMTpro labeling, and fractionation strategy used here, and added Isaacson et al. [33] for the phenol/ammonium acetate–methanol extraction. Full buffer compositions remain in S1 Methods.

**Comment 5:**

Ensure that the referencing format is consistent throughout both the manuscript and supporting information—either numbering or author names. For example, line 276: should it be "Cope et al. (2025)" or a reference number?

Corrected. The manuscript uses the PLOS ONE Vancouver numbered format throughout.

**Comment 6:**

Lastly, did the authors perform any qRT-PCR or other functional validation studies to confirm the function or variation of specific LEA3 genes or proteins?

No. No qRT-PCR, heterologous expression, or in vitro cryoprotection assays were performed. The study is a comparative quantitative-proteomics survey, and our LEA3 findings are framed accordingly as observations of cross-species abundance and structural architecture rather than functional claims; following Reviewer 2's Major Comment 4 in the previous round, we softened the functional language throughout the Discussion and stated explicitly that direct validation is required to establish causation and is beyond the present scope [L677].

We have reviewed the full reference list for completeness and confirm that no cited work has been retracted.We hope these revisions adequately address the points raised.

Sincerely,

Elad Oren, on behalf of all authors
