# Supplementary material for: Evolutionary constraint on a core winter proteome across independently cold-adapted PACMAD grasses
Source: PLoS One. 2026 Oct 14;21(10):e0358458. doi: 10.1371/journal.pone.0358458 (PMC13667808; doi:10.1371/journal.pone.0358458)
Supplement: Peer review history

# Peer review: Decision Letter 0

**DOI:** 10.1371/journal.pone.0358458.r001

**Authors:** Balamurugan Srinivasan

5 May 2026

Dear Dr. Oren,

Thank you for submitting your manuscript to PLOS ONE. After careful consideration, we feel that it has merit but does not fully meet PLOS ONE’s publication criteria as it currently stands. Therefore, we invite you to submit a revised version of the manuscript that addresses the points raised during the review process.

Please submit your revised manuscript by Jun 19 2026 11:59PM. If you will need more time than this to complete your revisions, please reply to this message or contact the journal office at plosone@plos.org. When you're ready to submit your revision, log on to https://www.editorialmanager.com/pone/ and select the 'Submissions Needing Revision' folder to locate your manuscript file.

- A letter that responds to each point raised by the academic editor and reviewer(s). You should upload this letter as a separate file labeled 'Response to Reviewers'.
- A marked-up copy of your manuscript that highlights changes made to the original version. You should upload this as a separate file labeled 'Revised Manuscript with Track Changes'.
- An unmarked version of your revised paper without tracked changes. You should upload this as a separate file labeled 'Manuscript'.

If you would like to make changes to your financial disclosure, please include your updated statement in your cover letter. Guidelines for resubmitting your figure files are available below the reviewer comments at the end of this letter.

If applicable, we recommend that you deposit your laboratory protocols in protocols.io to enhance the reproducibility of your results. Protocols.io assigns your protocol its own identifier (DOI) so that it can be cited independently in the future. For instructions see: https://journals.plos.org/plosone/s/submission-guidelines#loc-laboratory-protocols. Additionally, PLOS ONE offers an option for publishing peer-reviewed Lab Protocol articles, which describe protocols hosted on protocols.io. Read more information on sharing protocols at https://plos.org/protocols?utm_medium=editorial-email&utm_source=authorletters&utm_campaign=protocols.

As the corresponding author, your ORCID iD is verified in the submission system and will appear in the published article. PLOS supports the use of ORCID, and we encourage all coauthors to register for an ORCID iD and use it as well. Please encourage your coauthors to verify their ORCID iD within the submission system before final acceptance, as unverified ORCID iDs will not appear in the published article. *Only*  the individual author can complete the verification step; PLOS staff *cannot*  verify ORCID iDs on behalf of authors.

We look forward to receiving your revised manuscript.

Kind regards,

Balamurugan Srinivasan

Academic Editor

PLOS One

Journal Requirements:

When submitting your revision, we need you to address these additional requirements.

1. Please ensure that your manuscript meets PLOS ONE's style requirements, including those for file naming. The PLOS ONE style templates can be found at

https://journals.plos.org/plosone/s/file?id=wjVg/PLOSOne_formatting_sample_main_body.pdf and

https://journals.plos.org/plosone/s/file?id=ba62/PLOSOne_formatting_sample_title_authors_affiliations.pdf.

2. Please note that PLOS One has specific guidelines on code sharing for submissions in which author-generated code underpins the findings in the manuscript. In these cases, we expect all author-generated code to be made available without restrictions upon publication of the work. Please review our guidelines at https://journals.plos.org/plosone/s/materials-and-software-sharing#loc-sharing-code and ensure that your code is shared in a way that follows best practice and facilitates reproducibility and reuse.

3. Thank you for stating in your Funding Statement:

“This research was supported by the National Science Foundation under Grant Number 1822330, and the U.S. Department of Agriculture–Agricultural Research Service (USDA-ARS) under Project Number 5030-21000-072-00-D (Corn Insects and Crop Genetics Research Unit, Ames, Iowa), Project Number 6066-21310-005-00-D (Genomics and Bioinformatics Research Unit, Stoneville, MS), Project Number 8062-21000-043-00-D (Plant, Soil and Nutrition Research Unit, Ithaca, NY), and the United States - Israel Binational Agricultural Research and Development Fund, Vaadia-BARD Postdoctoral Fellowship Award No. FI-628-2022. C.M.G. was supported by the National Science Foundation Postdoctoral Research Fellowship in Biology under Grant No. 1523861. Research in the lab of J.H. is supported by USDA NIFA (2022-67013-37040). Q.F. was supported by HHMI Transformative Technology 2019 program for the Orbitrap Eclipse system. The funders had no role in study design, data collection and analysis, decision to publish, or preparation of the manuscript. “

Please provide an amended statement that declares *all* the funding or sources of support (whether external or internal to your organization) received during this study, as detailed online in our guide for authors at http://journals.plos.org/plosone/s/submit-now.  Please also include the statement “There was no additional external funding received for this study.” in your updated Funding Statement.

Please include your amended Funding Statement within your cover letter. We will change the online submission form on your behalf.

4. Thank you for stating the following in the Acknowledgments Section of your manuscript:

“This research was supported by the National Science Foundation under Grant Number 1822330, and the U.S. Department of Agriculture–Agricultural Research Service (USDA-ARS) under Project Number 5030-21000-072-00-D (Corn Insects and Crop Genetics Research Unit, Ames, Iowa), Project Number 6066-21310-005-00-D (Genomics and Bioinformatics Research Unit, Stoneville, MS), Project Number 8062-21000-043-00-D (Plant, Soil and Nutrition Research Unit, Ithaca, NY), and the United States - Israel Binational Agricultural Research and Development Fund, Vaadia-BARD Postdoctoral Fellowship Award No. FI-628-2022. C.M.G. was supported by the National Science Foundation Postdoctoral Research Fellowship in Biology under Grant No. 1523861. Research in the lab of J.H. is supported by USDA NIFA (2022-67013-37040). Q.F. was supported by HHMI Transformative Technology 2019 program for the Orbitrap Eclipse system.”

We note that you have provided additional information within the Acknowledgements Section that is not currently declared in your Funding Statement. Please note that funding information should not appear in the Acknowledgments section or other areas of your manuscript. We will only publish funding information present in the Funding Statement section of the online submission form.

Please remove any funding-related text from the manuscript and let us know how you would like to update your Funding Statement. Currently, your Funding Statement reads as follows:

“This research was supported by the National Science Foundation under Grant Number 1822330, and the U.S. Department of Agriculture–Agricultural Research Service (USDA-ARS) under Project Number 5030-21000-072-00-D (Corn Insects and Crop Genetics Research Unit, Ames, Iowa), Project Number 6066-21310-005-00-D (Genomics and Bioinformatics Research Unit, Stoneville, MS), Project Number 8062-21000-043-00-D (Plant, Soil and Nutrition Research Unit, Ithaca, NY), and the United States - Israel Binational Agricultural Research and Development Fund, Vaadia-BARD Postdoctoral Fellowship Award No. FI-628-2022. C.M.G. was supported by the National Science Foundation Postdoctoral Research Fellowship in Biology under Grant No. 1523861. Research in the lab of J.H. is supported by USDA NIFA (2022-67013-37040). Q.F. was supported by HHMI Transformative Technology 2019 program for the Orbitrap Eclipse system. The funders had no role in study design, data collection and analysis, decision to publish, or preparation of the manuscript. “

Please include your amended statements within your cover letter; we will change the online submission form on your behalf.

5. We are unable to open your Supporting Information file [PACMAD_Rhizome_Proteomics_Code_S1-S3.zip ]. Please kindly revise as necessary and re-upload.

6. If the reviewer comments include a recommendation to cite specific previously published works, please review and evaluate these publications to determine whether they are relevant and should be cited. There is no requirement to cite these works unless the editor has indicated otherwise.

[Note: HTML markup is below. Please do not edit.]

Reviewers' comments:

Reviewer's Responses to Questions

**Comments to the Author**

1. Is the manuscript technically sound, and do the data support the conclusions?

Reviewer #1: Partly

Reviewer #2: Yes

**********

2. Has the statistical analysis been performed appropriately and rigorously?

Reviewer #1: Yes

Reviewer #2: Yes

**********

3. Have the authors made all data underlying the findings in their manuscript fully available?

The PLOS Data policy

Reviewer #1: Yes

Reviewer #2: Yes

**********

4. Is the manuscript presented in an intelligible fashion and written in standard English?

Reviewer #1: Yes

Reviewer #2: Yes

**********

Reviewer #1: The manuscript “Constrained evolution of a core winter proteome across independently cold-adapted PACMAD grasses” presents a valuable contribution to understanding the evolution of cold tolerance within the PACMAD clade. The use of quantitative proteomics in a "common-garden" experimental design is a high-value methodological strength. The work is ambitious and well-grounded; however, it lacks transparency regarding technical details essential for reproducibility and presents gaps in the interpretation of some statistical data. While the writing is of high quality, certain terms and sections should be rewritten to improve the article’s impact.

Introduction and Abstract

#1 - L32 (Abstract): The term "remains unknown" is excessively broad. Suggestion: "...remains to be elucidated at the protein level" or "...remains poorly understood."

#2-The text explains the expansion into temperate latitudes well. I suggest adding a sentence regarding the economic importance of PACMAD grasses (e.g., maize, sorghum, sugarcane) at the beginning to frame the study's global impact for the editor.

#3 - The rationale for choosing rhizomes over leaves should be explicitly clarified in the introduction. It is critical to emphasize that the rhizome is the key organ for winter survival and perennity, justifying the focus on a "survival strategy" rather than an immediate "stress response."

#4 - The hypothesis currently appears "hidden" within the text. I recommend using a standalone sentence or a paragraph starting with: "Here, we test the hypothesis that..." to improve readability.

Methodology

#5 - For the phrase "in a common garden exposed to sustained sub-zero temperatures," I suggest using "common-garden experiment," which is the standard technical term for this design.

#6- (#L199): There is a significant lack of detail in the main text regarding the LC-MS/MS setup, which hinders reproducibility. Please include: Column specifications (brand, C18, length, diameter, and particle size) and whether a trap column was used. Solvents used and the exact chromatographic gradient (e.g., % of acetonitrile increase over time).

#7 - Provide the collision energy (HCD/CID), injection time, and precursor isolation windows for TMT. Without these, the MS³ data cannot be replicated.

#8 - Was any quality control or mass calibration monitoring performed during the runs?

#9 -Specify the source of the FASTA files for each species (e.g., Phytozome, NCBI, UniProt), the number of entries per database, and if a common contaminant database was included.

#10 - While Proteome Discoverer is mentioned, it is unclear if FDR (False Discovery Rate) filters were applied at both peptide and protein levels (standard <1%). Furthermore, clarify the quantification algorithm: were raw TMT intensities used, or was protein-level normalization applied?

Results and Discussion

#11 - Winter mortality reduced the biological replicates of S. nutans to n=2$. How did this affect the statistical power of the Tukey tests or linear models? Have the authors performed a sensitivity analysis to ensure this species is not distorting orthogroup correlations? Clarify how intra-group variance was handled to avoid false positives.

#12 - The authors state that the proteome is more conserved than the transcriptome but do not fully explore the biological reason. Is mRNA more "plastic" for rapid adaptation, while rhizome proteins require structural stability? Discussing the concept of "post-translational buffering" would enrich the manuscript.

#13 -The discussion should emphasize that rhizomes are subterranean organs. Soil insulation provides a different environment than leaves; therefore, conservation findings might be specific to this "dormancy organ."

#14 -The findings regarding the 11-amino acid repeats are compelling. However, it is unclear if total LEA3 abundance compensates for the lower number of repeats in sensitive species. Does maize (sensitive) produce more copies of a "short" protein or fewer copies? This distinction is vital for discussing energy efficiency and evolution.

#15- I suggest adding a graphic (such as an UpSet plot or Venn diagram) to clearly show the exact intersection of shared proteins among the five species.

#16 - A visualization (e.g., Bubble Plot) showing which metabolic pathways are most conserved across species beyond LEA3 would add significant value and novel information to the literature.

General Comments

#17- The manuscript relies heavily on the passive voice (e.g., "Proteomes were quantified..."). I recommend using the active voice (e.g., "We quantified...") to make the text more direct and engaging.

#18- While the focus on conservation is excellent, the authors could explore "Conservation Outliers"—proteins highly responsive in only one species despite belonging to conserved families—to provide a counterpoint to the "constrained evolution" thesis.

In summary, this manuscript provides a valuable and original contribution to plant evolutionary biology by demonstrating a conserved "core winter proteome" in PACMAD grasses. The application of TMT-MS³ technology and the functional analysis of the LEA3 protein provide a strong mechanistic basis for the findings. The study is well-grounded and has high potential for impact. However, the manuscript currently lacks transparency regarding its methodology. To ensure reproducibility, the authors must explicitly detail the LC-MS/MS parameters and database sources used. Furthermore, the statistical limitations of S. nutans and the biological implications of protein-transcript decoupling require a more rigorous and transparent discussion. Once these gaps are addressed, the manuscript will be well-suited for publication.

Reviewer #2: This manuscript presents a comparative proteomics study investigating the evolution of freezing tolerance across independently cold-adapted PACMAD grasses. Using a common garden design and orthogroup-based analysis, the authors demonstrate that cold-responsive proteins exhibit higher cross-species conservation in response magnitude than background proteins. The study identifies LEA3 as the only universally elevated ortholog and explores its structural variation as a potential factor underlying differences in freezing tolerance.

The work is novel, methodologically sound, and addresses an important question in plant evolutionary biology and stress physiology. However, several concerns regarding interpretation, statistical balance, and clarity need to be addressed.

Major Concerns

The authors claimed that protein-level responses reflect “constrained evolution”. According to this reviewer this claim seems to be overstated. While the observed correlations are compelling, they do not uniquely imply evolutionary constraint. Kindly clarify this.

It is noted that the alternative explanations for instances shared physiological limits, environmental uniformity, and statistical thresholds are not sufficiently discussed.

It seems to be imbalance in sample size across species for example extensive Tripsacum sampling vs. limited replication in other species, which might challegens the identification of differentially abundant proteins and shared responses. The authors may discuss this.

In the key findings, as claimed by the authors particularly regarding LEA3 were not experimentally validated. Hences, the authors may rephrase the conclusions and acknowledge the limitation.

Minor Concerns

Define key terms such as “core winter proteome” and “constraint on response magnitude”

Clarify threshold selection (|log₂FC| ≥ 1)

Provide more detail on batch correction and normalization

**********

what does this mean?). If published, this will include your full peer review and any attached files.

If you choose “no”, your identity will remain anonymous but your review may still be made public.

**Do you want your identity to be public for this peer review?**  For information about this choice, including consent withdrawal, please see our Privacy Policy

Reviewer #1: No

Reviewer #2: No

**********

[NOTE: If reviewer comments were submitted as an attachment file, they will be attached to this email and accessible via the submission site. Please log into your account, locate the manuscript record, and check for the action link "View Attachments". If this link does not appear, there are no attachment files.]

To ensure your figures meet our technical requirements, please review our figure guidelines: https://journals.plos.org/plosone/s/figures

You may also use PLOS’s free figure tool, NAAS, to help you prepare publication quality figures: https://journals.plos.org/plosone/s/figures#loc-tools-for-figure-preparation.

NAAS will assess whether your figures meet our technical requirements by comparing each figure against our figure specifications.

Attachment Submitted filename: revision_PONE-D-26-14249.docx

# Author comment: Author response to Decision Letter 1

**DOI:** 10.1371/journal.pone.0358458.r002

19 May 2026

Reviewer 1

Introduction and Abstract

Comment 1: "remains unknown" too broad

L32 (Abstract): The term "remains unknown" is excessively broad. Suggestion: "...remains to be elucidated at the protein level" or "...remains poorly understood."

Adopted. The Abstract now reads "...remains poorly understood at the protein level".

Comment 2: Economic-importance opening

Add a sentence regarding the economic importance of PACMAD grasses (e.g., maize, sorghum, sugarcane) at the beginning to frame the study's global impact.

Adopted. The Introduction now opens with: "Grasses of the PACMAD clade, including maize, sorghum, sugarcane, the millets, and the bioenergy grass Miscanthus, supply a substantial fraction of global cereal, forage, and biofuel production."

Comment 3: Rationale for rhizomes vs. leaves

The rationale for choosing rhizomes over leaves should be explicitly clarified. Emphasize that the rhizome is the key organ for winter survival and perennity, justifying the focus on a "survival strategy" rather than an immediate "stress response."

Adopted. We added a new paragraph to the Introduction [L97] explicitly justifying the focus on rhizome tissue: prior molecular work on grass cold biology has focused on the acute leaf-level cold-shock response, but inter-annual survival in perennial PACMAD grasses depends on the rhizome, a specialized below-ground stem that overwinters intact. Because the rhizome is buffered by soil from diurnal extremes and its overwintering is a sustained, months-long state of dormancy, we frame the molecular biology of winter survival as a question about a dormant subterranean organ and the survival strategy it deploys, not an immediate stress response.

Comment 4: Hypothesis stated explicitly

The hypothesis currently appears "hidden" within the text. I recommend using a standalone sentence or a paragraph starting with: "Here, we test the hypothesis that..."

Adopted. The Introduction now closes with explicit hypothesis sentences [L118]: "Here, we test the hypothesis that independently cold-adapted PACMAD lineages converge on a shared core winter proteome, defined as the set of orthogroups differentially abundant between summer-active and winter-dormant rhizomes in two or more of the five species examined. We further hypothesize that the magnitude of cold-induced proteomic responses is consistent with constraint imposed by the shared physiological requirements of freezing tolerance."

Methodology

Comment 5: "common-garden experiment"

For the phrase "in a common garden exposed to sustained sub-zero temperatures," use "common-garden experiment," which is the standard technical term.

Adopted. "Common garden" has been replaced with "common-garden experiment" throughout the manuscript and figure captions.

Comment 6: LC-MS/MS column and gradient details

There is a significant lack of detail in the main text regarding the LC-MS/MS setup. Please include: column specifications (brand, C18, length, diameter, particle size); whether a trap column was used; solvents and the exact chromatographic gradient.

Adopted. The LC description has been substantially expanded in S1 Methods (new subsection "Mass Spectrometric Acquisition"). Peptides were loaded onto a PepMap C-18 reversed-phase nano-trap column (5 µm, 100 µm × 20 mm; Dionex) and separated on a PepMap C-18 analytical

column (2 µm, 75 µm × 25 cm). Mobile phase A was 2% acetonitrile in 0.1% aqueous formic acid; mobile phase B was 95% acetonitrile in 0.1% formic acid. The 162-min run used a 120-min gradient from 5% to 33% B, an 8-min ramp to 90% B, a 9-min hold at 90% B, and a 25-min re-equilibration. Flow rate was 300 nL/min and the column oven was maintained at 35 °C.

Comment 7: MS3 acquisition parameters

Provide the collision energy (HCD/CID), injection time, and precursor isolation windows for TMT. Without these, the MS3 data cannot be replicated.

Full MS2 and MS3 acquisition parameters have been added to S1 Methods. MS2 fragmentation used CID in the linear ion trap (NCE 30%, isolation width 0.7 m/z, max injection time 50 ms, AGC target 1 × 10⁴). MS3 fragmentation of the top 10 SPS precursors used HCD in the Orbitrap (NCE 55%, isolation width 1.1 m/z, max injection time 120 ms, AGC target 1 × 10⁵). The Real-Time Search node searched MS2 spectra against the species-specific reference proteome with TMTpro and carbamidomethyl as static modifications and methionine oxidation as a variable modification, with up to one missed cleavage and two variable modifications per peptide. No inclusion list was used.

Comment 8: QC and mass calibration

Was any quality control or mass calibration monitoring performed during the runs?

Yes. The new "Instrument Quality Control and Calibration" subsection in S1 Methods describes the workflow: mass accuracy was monitored using the protonated hexakis(2,2-dimethoxypropane)cyclodisiloxane lock-mass ion at m/z 445.120029 with a 5 ppm SD tolerance prior to each sample injection; when the lock-mass mass error exceeded 5 ppm, the instrument was recalibrated using the Pierce FlexMix calibration solution. Escherichia coli tryptic digest and BSA were run as quality-assurance samples prior to each sample series, with instrument performance evaluated via the TIC, base peak chromatograms of the E. coli digest, and extracted ion chromatograms of representative BSA peptides.

Comment 9: FASTA sources and contaminant database

Specify the source of the FASTA files for each species (e.g., Phytozome, NCBI, UniProt), the number of entries per database, and if a common contaminant database was included.

The per-species FASTA sources, version codes, and Data Availability URLs (MaizeGDB and JGI Data Portal) were already provided in the originally submitted manuscript ("Peptide Mapping and Reference Proteome Assignment" subsection and Data Availability section). We have now added: (i) [L249] per-database protein-entry counts (197,551 for A. gerardi; 67,789 for M. sinensis; 95,264 for P. virgatum; 123,723 for S. nutans; 65,151 for T. dactyloides); and (ii) [L255] explicit confirmation that all databases were supplemented with the cRAP common contaminants database for the Sequest HT search.

Comment 10: FDR and TMT quantification

It is unclear if FDR filters were applied at both peptide and protein levels (standard <1%). Clarify the quantification algorithm: were raw TMT intensities used, or was protein-level normalization applied?

The originally submitted M&M states that Proteome Discoverer 2.5 was used with the Sequest HT engine, that peptide-spectral matches were filtered at 1% FDR (Percolator), that protein identifications required at least two distinct peptides, that protein quantification was based on summed reporter ion intensities of unique and razor peptides, that intensities were normalized using the "total peptide amount" method, and that statistical testing used ANOVA followed by Tukey HSD with Benjamini-Hochberg correction. To address the reviewer's specific questions: (i) FDR filters were applied at both peptide and protein levels (1% strict FDR), now stated explicitly in S1 Methods; (ii) reporter ions were quantified at the MS3 level (HCD, FTMS analyzer, 20 ppm integration tolerance), filtered at co-isolation threshold 50%, average reporter S/N at least 10, and SPS mass matches at least 65%, with reporter intensities of unique + razor peptides summed per protein and normalized across channels by Total Peptide Amount. Modified peptides (deamidated N/Q) were excluded from pairwise ratio calculation. Full parameter list is now provided in S1 Methods.

Results and Discussion

Comment 11: S. nutans n=2 — sensitivity analysis

Winter mortality reduced the biological replicates of S. nutans to n=2. How did this affect statistical power? Have the authors performed a sensitivity analysis to ensure this species is not distorting orthogroup correlations? Clarify how intra-group variance was handled.

We thank the reviewer; this concern overlaps with Reviewer 2's major comment on sample-size imbalance, and we address both together. The originally submitted Results already reported a sensitivity analysis: "This result was robust to the exclusion of S. nutans, which had fewer biological replicates (n = 2 per season; P = 0.002)." To address the reviewer's concern more fully, we have added a Limitations sentence explicitly framing the replication imbalance: S. nutans yields wider per-species confidence intervals than the other species; orthogroup-level fold-change summarization buffers, but does not eliminate, replication-driven variance; and the Tripsacum genus is represented as a single collapsed entry in cross-species tests to avoid pseudo-replication.

Comment 12: Post-translational buffering

The proteome being more conserved than the transcriptome is not fully explored. Discussing the concept of "post-translational buffering" would enrich the manuscript.

Adopted. The originally submitted Discussion already invoked this framework via Cope et al. (ref [29]), explaining that protein abundances are under stronger stabilizing selection than mRNA abundances. We have now strengthened this paragraph [L623] connecting the buffering argument specifically to the dormancy-organ context: "Such buffering is plausibly under particularly strong selection in an overwintering rhizome, which must maintain a functional, dormancy-competent proteome over months rather than minutes."

Comment 13: Subterranean / soil-insulated context

Emphasize that rhizomes are subterranean organs. Soil insulation provides a different environment than leaves; conservation findings might be specific to this "dormancy organ."

Adopted. We have added a Discussion paragraph that makes this point explicitly: soil thermally buffers below-ground organs against the diurnal extremes that aerial tissues experience, and the dormant rhizome inhabits a relatively stable, sustained sub-zero environment rather than the acute, fluctuating cold stress imposed on leaves. The conservation patterns reported here are therefore specific to a dormancy organ and may not generalize to leaf-level cold-shock responses.

Comment 14: LEA3 abundance vs. 11-aa repeat number

It is unclear if total LEA3 abundance compensates for the lower number of repeats in sensitive species. Does maize (sensitive) produce more copies of a "short" protein or fewer copies?

We thank the reviewer for this perceptive question. The originally submitted Discussion identified three factors shaping cryoprotective efficacy (amphipathic helix length per protein, total protein abundance via paralog accumulation, and repeat composition), and noted that Andropogon gerardi and Panicum virgatum accumulate multiple LEA3 paralogs while maize and M. giganteus do not. To make this answer explicit, we have added: "Maize encodes a single LEA3 ortholog, with no copy-number expansion to offset the shorter, more hydrophobic protein architecture." The single-paralog result was confirmed by DIAMOND BLASTp searches against the maize B73 NAM v5 reference proteome.

Comment 15: UpSet / Venn of shared proteins

Add a graphic (UpSet plot or Venn diagram) showing the exact intersection of shared proteins among the five species.

An UpSet plot of shared DAP intersections across species is provided as S6 Fig. We agree the figure deserves more prominent treatment and have added an explicit Results-section sentence pointing to it [L396]: "The full pattern of pairwise, triple, quadruple, and five-way DAP intersections across species is shown in S6 Fig."

Comment 16: Pathway breadth visualization

A visualization (e.g., Bubble Plot) showing which metabolic pathways are most conserved across species beyond LEA3 would add significant value.

Fig 3a in the originally submitted manuscript already presents this information: a mosaic plot of cold-related functional categories across all five species, showing which pathways (protein folding, metabolism/osmoregulation, detoxification/ROS scavenging, lipid metabolism, membrane stability, and others) are conserved versus species-specific in their relative investment. The mosaic plot was chosen over a bubble/dotplot because it integrates relative abundance and category presence within each species in a single panel. The Results narrative identifies the conserved categories beyond LEA3 (HSPs, ROS scavenging, putative chitinase-like antifreeze proteins) and discusses lineage-specific allocation.

General

Comment 17: Active voice

The manuscript relies heavily on the passive voice. Recommend active voice to make the text more direct and engaging.

Adopted in part. We have made selective active-voice conversions where the text read as overly impersonal. We have retained passive voice in Methods sections covering routine protocols (sample lysis, digestion, mass-spectrometric acquisition), where it is the field-standard convention. We hope this approach is acceptable.

Comment 18: Conservation outliers

Explore "Conservation Outliers" — proteins highly responsive in only one species despite belonging to conserved families — to provide a counterpoint to the "constrained evolution" thesis.

Adopted. We have added a Discussion paragraph and a new S13 Table identifying conservation outliers: orthogroups whose response in a single species substantially exceeds the cross-species median, despite belonging to functional families otherwise represented in two or more species. Notable examples highlighted in the text include a Temperature-Induced Lipocalin orthogroup elevated specifically in Tripsacum, a chitinase-like GH18 protein elevated in A. gerardi, and ureidoglycolate hydrolase elevated only in M. giganteus. We highlight these outliers as biologically meaningful exceptions rather than as a counter-thesis; the constrained-magnitude pattern is not contingent on their presence.

Reviewer 2

Major comments

Major Comment 1: "Constrained evolution" overstated

The authors claim that protein-level responses reflect "constrained evolution." This claim seems overstated. While the observed correlations are compelling, they do not uniquely imply evolutionary constraint.

We agree, and have made this the central interpretive revision. The originally submitted Abstract used "is consistent with evolutionary constraints on protein-level response magnitude" rather than asserting causation; we have now extended this consistency framing to the body. Two remaining instances of causal language ("is governed by") have been softened to "is consistent with." We have not removed the mechanistic hypothesis but now present it as one of several non-mutually-exclusive explanations (see Major Comment 2). The manuscript title has been updated from "Constrained evolution of..." to "Evolutionary constraint on..." to reflect the consistency framing throughout the body, now describing constraint as a property of the proteome we observed rather than a sweeping evolutionary claim.

Major Comment 2: Alternative explanations not sufficiently discussed

The alternative explanations such as shared physiological limits, environmental uniformity, and statistical thresholds are not sufficiently discussed.

Adopted. The Discussion now contains a dedicated paragraph enumerating three non-mutually-exclusive explanations for the observed cross-species correlations [L590]: (i) shared physiological limits — the biophysical demands of freezing tolerance impose a narrow set of viable molecular responses, generating cross-species correlation independently of evolutionary history; (ii) environmental uniformity — the common-garden experiment exposes all species to identical thermal cues, which alone can elicit correlated responses from independently evolved sensing pathways; and (iii) statistical-threshold artifacts — the |log2FC| at least 1 selection criterion preferentially retains proteins with strong, reproducible responses, biasing toward shared signal. We discuss honestly which alternatives our controls (magnitude-matched permutation; Brownian-motion permulation) address, and which they do not.

Major Comment 3: Sample-size imbalance

There seems to be imbalance in sample size across species, for example extensive Tripsacum sampling vs. limited replication in other species, which might challenge the identification of differentially abundant proteins and shared responses.

We address this concern jointly with Reviewer 1 Comment 11. Tripsacum accessions (Trip19, Trip22) were collapsed into a single Tripsacum entry for cross-species analyses to avoid pseudo-replication. Per-species DAP calling uses replication-aware tests; orthogroup-level fold-change summarization buffers some of the imbalance. The originally submitted Results [L584] documents that the headline cross-species correlation is robust to exclusion of S. nutans (the species with reduced replication; P = 0.002), and we have added an explicit Limitations sentence framing the replication imbalance.

Major Comment 4: LEA3 not experimentally validated

The key findings, particularly regarding LEA3, were not experimentally validated. The authors may rephrase the conclusions and acknowledge the limitation.

Adopted. The originally submitted Discussion includes the caveat that "freezing survival involves the coordinated action of numerous protective mechanisms, and restoring a single protein's architecture in a freezing-sensitive species would be unlikely to confer full tolerance." To make the validation gap fully explicit, we have softened LEA3 functional language throughout the Discussion and added a closing sentence [L664]: "Direct experimental validation of the proposed LEA3 contribution, for example through heterologous expression, repeat-number perturbation, or in vitro cryoprotection assays, is required to establish causation and is beyond the scope of the present study."

Minor comments

Minor Comment 1: Define key terms

Define key terms such as "core winter proteome" and "constraint on response magnitude."

Adopted. The Introduction now defines "core winter proteome" on first body use as "the set of orthogroups that are differentially abundant between summer-active and winter-dormant rhizomes in two or more of the five species examined." "Constraint on response magnitude" is defined operationally in the Discussion as "a higher cross-species correlation in cold-induced fold-change among shared cold-responsive orthogroups than among proteins responsive in at most one species."

Minor Comment 2: |log2FC| ≥ 1 threshold

Clarify threshold selection (|log2FC| ≥ 1).

This is already addressed in the originally submitted M&M, where we justify the |log2FC| at least 1 cutoff as a stringent threshold appropriate for a multi-species field study with inherent biological variability, prioritizing high-confidence candidates over minor fluctuations that may reflect transient environmental noise. The same paragraph notes that this approach addresses the statistical power imbalance between the deeply sampled Tripsacum dataset and the smaller per-species datasets.

Minor Comment 3: Batch correction and normalization detail

Provide more detail on batch correction and normalization.

The Methods describe within-plex normalization (Total Peptide Amount in PD 2.5) and the multi-batch Tripsacum 2022 strategy (within-batch testing followed by Fisher's-method P-value combination, retention of proteins quantified in at least 3 of 4 batches). Cross-species normalization rests on orthogroup-level fold-change summarization rather than direct cross-plex abundance correction. The full implementation is available in the GitHub repository linked in Data Availability.

Attachment Submitted filename: Response_to_Reviewers_clean.docx

# Peer review: Decision Letter 1

**DOI:** 10.1371/journal.pone.0358458.r003

**Authors:** Amaranatha Reddy Vennapusa

22 Aug 2026

PONE-D-26-14249R1

Evolutionary constraint on a core winter proteome across independently cold-adapted PACMAD grasses

PLOS One

Dear Dr. Oren,

Thank you for submitting your manuscript to PLOS One. After careful consideration, we feel that it has merit but does not fully meet PLOS One’s publication criteria as it currently stands. Therefore, we invite you to submit a revised version of the manuscript that addresses the points raised during the review process.

Please submit your revised manuscript by Oct 06 2026 11:59PM. If you will need more time than this to complete your revisions, please reply to this message or contact the journal office at plosone@plos.org. When you're ready to submit your revision, log on to https://www.editorialmanager.com/pone/ and select the 'Submissions Needing Revision' folder to locate your manuscript file.

Please include the following items when submitting your revised manuscript:

- A letter that responds to each point raised by the academic editor and reviewer(s). You should upload this letter as a separate file labeled 'Response to Reviewers'.
- A marked-up copy of your manuscript that highlights changes made to the original version. You should upload this as a separate file labeled 'Revised Manuscript with Track Changes'.
- An unmarked version of your revised paper without tracked changes. You should upload this as a separate file labeled 'Manuscript'.

If you would like to make changes to your financial disclosure, please include your updated statement in your cover letter. Guidelines for resubmitting your figure files are available below the reviewer comments at the end of this letter.

If applicable, we recommend that you deposit your laboratory protocols in protocols.io to enhance the reproducibility of your results. Protocols.io assigns your protocol its own identifier (DOI) so that it can be cited independently in the future. For instructions see: https://journals.plos.org/plosone/s/submission-guidelines#loc-laboratory-protocols. Additionally, PLOS One offers an option for publishing peer-reviewed Lab Protocol articles, which describe protocols hosted on protocols.io. Read more information on sharing protocols at https://plos.org/protocols?utm_medium=editorial-email&utm_source=authorletters&utm_campaign=protocols.

As the corresponding author, your ORCID iD is verified in the submission system and will appear in the published article. PLOS supports the use of ORCID, and we encourage all coauthors to register for an ORCID iD and use it as well. Please encourage your coauthors to verify their ORCID iD within the submission system before final acceptance, as unverified ORCID iDs will not appear in the published article. *Only*  the individual author can complete the verification step; PLOS staff *cannot*  verify ORCID iDs on behalf of authors.

We look forward to receiving your revised manuscript.

Kind regards,

Amaranatha Reddy Vennapusa

Guest Editor

PLOS One

**Journal Requirements:**

1. If the reviewer comments include a recommendation to cite specific previously published works, please review and evaluate these publications to determine whether they are relevant and should be cited. There is no requirement to cite these works unless the editor has indicated otherwise.

2. Please review your reference list to ensure that it is complete and correct. If you have cited papers that have been retracted, please include the rationale for doing so in the manuscript text, or remove these references and replace them with relevant current references. Any changes to the reference list should be mentioned in the rebuttal letter that accompanies your revised manuscript. If you need to cite a retracted article, indicate the article’s retracted status in the References list and also include a citation and full reference for the retraction notice.

**Additional Editor Comments:**

Please ensure that the affiliations, email for the corresponding author (Elad Oren) are correctly specified. Should it be Newe Ya’ar Research Center, ARO, Israel, Cornell University, or both?

In the methods section, please specify which plant species belong to each of the following clades: Panicoideae, Aristidoideae, Chloridoideae, Micrairoideae, Arundinelloideae, and Danthonioideae (PACMAD).

Maintain a uniform format when citing the table or figure numbers, and check the journal guidelines. Should it be Supporting Information S1 Methods, S1 Fig, or Fig S1?

Please provide citations for the F₂ seedlings used in RNA sequencing and RNA extraction, along with a brief description of the method.

Additionally, cite the references for the protein extraction and digestion method.

Ensure that the referencing format is consistent throughout both the manuscript and supporting information—either numbering or author names. For example, line 276: should it be "Cope et al. (2025)" or a reference number?

Lastly, did the authors perform any qRT-PCR or other functional validation studies to confirm the function or variation of specific LEA3 genes or proteins?

[Note: HTML markup is below. Please do not edit.]

Reviewers' comments:

Reviewer's Responses to Questions

**Comments to the Author**

1. If the authors have adequately addressed your comments raised in a previous round of review and you feel that this manuscript is now acceptable for publication, you may indicate that here to bypass the “Comments to the Author” section, enter your conflict of interest statement in the “Confidential to Editor” section, and submit your "Accept" recommendation.

Reviewer #1: All comments have been addressed

Reviewer #2: All comments have been addressed

**********

2. Is the manuscript technically sound, and do the data support the conclusions?

The manuscript must describe a technically sound piece of scientific research with data that supports the conclusions. Experiments must have been conducted rigorously, with appropriate controls, replication, and sample sizes. The conclusions must be drawn appropriately based on the data presented.

Reviewer #1: Yes

Reviewer #2: Yes

**********

3. Has the statistical analysis been performed appropriately and rigorously?

Reviewer #1: Yes

Reviewer #2: Yes

**********

4. Have the authors made all data underlying the findings in their manuscript fully available?

The PLOS Data policy requires authors to make all data underlying the findings described in their manuscript fully available without restriction, with rare exception (please refer to the Data Availability Statement in the manuscript PDF file). The data should be provided as part of the manuscript or its supporting information, or deposited to a public repository. For example, in addition to summary statistics, the data points behind means, medians and variance measures should be available. If there are restrictions on publicly sharing data—e.g. participant privacy or use of data from a third party—those must be specified.

Reviewer #1: Yes

Reviewer #2: (No Response)

**********

5. Is the manuscript presented in an intelligible fashion and written in standard English?

PLOS ONE does not copyedit accepted manuscripts, so the language in submitted articles must be clear, correct, and unambiguous. Any typographical or grammatical errors should be corrected at revision, so please note any specific errors here.

Reviewer #1: Yes

Reviewer #2: Yes

**********

6. Review Comments to the Author

Please use the space provided to explain your answers to the questions above. You may also include additional comments for the author, including concerns about dual publication, research ethics, or publication ethics. (Please upload your review as an attachment if it exceeds 20,000 characters)

Reviewer #1: The revised manuscript makes a significant contribution to comparative plant proteomics, demonstrating that the rhizome’s seasonal responses to freezing are evolutionarily conserved through post-translational protective mechanisms, despite transcriptomic divergence. The authors have addressed all peer-review comments with exemplary rigor. From a methodological standpoint, the text now ensures absolute reproducibility by fully detailing the LC-MS/MS parameters. Statistically, the limitation of the small sample size for Sorghastrum nutans was robustly addressed and validated through a transparent sensitivity analysis. Mechanistically, the structural dissection of the LEA3 protein brilliantly explains the phenotypic differences in cold tolerance among the lines. The article is recommended for acceptance in its current form.

Reviewer #2: (No Response)

**********

7. PLOS authors have the option to publish the peer review history of their article (what does this mean?). If published, this will include your full peer review and any attached files.

If you choose “no”, your identity will remain anonymous but your review may still be made public.

**Do you want your identity to be public for this peer review?**  For information about this choice, including consent withdrawal, please see our Privacy Policy.

Reviewer #1: No

Reviewer #2: **Yes:**  Srinivasan Balamurugan

**********

[NOTE: If reviewer comments were submitted as an attachment file, they will be attached to this email and accessible via the submission site. Please log into your account, locate the manuscript record, and check for the action link "View Attachments". If this link does not appear, there are no attachment files.]

To ensure your figures meet our technical requirements, please review our figure guidelines: https://journals.plos.org/plosone/s/figures

You may also use PLOS’s free figure tool, NAAS, to help you prepare publication quality figures: https://journals.plos.org/plosone/s/figures#loc-tools-for-figure-preparation.

NAAS will assess whether your figures meet our technical requirements by comparing each figure against our figure specifications.

# Author comment: Author response to Decision Letter 2

**DOI:** 10.1371/journal.pone.0358458.r004

24 Aug 2026

Comment 1:

Please ensure that the affiliations, email for the corresponding author (Elad Oren) are correctly specified. Should it be Newe Ya'ar Research Center, ARO, Israel, Cornell University, or both?

Both. We have corrected this. E.O. is now listed with both affiliations, and the correspondence address and email have been updated accordingly.

Comment 2:

In the methods section, please specify which plant species belong to each of the following clades: Panicoideae, Aristidoideae, Chloridoideae, Micrairoideae, Arundinelloideae, and Danthonioideae (PACMAD).

Adopted, and we thank the editor for raising this. We have added a clarification to the opening of the Materials and Methods. While all sampled taxa fall within Panicoideae, the PACMAD framing describes the evolutionary context of repeated cold adaptation, not the taxonomic breadth of our sampling. We have also corrected the subfamily name in the Abstract and Introduction to Arundinoideae.

Comment 3:

Maintain a uniform format when citing the table or figure numbers, and check the journal guidelines. Should it be Supporting Information S1 Methods, S1 Fig, or Fig S1?

Corrected according to the PLOS ONE convention.

Comment 4:

Please provide citations for the F₂ seedlings used in RNA sequencing and RNA extraction, along with a brief description of the method. Additionally, cite the references for the protein extraction and digestion method.

Adopted. We now cite El-Walid et al. [32] for the F₂ germplasm, crossing design, and freezing screen, and have expanded the "F₂ Seedlings for RNA sequencing" subsection with a brief description of the growth, cold-acclimation, and screening conditions; this companion preprint was posted after our original submission, which is why it could not be cited previously. We have also clarified the sampling sequence. RNA extraction follows Gault et al. [43], as before. For the proteomics, we have extended the existing citation to Fu et al. [34], which describes the S-Trap digestion, TMTpro labeling, and fractionation strategy used here, and added Isaacson et al. [33] for the phenol/ammonium acetate–methanol extraction. Full buffer compositions remain in S1 Methods.

Comment 5:

Ensure that the referencing format is consistent throughout both the manuscript and supporting information—either numbering or author names. For example, line 276: should it be "Cope et al. (2025)" or a reference number?

Corrected. The manuscript uses the PLOS ONE Vancouver numbered format throughout.

Comment 6:

Lastly, did the authors perform any qRT-PCR or other functional validation studies to confirm the function or variation of specific LEA3 genes or proteins?

No. No qRT-PCR, heterologous expression, or in vitro cryoprotection assays were performed. The study is a comparative quantitative-proteomics survey, and our LEA3 findings are framed accordingly as observations of cross-species abundance and structural architecture rather than functional claims; following Reviewer 2's Major Comment 4 in the previous round, we softened the functional language throughout the Discussion and stated explicitly that direct validation is required to establish causation and is beyond the present scope [L677].

Attachment Submitted filename: Response_to_Reviewers_R2_clean.docx

# Editor report: Decision Letter 2

**DOI:** 10.1371/journal.pone.0358458.r005

**Authors:** Amaranatha Reddy Vennapusa

2 Sep 2026

Evolutionary constraint on a core winter proteome across independently cold-adapted PACMAD grasses

PONE-D-26-14249R2

Dear Dr. Elad Oren

We’re pleased to inform you that your manuscript has been judged scientifically suitable for publication and will be formally accepted for publication once it meets all outstanding technical requirements.

Within one week, you’ll receive an e-mail detailing the required amendments. When these have been addressed, you’ll receive a formal acceptance letter and your manuscript will be scheduled for publication.

An invoice will be generated when your article is formally accepted. Please note, if your institution has a publishing partnership with PLOS and your article meets the relevant criteria, all or part of your publication costs will be covered. Please make sure your user information is up-to-date by logging into Editorial Manager at Editorial Manager® and clicking the ‘Update My Information' link at the top of the page. For questions related to billing, please contact billing support.

If your institution or institutions have a press office, please notify them about your upcoming paper to help maximize its impact. If they’ll be preparing press materials, please inform our press team as soon as possible -- no later than 48 hours after receiving the formal acceptance. Your manuscript will remain under strict press embargo until 2 pm Eastern Time on the date of publication. For more information, please contact onepress@plos.org.

Kind regards,

Amaranatha Reddy Vennapusa

Guest Editor

PLOS One

Additional Editor Comments (optional):

Thank you for addressing the comments and submitting the rebuttal letter.

Reviewers' comments:

# Editor report: Acceptance letter

**DOI:** 10.1371/journal.pone.0358458.r006

**Authors:** Amaranatha Reddy Vennapusa

PONE-D-26-14249R2

PLOS One

Dear Dr. Oren,

I'm pleased to inform you that your manuscript has been deemed suitable for publication in PLOS One. Congratulations! Your manuscript is now being handed over to our production team.

At this stage, our production department will prepare your paper for publication. This includes ensuring the following:

* All references, tables, and figures are properly cited

* All relevant supporting information is included in the manuscript submission,

* There are no issues that prevent the paper from being properly typeset

You will receive further instructions from the production team, including instructions on how to review your proof when it is ready. Please keep in mind that we are working through a large volume of accepted articles, so please give us a few days to review your paper and let you know the next and final steps.

Lastly, if your institution or institutions have a press office, please let them know about your upcoming paper now to help maximize its impact. If they'll be preparing press materials, please inform our press team within the next 48 hours. Your manuscript will remain under strict press embargo until 2 pm Eastern Time on the date of publication. For more information, please contact onepress@plos.org.

You will receive an invoice from PLOS for your publication fee after your manuscript has reached the completed accept phase. If you receive an email requesting payment before acceptance or for any other service, this may be a phishing scheme. Learn how to identify phishing emails and protect your accounts at https://explore.plos.org/phishing.

If we can help with anything else, please email us at customercare@plos.org.

Thank you for submitting your work to PLOS One and supporting open access.

Kind regards,

PLOS One Editorial Office Staff

on behalf of

Dr. Amaranatha Reddy Vennapusa

Guest Editor

PLOS One
